# KIR^+^CD8^+^ T cells suppress pathogenic T cells and are active in autoimmune diseases and COVID-19

**DOI:** 10.1126/science.abi9591

**Published:** 2022-03-08

**Authors:** Jing Li, Maxim Zaslavsky, Yapeng Su, Jing Guo, Michael J. Sikora, Vincent van Unen, Asbjørn Christophersen, Shin-Heng Chiou, Liang Chen, Jiefu Li, Xuhuai Ji, Julie Wilhelmy, Alana M. McSween, Brad A. Palanski, Venkata Vamsee Aditya Mallajosyula, Nathan A. Bracey, Gopal Krishna R. Dhondalay, Kartik Bhamidipati, Joy Pai, Lucas B. Kipp, Jeffrey E. Dunn, Stephen L. Hauser, Jorge R. Oksenberg, Ansuman T. Satpathy, William H. Robinson, Cornelia L. Dekker, Lars M. Steinmetz, Chaitan Khosla, Paul J. Utz, Ludvig M. Sollid, Yueh-Hsiu Chien, James R. Heath, Nielsen Q. Fernandez-Becker, Kari C. Nadeau, Naresha Saligrama, Mark M. Davis

**Affiliations:** ^1^ Institute of Immunity, Transplantation and Infection, Stanford University School of Medicine, Stanford, CA, USA.; ^2^ Program in Computer Science, Stanford University, Stanford, CA, USA.; ^3^ Institute for Systems Biology, Seattle, WA, USA.; ^4^ Department of Microbiology and Immunology, Stanford University School of Medicine, Stanford, CA, USA.; ^5^ Department of Genetics, Stanford University School of Medicine, Stanford, CA, USA.; ^6^ K.G. Jebsen Coeliac Disease Research Centre, University of Oslo, Oslo, Norway.; ^7^ Institute of Clinical Medicine, University of Oslo, Oslo, Norway.; ^8^ Department of Immunology, University of Oslo, Oslo, Norway.; ^9^ The Howard Hughes Medical Institute, Stanford University School of Medicine, Stanford, CA, USA.; ^10^ Human Immune Monitoring Center, Stanford University School of Medicine, Stanford, CA, USA.; ^11^ Department of Chemistry, Stanford University, Stanford, CA, USA.; ^12^ Sean N. Parker Center for Allergy and Asthma Research, Department of Medicine, Stanford University, Stanford, CA, USA.; ^13^ Program in Immunology, Stanford University School of Medicine, Stanford, CA, USA.; ^14^ Division of Neuroimmunology, Department of Neurology and Neurological Sciences, Stanford University School of Medicine, Stanford, CA, USA.; ^15^ Department of Neurology and UCSF Weill Institute for Neurosciences, University of California, San Francisco, CA, USA.; ^16^ Department of Pathology, Stanford University School of Medicine, Stanford, CA, USA.; ^17^ VA Palo Alto Health Care System, Palo Alto, CA, USA.; ^18^ Division of Immunology and Rheumatology, Department of Medicine, Stanford University, Stanford, CA, USA.; ^19^ Department of Pediatrics, Stanford University School of Medicine, Stanford, CA, USA.; ^20^ Stanford Genome Technology Center, Stanford University, Palo Alto, CA, USA.; ^21^ European Molecular Biology Laboratory (EMBL), Genome Biology Unit, Heidelberg, Germany.; ^22^ Department of Chemical Engineering, Stanford University, Stanford, CA, USA.; ^23^ Stanford ChEM-H, Stanford University, Stanford, CA, USA.; ^24^ Department of Immunology, Oslo University Hospital, Oslo, Norway.; ^25^ Department of Bioengineering, University of Washington, Seattle, WA, USA.; ^26^ Division of Gastroenterology and Hepatology, Department of Medicine, Stanford University, Stanford, CA, USA.; ^27^ Department of Microbiology and Immunology, Stanford University School of Medicine, Stanford, CA, USA.

## Abstract

In this work, we find that CD8^+^ T cells expressing inhibitory killer cell immunoglobulin-like receptors (KIRs) are the human equivalent of Ly49^+^CD8^+^ regulatory T cells in mice and are increased in the blood and inflamed tissues of patients with a variety of autoimmune diseases. Moreover, these CD8^+^ T cells efficiently eliminated pathogenic gliadin-specific CD4^+^ T cells from the leukocytes of celiac disease patients in vitro. We also find elevated levels of KIR^+^CD8^+^ T cells, but not CD4^+^ regulatory T cells, in COVID-19 patients, correlating with disease severity and vasculitis. Selective ablation of Ly49^+^CD8^+^ T cells in virus-infected mice led to autoimmunity after infection. Our results indicate that in both species, these regulatory CD8^+^ T cells act specifically to suppress pathogenic T cells in autoimmune and infectious diseases.

## Say a KIR-full goodbye to autoimmunity

Ly49^+^CD8^+^ T cells are a subset of CD8^+^ T cells that show immunoregulatory activity in mice. Li *et al*. report the existence of a similar CD8^+^ T cell subset in humans that expresses killer cell immunoglobulin-like receptors (KIRs), a functional parallel of the mouse Ly49 family (see the Perspective by Levescot and Bensussan). These cells, which can suppress self-reactive CD4^+^ T cells, were more abundant in patients with autoimmune conditions such as celiac disease, multiple sclerosis, and lupus, as well as in patients infected with influenza virus or severe acute respiratory syndrome coronavirus 2. When mice selectively deficient in Ly49^+^CD8^+^ T cells were infected with viruses, they showed normal antiviral immune responses but eventually developed symptoms of autoimmune disease. KIR^+^CD8^+^ T cells may therefore be an important therapeutic target for the control of autoimmune diseases such as “long COVID” that emerge after viral infections. —STS

## Abstract (structured)

**INTRODUCTION:**

Previous studies have identified Ly49^+^CD8^+^ T cells as a CD8^+^ T cell subset with regulatory functions in mice. These cells can suppress myelin oligodendrocyte glycoprotein (MOG)–specific pathogenic CD4^+^ T cells through their cytolytic activity and thereby ameliorate experimental autoimmune encephalomyelitis (EAE). However, whether a similar CD8^+^ regulatory T cell subset exists in humans and whether its suppressive activity extends beyond autoimmune diseases to play a more general role in peripheral tolerance remains to be determined.

**RATIONALE:**

Because killer cell immunoglobulin-like receptors (KIRs) are the evolutionary counterpart of the mouse Ly49 family in humans, we investigated whether KIR^+^CD8^+^ T cells are the phenotypic and functional equivalent of mouse Ly49^+^CD8^+^ T cells. We assessed the frequency of KIR^+^CD8^+^ T cells in human autoimmune and infectious diseases and analyzed their transcriptional profiles as well as T cell receptor (TCR) repertoires. Moreover, we developed an in vitro functional assay to test their regulatory functions on gliadin-specific disease-driving CD4^+^ T cells from patients with celiac disease (CeD) and to study the mechanisms of suppression. To further elucidate their role in infectious diseases, we analyzed the effects of selective ablation of their murine counterpart on the antiviral responses during infection and tissue pathology after resolution of viral infection.

**RESULTS:**

The frequency of KIR^+^CD8^+^ T cells was higher in the blood and inflamed tissues of patients with multiple autoimmune diseases compared with those of healthy controls. Furthermore, the increase of duodenal KIR^+^CD8^+^ T cells positively correlated with disease activity in CeD. In vitro, KIR^+^CD8^+^ T cells were able to specifically eliminate gliadin-specific pathogenic CD4^+^ T cells from the leukocytes of CeD patients through their cytotoxicity in a class I major histocompatibility complex (MHC)–dependent manner. Moreover, our RNA sequencing (RNA-seq) analysis revealed many similarities between human KIR^+^CD8^+^ T cells and mouse Ly49^+^CD8^+^ T cells, which suggests that KIR^+^CD8^+^ T cells are the functional and phenotypic equivalents of mouse Ly49^+^CD8^+^ T cells in humans. The expression of inhibitory KIR receptors appeared to suppress the activation and cytotoxic functions of KIR^+^CD8^+^ T cells. Moreover, elevated levels of KIR^+^CD8^+^ T cells, but not CD4^+^ regulatory T cells (T_regs_), were also observed in severe acute respiratory syndrome coronavirus 2 (SARS-CoV-2)– or influenza-infected patients and correlated with vasculitis in COVID-19 patients. Notably, expanded KIR^+^CD8^+^ T cells from healthy subjects and different diseases displayed shared phenotypes and similar TCR sequences as revealed by single-cell RNA-seq and TCR sequencing (TCR-seq). Finally, the selective depletion of Ly49^+^CD8^+^ T cells in virus-infected mice did not interfere with the antiviral responses but resulted in exacerbated autoimmunity after infection, demonstrating the critical role that this subset of CD8^+^ T_regs_ plays in controlling harmful self-reactivity in infectious diseases.

**CONCLUSION:**

We identify KIR^+^CD8^+^ T cells as an important regulatory T cell subset in humans. They are induced as part of the response during an autoimmune reaction or infection and may act as a negative feedback mechanism to specifically suppress the self-reactive or otherwise pathogenic cells without affecting the immune responses against pathogens. This subset of CD8^+^ T_regs_ appears to play an important role in maintaining peripheral tolerance, which is distinct from and likely complementary to that of CD4^+^ T_regs_. Our findings also provide insights into understanding the relationship between autoimmunity and infectious diseases and into the development of potential therapeutic approaches targeting KIR^+^CD8^+^ T cells to suppress undesirable self-reactivity in autoimmune disorders and infectious diseases.

## Teaser

Regulatory CD8^+^ T cells act to suppress pathogenic T cells in autoimmune and infectious diseases in mice and humans.

## Graphical abstract

**Figure:**
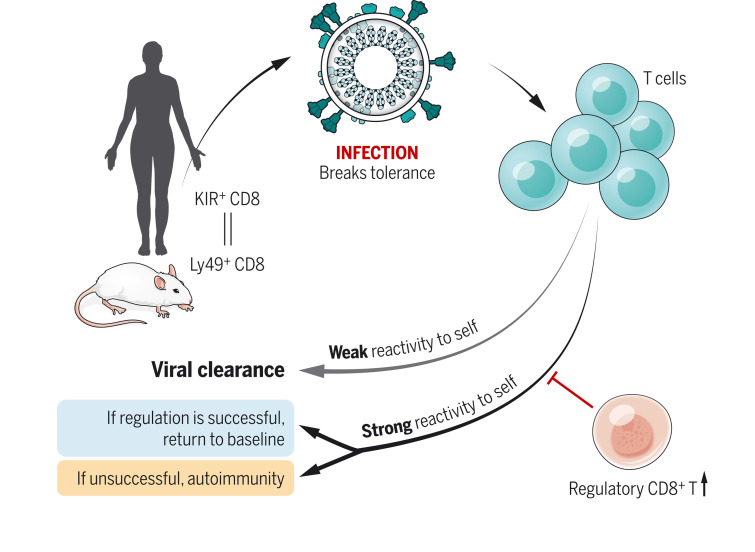
The proposed role of CD8^+^ regulatory T cells in peripheral tolerance. KIR^+^CD8^+^ T cells are the equivalent of mouse Ly49^+^CD8^+^ T cells in humans, with similar regulatory functions. During an infection, this kind of CD8^+^ regulatory T cell is induced and suppresses those CD4^+^ T cells with a strong reactivity to self, which may cause autoimmunity without interfering with the immune responses against pathogens.

Although most CD8^+^ T cells are geared toward the control of pathogen-infected or cancerous cells, there has been long-standing evidence in mice that a small subset can also suppress autoimmune responses (*1*). This regulatory function of CD8^+^ T cells was first implicated by the depletion of CD8^+^ T cells in experimental autoimmune encephalomyelitis (EAE), a mouse model of human multiple sclerosis (MS) (*2*, *3*). In particular, disruption of Qa-1-CD8 co-receptor binding in B6.Qa-1-D227K mice leads to spontaneous autoimmune diseases (*4*). The Ly49 family of inhibitory C-type lectin-like receptors, which are ubiquitous on natural killer (NK) cells, were identified as specific surface markers for this regulatory CD8^+^ T cell subset (*5*). Moreover, the transcription factor Helios is an essential control element for their differentiation and function in mice (*6*). Recently, our research group found that clonally expanded CD8^+^ T cells in EAE recognized peptides bound to H2-D^b^ and that these peptides stimulate Ly49^+^CD8^+^ regulatory T cells and suppress disease (*7*). This extended the original observations beyond Qa-1 to encompass classical class I major histocompatibility complex (MHC) interactions, suggesting a general mechanism of peripheral tolerance. In this work, we identify CD8^+^ T cells expressing inhibitory killer cell immunoglobulin-like receptors (KIRs)—the functional counterpart of the mouse Ly49 family in humans (*8*)—as a regulatory CD8^+^ T cell subset in humans that suppresses pathogenic CD4^+^ T cells in celiac disease (CeD) and likely other autoimmune disorders and infectious diseases as well.

## Increased KIR^+^CD8^+^ T cells in human autoimmune diseases

**Fig. 1.**
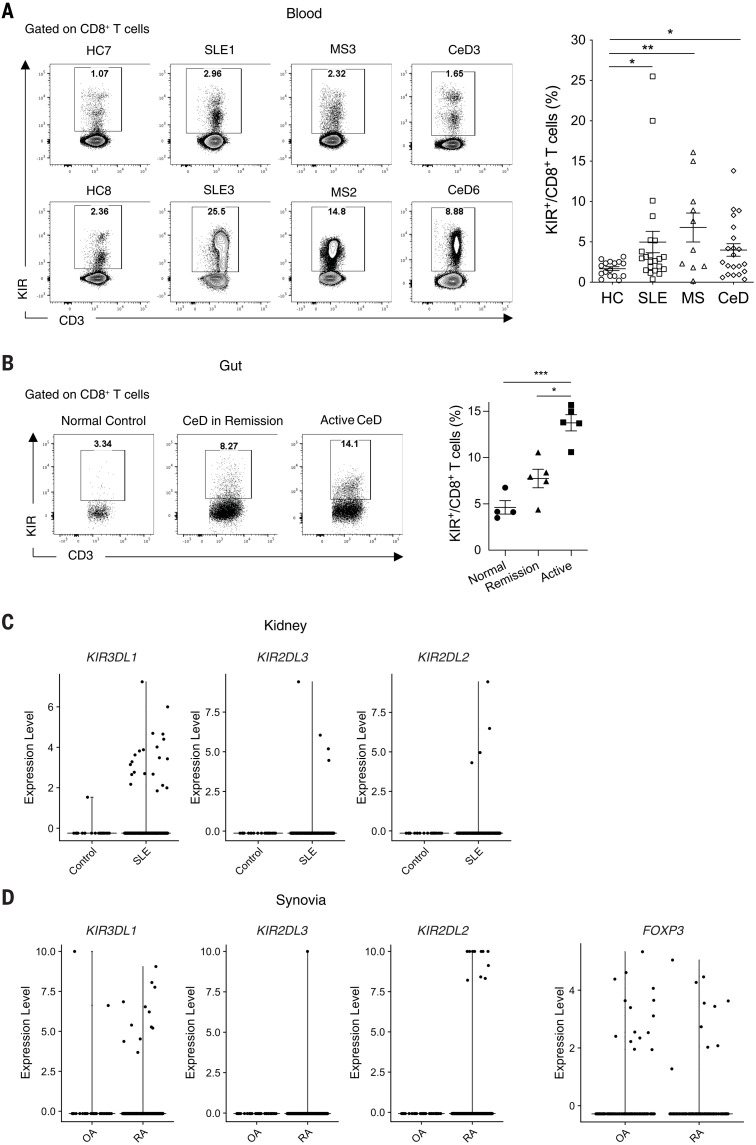
Increased KIR^+^CD8^+^ T cells in patients with autoimmune diseases. (**A**) Representative contour plots and a summary histogram showing the frequency of KIR^+^CD8^+^ T cells in the peripheral blood of HCs (*N* = 16) and patients with SLE (*N* = 22), MS (*N* = 10), or CeD (*N* = 21) analyzed by flow cytometry. KIR^+^ cells were detected by PE-conjugated antibodies against KIR2DL1 (clone no. 143211), KIR2DL2/L3 (Dx27), KIR2DL5 (UP-R1), KIR3DL1 (Dx9), and KIR3DL2 (clone no. 539304). *P < 0.05; **P < 0.01; Kruskal-Wallis test followed by multiple comparisons test. (**B**) Representative contour plots and a summary histogram showing the frequency of KIR^+^CD8^+^ T cells in the duodenum of normal controls (*N* = 4), CeD in remission (*N* = 5), and active CeD (*N* = 5) analyzed by flow cytometry. *P < 0.05; ***P < 0.001; Kruskal-Wallis test followed by multiple comparisons test. (**C**) Expression of *KIR* transcripts (*KIR3DL1*, *KIR2DL3*, and *KIR2DL2*) in CD8^+^ T cells from healthy kidneys (control, *N* = 6) versus SLE nephritis kidneys (*N* = 20). (**D**) Expression of *KIR* transcripts (*KIR3DL1*, *KIR2DL3*, and *KIR2DL2*) in synovial CD8^+^ T cells and expression of *FOXP3* in synovial CD4^+^ T cells from RA (*N* = 18) and OA (*N* = 3).

Both mouse Ly49 and human KIR receptors are known to bind to class I MHC molecules (*8*). They typically have immunoreceptor tyrosine-based inhibitory motifs (ITIMs) in their cytoplasmic tails and are ubiquitously expressed on NK cells as well as on a small subset (1 to 5%) of CD8^+^ T cells (*5*). Therefore, we analyzed CD8^+^ T cells expressing inhibitory KIRs (which we refer to as KIR^+^CD8^+^ T cells) (*9*, *10*) in the peripheral blood of patients with autoimmune diseases and age- and gender-matched healthy controls (HCs). KIR3DL1 and KIR2DL3 were the two major KIR subtypes expressed by human CD8^+^ T cells (fig. S1). The frequency of KIR^+^CD8^+^ T cells was significantly increased in the blood of patients with MS, systemic lupus erythematosus (SLE), or CeD compared with the blood of HCs (Fig. 1A).

Next, we investigated whether KIR^+^CD8^+^ T cells were also present in the inflamed tissues of patients with these diseases. CeD patients with active disease had higher levels of KIR^+^CD8^+^ T cells in the gut compared with those in remission (on a gluten-free diet) as well as the non-CeD controls (Fig. 1B), which indicates a strong correlation of KIR^+^CD8^+^ T cell levels with disease severity. Additionally, the number of KIR^+^CD8^+^ T cells was markedly increased in the kidneys of patients with SLE compared with healthy kidneys (Fig. 1C) and in the synovial tissues of rheumatoid arthritis (RA) patients compared with those with osteoarthritis (OA), which is not thought to be an autoimmune disease. By contrast, the frequencies of synovial FOXP3^+^CD4^+^ regulatory T cells (T_regs_) were similar between RA and OA patients (Fig. 1D).

## KIR^+^CD8^+^ T cells are the functional and phenotypic equivalent of mouse Ly49^+^CD8^+^ T cells

**Fig. 2.**
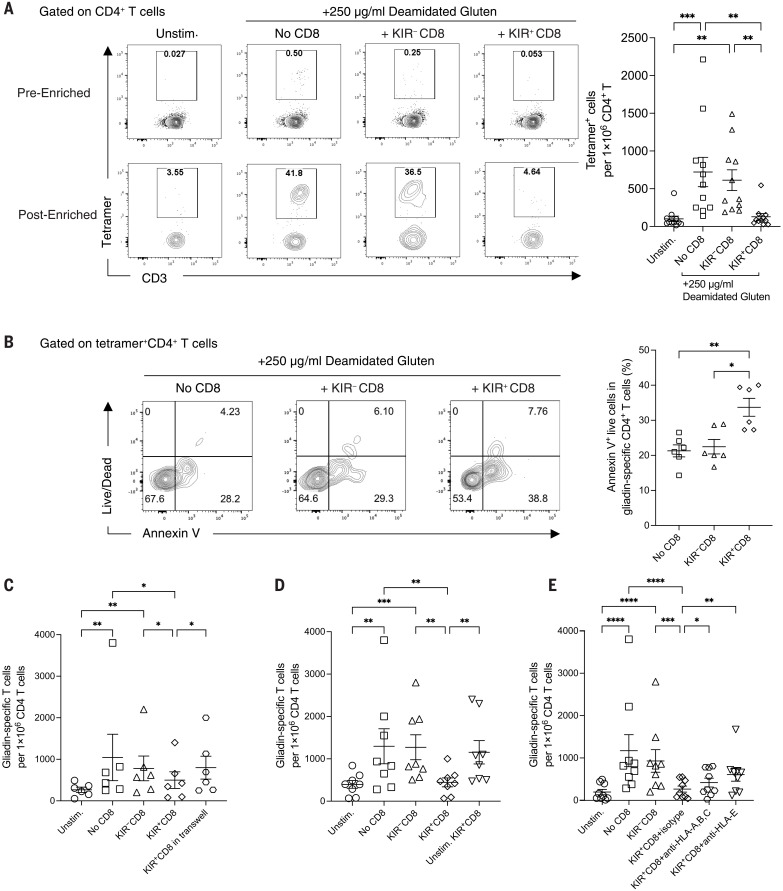
Elimination of gliadin-specific CD4^+^ T cells by KIR^+^CD8^+^ T cells. (**A**) Representative contour plots showing tetramer-bound CD4^+^ T cells before and after enrichment and summary of the number of gliadin-specific CD4^+^ T cells per 1 × 10^6^ CD4^+^ T cells on day 6. Experiments were repeated using PBMCs from 11 CeD patients. **P < 0.01; ***P < 0.001; Friedman test followed by multiple comparisons test. Unstim., unstimulated. (**B**) Representative contour plots and a summary graph displaying annexin V binding of gliadin-specific CD4^+^ T cells from the culture harvested on day 3 (*N* = 6). *P < 0.05; **P < 0.01; Friedman test followed by multiple comparisons test. (**C**) Frequency of gliadin-specific CD4^+^ T cells from the cell cultures in the presence or absence of KIR^–^ or KIR^+^CD8^+^ T cells or with KIR^+^CD8^+^ T cells separated by a 4-μm insert in a transwell plate (*N* = 6). *P < 0.05; **P < 0.01; Friedman test followed by multiple comparisons test. (**D**) Frequency of gliadin-specific CD4^+^ T cells from the PBMC cultures in the presence or absence of KIR^–^ or KIR^+^CD8^+^ T cells with or without preactivation (*N* = 8). **P < 0.01; ***P < 0.001; Friedman test followed by multiple comparisons test. (**E**) Frequency of gliadin-specific CD4^+^ T cells from the PBMC cultures in the presence or absence of preactivated KIR^–^ or KIR^+^CD8^+^ T cells with isotype control, anti–HLA-ABC, or anti–HLA-E blockade (*N* = 9). *P < 0.05; **P < 0.01; ***P < 0.001; ****P < 0.0001; Friedman test followed by multiple comparisons test.

We next investigated whether KIR^+^CD8^+^ T cells are the functional counterpart of mouse Ly49^+^ regulatory CD8^+^ T cells. Previously we had found that Ly49^+^CD8^+^ T cells suppress myelin oligodendrocyte glycoprotein (MOG)–specific pathogenic CD4^+^ T cells in a perforin-dependent manner (*4*, *7*). Deamidated gliadin derived from dietary gluten is the antigen for CD4^+^ T cells that drives autoimmune enteropathy in human CeD (*11*, *12*). Therefore, we explored whether KIR^+^CD8^+^ T cells can suppress gliadin-specific CD4^+^ T cells from CeD patients. CD8^–^ peripheral blood mononuclear cells (PBMCs) from human leukocyte antigen (HLA)–DQ2.5^+^ CeD patients were cultured with preactivated KIR^+^ and KIR^–^CD8^+^ T cells supplemented with deamidated gluten (fig. S2A). In the absence of KIR^+^CD8^+^ T cells, deamidated gluten profoundly stimulated the expansion of gliadin-specific CD4^+^ T cells. Notably, stimulated KIR^+^CD8^+^ T cells, but not KIR^–^CD8^+^ T cells or KIR^+^ NK cells, significantly reduced the number of gliadin-specific CD4^+^ T cells (Fig. 2A and fig. S2C) without affecting the number of total CD4^+^ T cells (fig. S2B). KIR^+^CD8^+^ T cells appeared to target only the pathogenic CD4^+^ T cells because they had no discernible effect on hemagglutinin (HA)–specific CD4^+^ T cells induced by influenza A HA peptides (fig. S2D) or on the proliferation of CD4^+^ T cells after anti-CD3 stimulation (fig. S2E). The suppression by KIR^+^CD8^+^ T cells was contact dependent because their inhibitory effects on gliadin-specific CD4^+^ T cells were abrogated when they were separated from the CD8^–^ PBMCs by a membrane insert (Fig. 2C). We also found increased annexin V binding on gliadin-specific CD4^+^ T cells in the presence of KIR^+^CD8^+^ T cells (Fig. 2B), indicating that these cells can induce apoptosis of the pathogenic CD4^+^ T cells. In the presence of high-dose interleukin-2 (IL-2), KIR^+^CD8^+^ T cells were still able to reduce the number of gliadin-specific CD4^+^ T cells (fig. S2F). Thus, KIR^+^CD8^+^ T cells suppress pathogenic CD4^+^ T cells through direct killing instead of a competition for IL-2, consistent with the perforin dependance of Ly49^+^CD8^+^ T cells in mice (*4*, *7*).

Previous studies have suggested that the regulatory function of mouse Ly49^+^CD8^+^ T cells is mediated by recognition of both classical (*7*) and nonclassical class I MHCs (*4*, *5*, *13*) on their target cells. Preactivated KIR^+^CD8^+^ T cells showed more-potent suppression of gliadin-specific CD4^+^ T cells than the untreated KIR^+^CD8^+^ T cells (Fig. 2D), which indicates that T cell receptor (TCR) activation is required to fully elicit their suppressive functions. Moreover, anti–HLA-ABC and anti–HLA-E blockade could partially reverse the suppression by KIR^+^CD8^+^ T cells (Fig. 2E). Thus, KIR^+^CD8^+^ T cells specifically eliminate gliadin-specific CD4^+^ T cells from the leukocytes of CeD patients through the recognition of classical and/or nonclassical class I MHC molecules.

To further investigate whether KIR^+^CD8^+^ T cells are the phenotypic equivalent of mouse Ly49^+^ T cells in humans, we performed RNA sequencing (RNA-seq) analysis on KIR^+^ versus KIR^–^CD8^+^ T cells from patients with MS to compare with mouse Ly49^+^CD8^+^ T cells in EAE, a mouse model of human MS. There were 778 differentially expressed genes (DEGs) between KIR^+^ and KIR^–^CD8^+^ T cells (table S1). Notably, KIR^+^CD8^+^ T cells showed a marked up-regulation of cytotoxic molecules, NK cell–associated genes, and cell-trafficking molecules, in addition to inhibitory KIR receptor genes (fig. S3A). Furthermore, KIR^+^CD8^+^ T cells expressed higher transcript levels for Helios (encoded by *IKZF2*), a transcription factor associated with regulatory functions of both CD4^+^ and CD8^+^ T cells (*6*). KIR^+^CD8^+^ T cells also down-regulated naïve and memory T cell–associated molecules and the costimulatory receptor *CD28* (fig. S3A), which is one of the key features for regulatory CD8^+^ T cell populations in mice and humans (*14*). Gene ontology enrichment analysis of these DEGs showed enrichment for T cell activation, proliferation, migration, and differentiation (fig. S3B). Moreover, gene set enrichment analysis (GSEA) (*15*, *16*) revealed that approximately half of the top 200 genes up-regulated in Ly49^+^CD8^+^ T cells were also higher in KIR^+^CD8^+^ T cells (fig. S3C). Previously, we found that Ly49^+^CD8^+^ T cells expressed 16 of the 60 genes conserved in CD4^+^ T_regs_ (*7*). These same T_reg_ signature genes (*17*) were also enriched in KIR^+^CD8^+^ T cells (fig. S3D). Thus, human KIR^+^CD8^+^ T cells share many similarities with Ly49^+^CD8^+^ T cells from EAE mice.

RNA-seq analysis of KIR^+^ versus KIR^–^CD8^+^ T cells from healthy subjects and patients with different autoimmune diseases (including MS, SLE, and CeD) identified a set of 963 DEGs. Many of them overlapped with the DEGs previously defined in MS (fig. S3E and table S2), but larger-fold changes were observed in patients with higher frequencies of KIR^+^CD8^+^ T cells (fig. S4A). Consistent with the transcriptional profiles, KIR^+^CD8^+^ T cells had higher protein expression of granzyme B, perforin, CX3CR1, KLRG1, CD244, TIGIT, T-bet, and Helios and lower levels of CCR7, CD27, and CD28 (fig. S4B). Similar to the circulating KIR^+^CD8^+^ T cells, both kidney and synovial KIR^+^CD8^+^ T cells up-regulated *KLRG1*, *CD244*, *TIGIT*, *CX3CR1*, *PRF1*, *GZMB*, and *IKZF2* and down-regulated *CD28* and *CCR7* (fig. S5). Thus, KIR^+^CD8^+^ T cells appear to be the functional and phenotypic equivalent of mouse Ly49^+^CD8^+^ T cells in humans, with many conserved features in both healthy subjects and those with autoimmune diseases.

Because the KIR^+^CD8^+^ T cells we focused on in this study express inhibitory KIR receptors, which contain intracellular ITIMs, we investigated how these KIR receptors contribute to the differentiation and functionality of KIR^+^CD8^+^ T cells. CD8^+^ T cells with low KIR3DL1 or KIR2DL3 expression displayed higher surface CX3CR1 and intracellular granzyme B and perforin compared with those with high KIR expression (fig. S6, A to B). RNA-seq followed by gene ontology enrichment analysis confirmed that CD8^+^ T cells with low KIR expression displayed enhanced cytotoxicity and T cell activation (fig. S6, C to D). Thus, the high expression of inhibitory KIR receptors suppresses the activation and effector functions of the KIR^+^CD8^+^ T cells. This may allow for the precise control of their activity to avoid bystander suppression.

## Increased KIR^+^CD8^+^ T cells in SARS-CoV-2– and influenza-infected patients

**Fig. 3.**
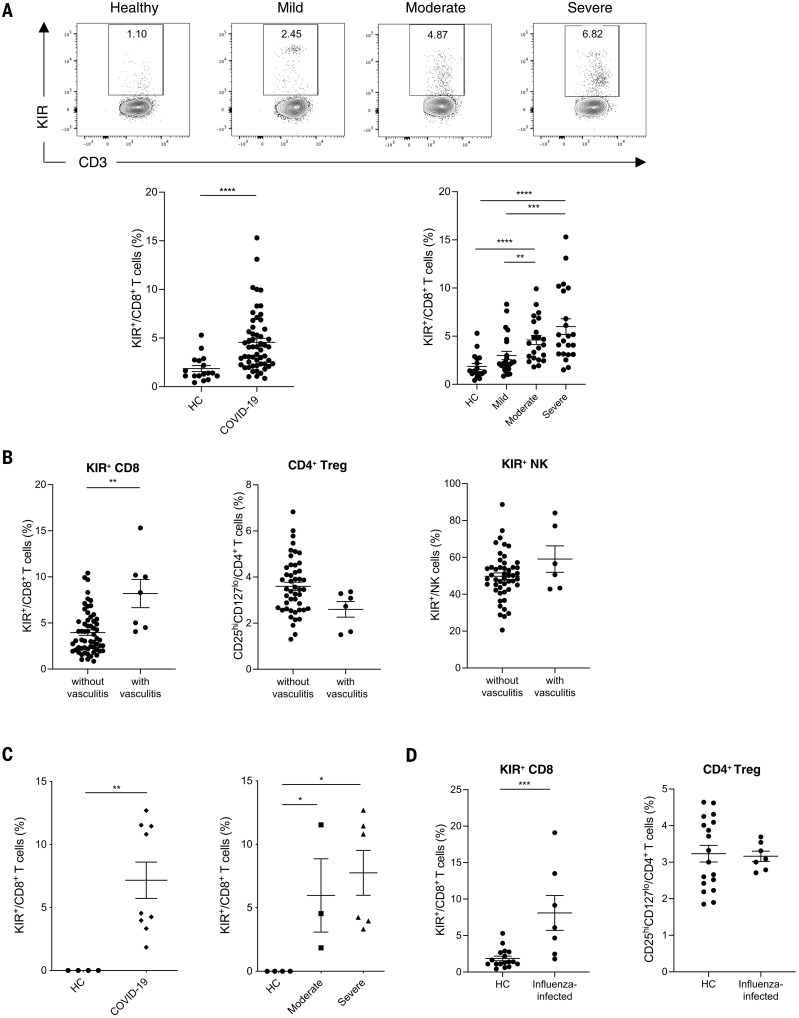
Increased KIR^+^CD8^+^ T cells in infectious diseases. (**A**) Representative contour plots and summary scatter plots showing the percentage of KIR^+^ cells in CD8^+^ T cells from the blood of 17 HCs and 53 COVID-19 patients with varying disease severity (mild, *N* = 23; moderate, *N* = 17; severe, *N* = 13). ****P < 0.0001; Mann-Whitney test (left). **P < 0.01; ***P < 0.001; ****P < 0.0001; Kruskal-Wallis test followed by multiple comparisons test (right). (**B**) Frequency of KIR^+^CD8^+^ T cells, CD4^+^ T_regs_ (CD25^hi^CD127^lo^), and KIR^+^ NK cells in the blood of COVID-19 patients with or without vasculitis. **P < 0.01; Mann-Whitney test. (**C**) Frequency of CD8^+^ T cells expressing *KIR* transcripts (*KIR3DL1*, *KIR3DL2*, *KIR2DL3*, or *KIR2DL1*) in the bronchoalveolar lavage fluid of HCs (*N* = 4) versus COVID-19 patients (*N* = 9) (left; **P < 0.01; Mann-Whitney test) and HCs versus COVID-19 patients with moderate (*N* = 3) or severe (*N* = 6) disease (right; *P < 0.05; Kruskal-Wallis test followed by multiple comparisons test). (**D**) Frequency of KIR^+^CD8^+^ T cells and CD4^+^ T_regs_ (CD25^hi^CD127^low^) in the blood of HCs (*N* = 17) versus influenza-infected patients (*N* = 7). ***P < 0.01; Mann-Whitney test.

Although it was previously accepted that most self-specific T cells were eliminated in the thymus, recent work has shown that many such cells survive and populate the periphery of both humans and mice (*18*, *19*). We have hypothesized that this occurs because the threat of infectious diseases (*20*) necessitates a complete T cell repertoire (*18*, *21*), such that even self-reactive T cells might be needed in the response to a particular pathogen. Consistent with this are classic experiments showing that infectious diseases or treatments that mimic them can activate self-specific T cells (*22*). This led us to hypothesize that KIR^+^CD8^+^ T cells might be elevated during an infection to control autoreactive T cells. We first analyzed 53 patients with COVID-19. We found that KIR^+^CD8^+^ T cells were substantially elevated in many of these patients and higher levels correlated with more severe disease (Fig. 3A). Moreover, the highest frequencies of KIR^+^CD8^+^ T cells were found in patients with vasculitis or embolism and, to a lesser extent, in those with acute respiratory distress syndrome (ARDS) (Fig. 3B and fig. S7, C to D), which are common complications of this disease, likely caused by excessive inflammation. Thus, increased levels of KIR^+^CD8^+^ T cells may be prognostic for autoimmune-related pathologies during a severe acute respiratory syndrome coronavirus 2 (SARS-CoV-2) infection. By contrast, we did not observe any difference in the levels of CD25^hi^CD127^lo^CD4^+^ T_regs_ or KIR^+^ NK cells in COVID-19 patients compared with HCs or in COVID-19 patients with different disease severities or complications (fig. S7, A to D, and Fig. 3B). Using publicly available single-cell RNA sequencing (scRNA-seq) data (*23*), we also found an increased frequency of KIR^+^CD8^+^ T cells in the bronchoalveolar lavage fluid of COVID-19 patients with moderate or severe disease compared with that from HCs (Fig. 3C). Additionally, an increased frequency of KIR^+^CD8^+^ T cells, but not CD4^+^ T_regs_, was observed in the peripheral blood of influenza-infected patients (Fig. 3D), which suggests that KIR^+^CD8^+^ T cells are generally induced as part of an infectious disease response.

## Commonality and heterogeneity of KIR^+^CD8^+^ T cells

**Fig. 4.**
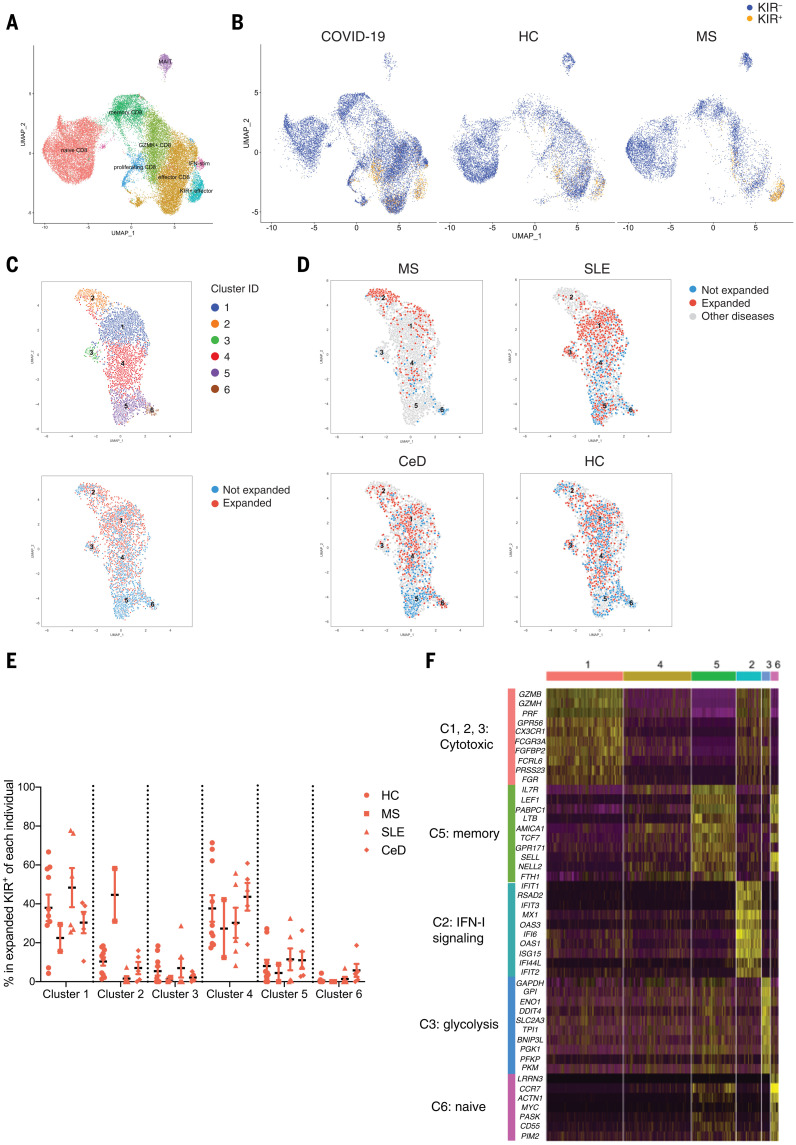
scRNA-seq analysis of KIR^+^CD8^+^ T cells in the blood. (**A** and **B**) scRNA-seq analysis of total CD8^+^ T cells from the blood of HCs (*N* = 10), MS patients (*N* = 6), and COVID-19 patients (*N* = 25) by 10X Genomics. (A) UMAP plot of the eight subpopulations identified by unsupervised clustering. (B) UMAP plots showing the distribution of KIR^+^CD8^+^ T cells (expressing *KIR3DL1*, *KIR3DL2*, *KIR2DL1*, or *KIR2DL3* transcripts) and KIR^–^CD8^+^ T cells from HCs, MS patients, and COVID-19 patients. (**C** to **F**) KIR^+^CD8^+^ T cells in the blood of HCs (*N* = 10) and patients with MS (*N* = 2), SLE (*N* = 6), and CeD (*N* = 5) were sorted for scRNA-seq using the Smart-seq2 protocol and analyzed using the R package “Seurat.” (C) UMAP plots showing KIR^+^CD8^+^ T cells segregated into six clusters (top) and the distribution of expanded (≥2 cells expressing same TCR) and unexpanded (cells expressing unique TCRs) cells (bottom). (D) UMAP plots of KIR^+^CD8^+^ T cells from MS, SLE, and CeD patients and HCs are shown, with expanded and unexpanded cells annotated with different colors (expanded, red; unexpanded, blue; other diseases, gray). (E) Cluster compositions of expanded KIR^+^CD8^+^ T cells from each individual. (F) Heatmap showing expression of the top 10 genes differentially expressed in each cluster, with the categories of each group of genes annotated on the left.

We next performed scRNA-seq analysis on peripheral blood CD8^+^ T cells from HCs, MS patients, and COVID-19 patients (*24*) using the 10X Genomics platform (*25*). KIR^+^CD8^+^ T cells from different conditions formed a distinct cluster with high expression of effector genes as well as *KIR* transcripts (Fig. 4, A to B, and table S3). Compared with KIR^–^ effector CD8^+^ T cells, KIR^+^ effector CD8^+^ T cells expressed higher levels of *IKZF2* and NK cell–associated genes (e.g., *TYROBP*, *KLRC2*, *KLRC3*, *NCR1*, and *NCR3*) while expressing lower levels of *IL7R*, *CD27*, and *KLRB1* (table S3). Thus, these findings reveal the commonality of KIR^+^CD8^+^ T cells across physiological and diseased statuses as well as their specialness relative to other CD8^+^ T cells.

To better understand the similarity and heterogeneity of KIR^+^CD8^+^ T cells under different circumstances, we performed scRNA-seq on 4512 KIR^+^CD8^+^ T cells sorted from healthy subjects and patients with MS, SLE, or CeD using the Smart-seq2 protocol (*26*) and analyzed their TCR α and β sequences (*27*). Unsupervised clustering of these KIR^+^CD8^+^ T cells identified six clusters, with clusters 1 to 3 mostly containing expanded KIR^+^CD8^+^ T cells (≥2 cells expressing same TCR), and clusters 5 and 6 consisting of unexpanded cells expressing unique TCRs (Fig. 4C and fig. S8, A to B). Expanded KIR^+^ cells in clusters 1 to 3 had higher transcripts for cytotoxic molecules (e.g., *GZMH*, *GZMB*, and *PRF1*) and genes associated with effector T cells (e.g., *FCGR3A*, *FGFBP2*, and *CX3CR1*). Cluster 2, which was more restricted to expanded KIR^+^ cells from MS patients, showed higher levels of type I interferon (IFN)–responding genes. Cluster 3—specific to expanded KIR^+^ cells from a subset of HCs and SLE patients—showed higher expression of genes involved in glycolysis (Fig. 4, D to F, and table S4). Cells in cluster 4 were in a transitional state, with a loss of memory-associated features. Clusters 5 and 6 displayed memory and naïve signatures, respectively (Fig. 4F and table S4), and accounted for a small proportion of total KIR^+^CD8^+^ T cells (fig. S8A). T cell clones expressing identical TCRs could be found in different clusters, indicating possible lineage relationships. Additionally, clonally expanded KIR^+^CD8^+^ T cells in COVID-19 patients identified from the previous 10X Genomics scRNA-seq (*24*) displayed a higher expression of cytotoxic genes while downregulating naïve- or memory-associated genes compared with unexpanded KIR^+^CD8^+^ T cells (fig. S8, C to D). In the assay with celiac PBMCs, CCR7^–^ effector KIR^+^CD8^+^ T cells displayed stronger suppressive activity against gliadin-specific CD4^+^ T cells than CCR7^+^KIR^+^CD8^+^ T cells (fig. S2G), which was consistent with their up-regulated cytotoxic functions as revealed by the scRNA-seq. Thus, in parallel with clonal expansion, KIR^+^CD8^+^ T cells may lose their naïve or memory attributes, enter the differentiation program for effector T cells, and then suppress pathogenic CD4^+^ T cells through cytotoxicity. There are common features shared by KIR^+^CD8^+^ T cells from healthy subjects and from subjects with different diseases, yet there is also heterogeneity associated with different diseases or treatments.

**Fig. 5.**
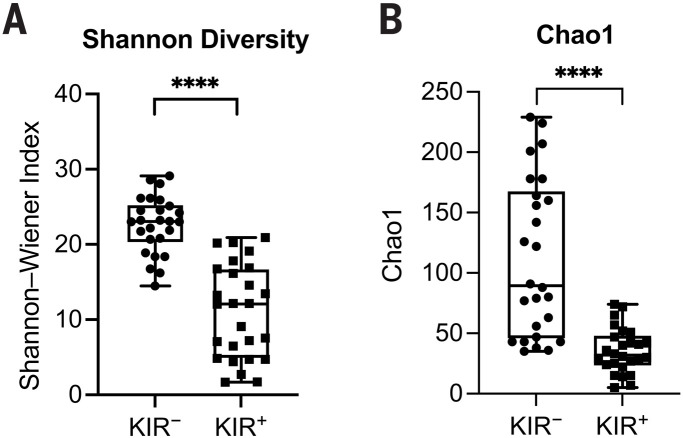
Analysis of TCR sequences of KIR^+^CD8^+^ T cells. (**A** and **B**) Summary histograms showing Shannon-Wiener indices (A) and Chao estimates (B) of TCRs of KIR^–^ versus KIR^+^CD8^+^ T cells from 26 subjects, including 11 healthy donors, two MS, five SLE, three CeD, and five T1D patients as evaluated by VDJtools. ****P < 0.0001; Wilcoxon matched-pairs signed-rank test.

We next compared the diversity of KIR^+^CD8^+^ TCRs with KIR^–^CD8^+^ TCRs from the same individuals (*N* = 26) and found that TCRs of KIR^+^CD8^+^ T cells had significantly lower Shannon-Wiener indices and Chao estimates than KIR^–^CD8^+^ T cells (Fig. 5, A to B). Thus, the TCR repertoire of KIR^+^CD8^+^ T cells is less diverse, consistent with a previous study that KIR^+^CD8^+^ T cells display a more restricted TCR Vβ chain usage (*9*). To compare the antigen specificities of KIR^+^CD8^+^ T cells from different disease types, we also analyzed the TCR sequences using GLIPH2 (*28*), which is an algorithm to cluster TCRs that recognize the same antigen in most cases. TCRs of KIR^+^CD8^+^ T cells from healthy donors and different diseases could be grouped into the same GLIPH clusters, although with different extents of clonal expansion (Table 1), which indicates that they may recognize the same antigens that commonly exist under physiological and different pathological conditions. Thus, expanded KIR^+^CD8^+^ T cells have shared phenotypes and antigen specificity independent of disease type. Although the analysis above shows that the TCR repertoire of KIR^+^CD8^+^ T cells is less diverse than KIR^–^CD8^+^ T cells generally, it is still considerably diverse, using multiple classical HLAs and HLA-E and probably recognizing many antigenic peptides as well.

**Table 1.**
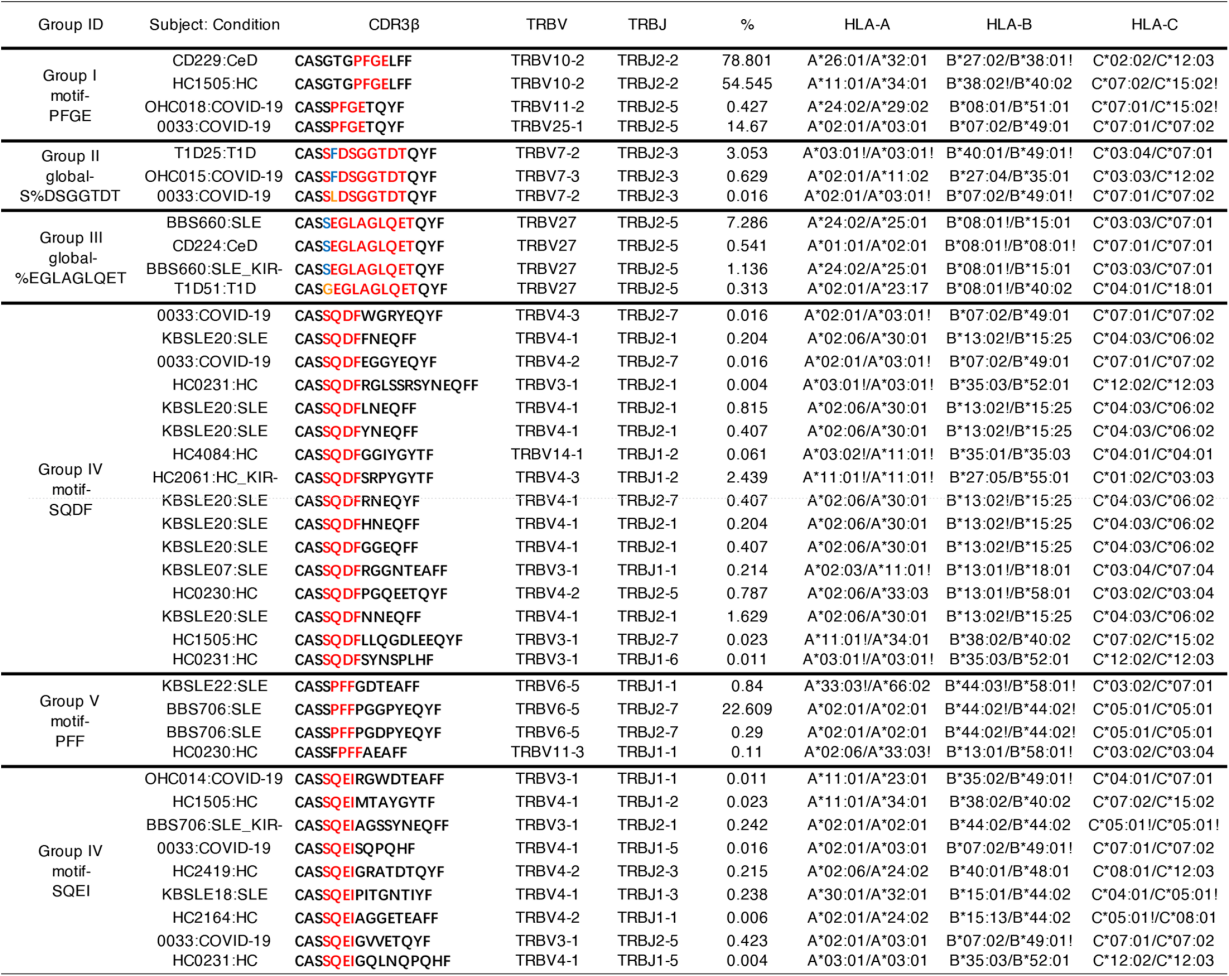
Selected specificity groups from the GLIPH2 analysis that contained TCRβ sequences from three or more individuals and exhibited significant bias of V-gene usage (*P* < 0.05). Significant motif residues are highlighted in red in CDR3 alignments.

## Regulatory CD8^+^ T cells suppress autoimmunity developed after virus infection

**Fig. 6.**
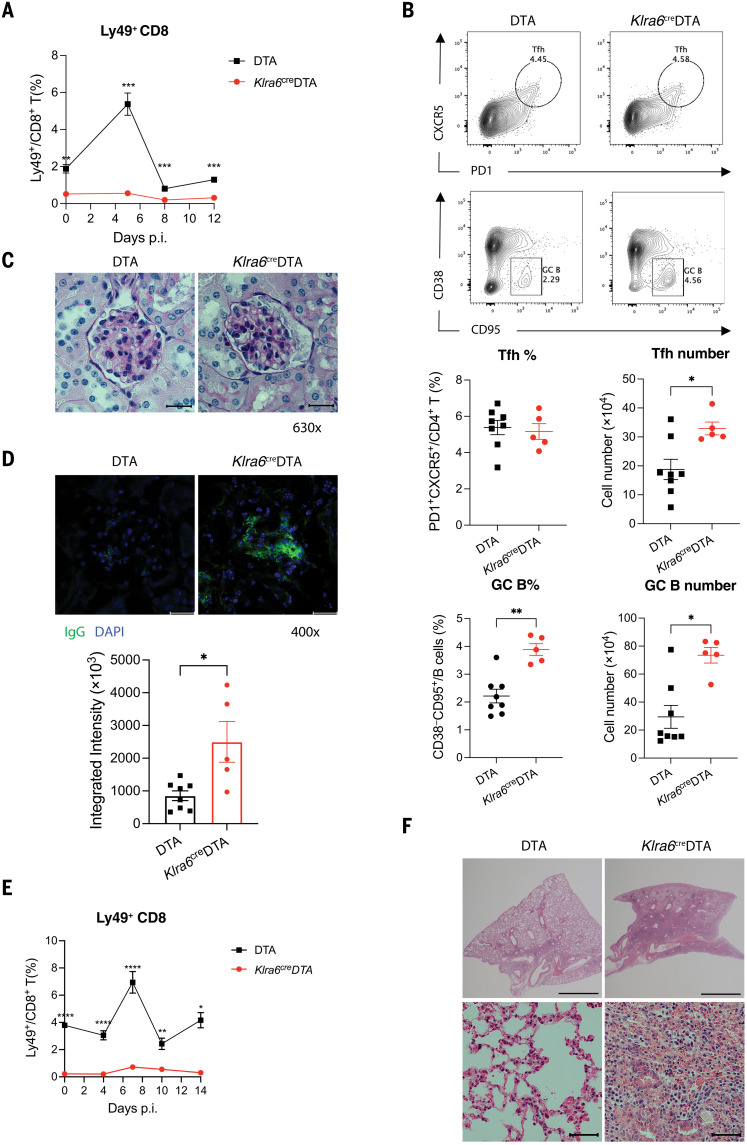
Exacerbated autoimmunity in *Klra6*^cre^DTA mice after viral infection. (**A**) Frequency of Ly49^+^CD8^+^ T cells in the blood of DTA mice (*N* = 8) and *Klra6*^cre^DTA mice (*N* = 5) 0, 5, 8, and 12 days after LCMV-Armstrong infection. **P < 0.01; ***P < 0.001; repeated measures (RM) two-way analysis of variance (ANOVA) followed by multiple comparisons test. p.i., postinfection. (**B**) Representative contour plots and summarized scatter plots displaying frequency and absolute number of PD1^+^CXCR5^+^CD4^+^ T cells (T_FH_) and CD38^–^CD95^+^ GC B cells in the spleen of DTA mice (*N* = 8) and *Klra6*^cre^DTA mice (*N* = 5) 30 days after LCMV-Armstrong infection. *P < 0.05; **P < 0.01; Mann-Whitney test. Tfh, T follicular helper cell. (**C**) Representative kidney pathology of day-30 LCMV-infected DTA mice and *Klra6*^cre^DTA mice assessed by PAS staining (630X; scale bars, 20 μm). (**D**) IgG deposition in glomeruli of the kidneys of DTA mice (*N* = 8) and *Klra6*^cre^DTA mice (*N* = 5) 30 days after LCMV infection accessed by immunofluorescence staining (400X; scale bars, 20 μm) and quantified by ImageJ. *P < 0.05; Mann-Whitney test. DAPI, 4′,6-diamidino-2-phenylindole. (**E**) Frequency of Ly49^+^CD8^+^ T cells in the blood of DTA mice (*N* = 6) and *Klra6*^cre^DTA mice (*N* = 5) after influenza A-PR8 infection. *P < 0.05; **P < 0.01; ****P < 0.0001; RM two-way ANOVA followed by multiple comparisons test. (**F**) Microscopy of representative H&E staining of lung sections from DTA mice and *Klra6*^cre^DTA mice 60 days after influenza infection (top, 20X; scale bars, 2 mm) (bottom, 630X; scale bars, 50 μm). Representative data from two independent experiments are shown. The means ± SEMs are indicated.

We next studied the effect of selective ablation of Ly49^+^CD8^+^ T cells on virus-infected mice. Because Ly49F (encoded by *Klra6*) is expressed on 90% of Ly49^+^CD8^+^ T cells but only a small fraction of NK cells (*5*, *29*), we generated a *Klra6*^cre^ mouse line. In *Klra6*^cre^R26R-EYFP mice (EYFP, enhanced yellow fluorescent protein), all of the YFP^+^ cells expressed CD3 or NK1.1, indicating that the Cre expression is restricted to NK, T, and NKT cells (fig. S9, A to B). In *Klra6*^cre^DTA mice, there was a 50 to 75% decrease of Ly49^+^CD8^+^ T cells in the spleen and lymph nodes, whereas Ly49^+^ NK cells did not show a significant reduction (fig. S9C), consistent with the preferential expression of Ly49F on CD8^+^ T cells. *Klra6*^cre^DTA mice did not appear to spontaneously develop any autoimmune disorders or exhibit changes in their frequencies of effector T cells, T follicular helper (T_FH_) cells, or germinal center (GC) B cells up to 8 months of age (fig. S9, D to E). When mice were infected with lymphocytic choriomeningitis virus (LCMV)–Armstrong or influenza A-PR8 viruses, there was a surge of Ly49^+^CD8^+^ T cells in the blood of wild-type mice (Fig. 6, A and E), consistent with our previous observations of increased KIR^+^CD8^+^ T cells in patients with acute SARS-CoV-2 or influenza infection. However, the frequency of Ly49^+^CD8^+^ T cells in *Klra6*^cre^DTA mice remained very low at all times after viral challenge, whereas Ly49^+^ NK cells in *Klra6*^cre^DTA mice showed only minor reductions compared with DTA (wild-type) mice (figs. S10A and S11A), indicating a selective and efficient ablation of Ly49^+^CD8^+^ T cells.

With either LCMV or influenza infection, *Klra6*^cre^DTA mice showed no difference in viral clearance (figs. S10B and S11B) or levels of effector- and virus-specific CD4^+^ and CD8^+^ T cell responses (figs. S10C and S11C) compared with those of controls. However, *Klra6*^cre^DTA mice, but not DTA mice, developed autoimmune pathology characterized by increased numbers of T_FH_ and GC B cells in the spleen (Fig. 6B) as well as glomerular nephritis (Fig. 6C) and immunoglobulin G (IgG) deposition (Fig. 6D) in the kidney 30 days after LCMV-Armstrong infection. Similarly, influenza-infected *Klra6*^cre^DTA mice displayed more-severe inflammation and pathology characterized by peribronchial and interstitial accumulation of inflammatory cells, pulmonary consolidation, and fibrous tissue hyperplasia in the lung 60 days after influenza infection (Fig. 6E). Thus, this population of regulatory CD8^+^ T cells appears to suppress autoimmunity that can develop after viral infection rather than having any discernable role in viral clearance.

## Discussion

Here, we identify KIR^+^CD8^+^ T cells as an important regulatory CD8^+^ T cell subset in humans. Previous studies have shown that KIR^+^CD8^+^ T cells are terminally differentiated cells and display a restricted TCR repertoire (*9*, *30*), which is consistent with our findings. Correlations between KIR^+^CD8^+^ T cells and tumor immune surveillance (*31*, *32*) or chronic viral infections (*33*, *34*) have been reported, but the suppressive functions of this population have not been clearly defined previously. We demonstrate the regulatory function of KIR^+^CD8^+^ T cells toward pathogenic CD4^+^ T cells through an in vitro functional assay using PBMCs from CeD patients. This effect of KIR^+^CD8^+^ T cells seems specific to self-reactive or otherwise pathogenic T cells, but not CD4^+^ T cells recognizing foreign antigens. Similar to the perforin- or Fas-FasL–dependent suppression of self-reactive CD4^+^ T cells by murine Ly49^+^CD8^+^ T cells (*4*, *7*, *35*), human KIR^+^CD8^+^ T cells likely target pathogenic CD4^+^ T cells through direct killing because KIR^+^CD8^+^ T cells significantly up-regulate cytotoxic molecules and suppress gliadin-specific CD4^+^ T cells in a contact-dependent manner by inducing apoptosis. Additionally, the destruction of pathogenic CD4^+^ T cells by KIR^+^CD8^+^ T cells appears to depend on recognition of both classical and nonclassical class I MHC molecules because the blockade of either HLA-ABC or HLA-E can reverse the suppression by KIR^+^CD8^+^ T cells. However, the MHC restrictions of the KIR^+^ regulatory CD8^+^ T cells at work vary between individuals. Because KIR receptors deliver inhibitory signals through their ITIMs to suppress the activation and functions of KIR^+^CD8^+^ T cells, antibody-dependent blockade of KIR3DL1 or KIR2DL3 may further enhance the suppressive activity of KIR^+^CD8^+^ T cells toward pathogenic CD4^+^ T cells.

We frequently observed an increased frequency of KIR^+^CD8^+^ T cells in the blood as well as in the inflamed tissues of patients with autoimmune disease. This increase positively correlated with disease activity in CeD intestinal biopsies. The expansion of KIR^+^CD8^+^ T cells in the context of autoimmune diseases may act as a negative feedback mechanism to ameliorate pathogenesis by killing autoreactive T cells. Moreover, increased KIR^+^CD8^+^ T cells were found in SARS-CoV-2– or influenza-infected patients and were associated with autoimmune-related complications in COVID-19 patients. This suggests that increase of KIR^+^CD8^+^ T cells is a general mechanism induced during an infection, which, in classical murine studies, has been seen to break tolerance—that is, allow the activation of self-reactive T cells that normally require innate immune signals in addition to their cognate antigens (*36*). In mice, there was also a surge of circulating Ly49^+^CD8^+^ T cells after LCMV-Armstrong or influenza A-PR8 infection. Selective ablation of Ly49^+^CD8^+^ T cells did not interfere with antiviral immune responses but led to exacerbated autoimmunity after virus infection. This is in line with the autoimmune phenotypes secondary to LCMV infection in *Helios^–/–^* mice, in which both CD8^+^ and CD4^+^ regulatory T cells are defective (*6*). Therefore, we hypothesize that a major role of these CD8^+^ regulatory T cells is to control autoreactive T cells that are activated during an infection, likely because they are cross-reactive to foreign antigens. This would allow an organism to have the benefit of a complete T cell repertoire while limiting damage from the autoreactive clones. This type of peripheral tolerance is distinct from and likely complementary to the one mediated by CD4^+^ T_regs_, which represent a separate lineage of T cells and do not appear to be generally active in the human infectious diseases analyzed here or in murine infections (*37*, *38*).

Although there is still much more to learn about KIR^+^CD8^+^ T cells and their murine equivalents, the data presented here and in previous studies indicate that they represent an important element in peripheral tolerance and in our understanding of the relationship between autoimmunity and infectious diseases. Further characterization of this pathway and how it may break down in autoimmune diseases and severe infections, like COVID-19, will be important challenges for the future. Likewise, our findings on the KIR^+^CD8^+^ T cells and their properties described here may be useful in understanding key cellular dynamics in immune dysregulation and in potential therapeutic approaches to suppress undesirable self-reactivity in autoimmune or infectious diseases.

## Materials and methods

### Human samples

Our study cohort of patients with autoimmune disorders met classification criteria for SLE (*39*), CeD (*40*), or MS (*41*), respectively. Collection of blood or biopsies from patients with SLE, CeD, or MS was covered by IRB-14734, IRB-20362, and IRB-36061. Blood samples from patients during influenza virus infection were obtained from patients who had influenza-like symptoms and were tested positive for influenza A virus at the Emergency Department or the Express Outpatient Clinic at Stanford Hospital, which is covered by IRB-22442. Blood from healthy subjects was requested from the Stanford Blood Center or drawn from healthy volunteers under IRB-40146. The protocols listed above have been approved by the Research Compliance Office of Stanford University. PBMCs from MS patients were also obtained from the Multiple Sclerosis Center at the University of California, San Francisco (UCSF), with the protocol approved by the committee on Human Research at UCSF. Informed written consent was obtained from all participants. Detailed information of the HCs and patients with autoimmune diseases included in the study is provided as table S5. PBMCs were isolated from the blood through density gradient centrifugation (Ficoll-Paque, GE Healthcare). Duodenal biopsies from CeD patients were treated twice with 6 mM EDTA in calcium/magnesium-free Hanks’ balanced salt solution (HBSS) for 30 min at 37°C. Supernatants containing the epithelial fractions were combined, washed, and kept on ice until staining. The remaining tissues were minced and incubated with 200 μg/ml of Liberase TL and 20 U/ml of deoxyribonuclease (DNase) I in Iscove’s modified Dulbecco’s medium (IMDM) for 30 min at 37°C. Digested cell suspensions were passed through a 100-μm cell strainer, washed with complete media, and combined with the epithelial fraction for staining.

For sample collection of COVID-19 patients, enrollment included any adult with reverse transcription polymerase chain reaction (RT-PCR)–positive COVID-19. Informed consent was obtained from each patient or from the patient’s legally authorized representative if the patient was unable to provide consent. Participants were excluded if they were taking any experimental medications (i.e., those medications not approved by a regulatory agency for use in COVID-19). COVID-19 severity of illness was defined as described in the literature (*42*). Collection of blood from COVID-19 patients was covered by IRB-55689 and NCT04373148. Handling of COVID-19 PBMCs for flow cytometric analysis was covered under APB-3343-MD0620. The IRB and APB protocols mentioned above have been approved by the Research Compliance Office of Stanford University. Clinical metadata were obtained from the Stanford clinical data electronic medical record system as per consented participant permission, and definitions and diagnoses of disease were used according to Harrison’s Principles of Internal Medicine, 20e. Clinical metadata for the COVID-19 patients in this study are presented in table S6.

### Mice

R26R-EYFP mice (stock no. 006148) and ROSA-DTA mice (stock no. 009669) were obtained from the Jackson Laboratory. *Klra6*^cre^ mice were generated by the Stanford Transgenic, Knockout and Tumor model Center. *Klra6* reporter mice were generated by crossing *Klra6*^cre^ mice to R26R-EYFP mice. *Klra6*^cre^DTA mice were generated by crossing *Klra6*^cre^ mice to ROSA-DTA mice. ROSA-DTA (wild-type) littermates were used as controls in the experiments described here. All mice were housed in the specific pathogen-free animal facilities at Stanford University. Experiments in this study were covered by the following animal protocols approved by the Animal Care and Use Committee of Stanford University: APLAC-10081, APLAC-34021, and APLAC-32763.

### Flow cytometric analysis

The fluorescent dye–conjugated antibodies used for staining were listed in table S7. Frozen cell samples were thawed and washed in 10% fetal bovine serum (FBS) with benzonase (Sigma-Aldrich, 25 U/ml) in RPMI. After 450*g* centrifugation, cells were treated with Fc receptor (FcR) block (Biolegend, 10 μg/ml) in FACS buffer [0.5% bovine serum albumin (BSA), 2 mM EDTA in phosphate-buffered saline (PBS)] for 10 min followed by staining with antibodies against surface molecules (30 min, 4°C). For intracellular staining, cells were fixed and permeabilized with the Intracellular Fixation & Permeabilization Buffer Set (eBioscience), followed by staining with antibodies against intracellular antigens (30 min, 4°C). Cells were acquired on an LSR II flow cytometer (BD), and data were analyzed using FlowJo X. Dead cells were excluded based on viability dye staining (LIVE/DEAD Fixable Near-IR Dead Cell Stain, ThermoFisher).

### Functional assay

Chymotryptic gluten digests were deamidated with recombinant human transglutaminase 2, as described previously (*43*). PBMCs were isolated from blood of HLA-DQ2.5^+^ CeD patients on day 0. CD8^+^ T cells were purified from PBMCs using CD8 microbeads (Miltenyi) per manufacturer’s instructions, stained with flow antibodies, and live CD3^+^CD56^–^CD8^+^KIR^+^ or KIR^–^ T cells were sorted out by FACSAria Fusion flow cytometer (BD). The sorted KIR^+^ and KIR^–^CD8^+^ T cells were stimulated with anti-CD3/CD28 beads (Gibco) at 1:1 ratio (1 μl of beads per 4 × 10^4^ cells) supplemented with 50 U/ml of IL-2 in 96-well plates for 18 hours. KIR^+^ and KIR^−^ NK cells were sorted for PBMCs and rested overnight. The CD8^–^ PBMCs were stimulated with 250 μg/ml of deamidated gluten or 10 μg/ml of influenza A HA 306-318 peptide (PKYVKQNTLKLAT) or left unstimulated at 3 × 10^5^ to 1 × 10^6^ cells/100 μl per well supplemented with 50 U/ml or 300 U/ml of IL-2. X-VIVO 15 with Gentamicin L-Gln (Lonza) supplemented with 10% human AB serum (Sigma-Aldrich) was used as culture medium. After 18 hours, anti-CD3/CD28 beads were removed from CD8^+^ T cells by a magnet and KIR^+^ or KIR^–^CD8^+^ T cells or NK cells were added to the culture of CD8^–^ PBMCs at a 1:30 ratio. In the class I MHC blockade experiments, 10 μg/ml of anti–HLA-ABC (W6/32, Biolegend), anti–HLA-E (3D12, eBioscience), or isotype controls were added to the culture. In the transwell assay, CD8^–^ PBMCs were cultured with 250 μg/ml of deamidated gluten and 50 U/ml of IL-2 in the lower chamber, and preactivated KIR^+^CD8^+^ T cells were added to the upper chamber of the Millicell-96 Cell Culture Insert Plate (Millipore Sigma, cat. no. PSHT004R1). On day 3, 50 U/ml of IL-2 was added to the cultures. Cells were harvested on day 6 and stained with 10 μg/ml of HLA-DQ2.5 tetramers (*44*) complexed with four disease-relevant and immunodominant gliadin T cell epitopes (DQ2.5-glia-α1a, QLQPFPQPELPY; DQ2.5-glia-α2, PQPELPYPQPE; DQ2.5-glia-ω1, QQPFPQPEQPFP; and DQ2.5-glia-ω2, FPQPEQPFPWQP) (*45*) or 10 μg/ml of HLA-DR4 tetramers complexed with influenza A HA 306-318 peptide for 45 min at room temperature. Magnetic bead enrichment of tetramer^+^ CD4^+^ T cells was performed as previously described (*46*). Cells were washed with FACS buffer and then stained with antibodies against surface molecules for 30 min at 4°C. After two washes with FACS buffer, 10% of the cells were reserved for FACS analysis and 90% were labeled with anti–phycoerythrin (PE) microbeads and subjected to magnetic bead enrichment of PE-conjugated tetramer-positive cells using a single magnetic activated cell sorting (MACS) column according to the manufacturer’s protocol (Miltenyi). Cells were also harvested on day 3 to measure annexin V binding (BD) on gliadin-specific CD4^+^ T cells. All cells were acquired on an LSR II flow cytometer (BD), gated on live CD3^+^CD4^+^CD8^–^TCRαβ^+^ cells, and analyzed using FlowJo X software. The frequency of tetramer-positive cells was calculated by dividing the number of postenrichment tetramer^+^ CD4^+^ T cells by the number of CD4^+^ T cells in the pre-enrichment sample multiplied by 9 (to account for the fact that 90% of the cells were used for the enrichment).

### Bulk RNA-seq gene-expression quantification and data analysis

Live KIR^+^ or KIR^−^ CD8^+^ T cells were bulk sorted directly into 500 μl of TRIzol (ThermoFisher, cat. no.15596026) by FACSAria Fusion flow cytometer (BD). Total RNA was extracted from TRIzol samples using chloroform separation and isopropanol precipitation and then RNAeasy Plus Mini kit (Qiagen) for clean-up. After analysis on the 2100 Bioanalyzer (Agilent), the sequencing libraries were prepared using the SMARTer Stranded RNA-seq Kit (Clontech). The resulting library was sequenced on the HiSeq 4000 platform (Illumina) in Stanford Functional Genomics Facility. For each sample in the bulk RNA-seq library, 75–base pair (bp) paired-end reads were acquired from the sequencer. We aligned the reads to the human reference genome (NCBI GRCh38) using STAR v2.7.0e (*47*). Gene counts were quantified and normalized (TPM) with Salmon (*48*). DEGs were determined using the DESeq function (adjusted *P* < 0.05, fold change > 2) in the DESeq2 R package (*49*). Heatmaps were generated with seaborn.clustermap in python. Gene Ontology analysis plots were generated with the R package “clusterProfiler.” To generate gene sets for GSEA, we selected the top 200 genes up-regulated in Ly49^+^CD8^+^ T cells compared with Ly49^–^CD8^+^ T cells in EAE mice (*7*) and the previously reported CD4^+^ T_reg_ signature genes identified in mice (*17*). These mouse genes were converted to homolog genes in humans and constituted as gene sets for the subsequent GSEA analysis (*15*, *16*) in human KIR^+^ versus KIR^–^CD8^+^ T cells.

### scRNA-seq analysis of kidneys and synovial tissues

The unique molecular identifier (UMI) count matrices of cells in kidneys (accession code SDY997) (*50*) or synovial tissues (accession code SDY998) (*51*) generated by CEL-Seq2 were downloaded from the ImmPort repository, and downstream analysis was performed using the Seurat 3.0 package (*52*). Cells with fewer than 1000 detected genes, more than 5000 detected genes, or more than 25% mitochondrial genes were discarded. CD8^+^ T cells (expressing *CD3D*, *CD3E*, *CD8A*, and *CD8B* transcripts) and CD4^+^ T cells (expressing *CD3D*, *CD3E*, and *CD4* transcripts) were selected for standard downstream procedures of log-normalization, variable gene selection, and data scaling.

### scRNA-seq analysis of bronchoalveolar immune cells

Filtered expression matrix of scRNA-seq of immune cells from the bronchoalveolar lavage fluid of six severe and three moderate COVID-19 patients and three HCs generated by 10X Genomics (*23*) were downloaded from Gene Expression Omnibus under the accession number GSE145926. CD8^+^ T cells were identified for downstream analysis using the Seurat 3.0 package (*52*).

### scRNA-seq analysis of blood CD8^+^ T cells by 10X Genomics

scRNA-seq of T cells from the blood of healthy subjects (*N* = 10), MS patients (*N* = 6), and COVID-19 patients (*N* = 25; ArrayExpress: E-MTAB-9357) (*24*) from the microfluidic droplet platform (10X Genomics Chromium Single Cell 5′-paired-end chemistry) were demultiplexed, aligned to the GRCh38 reference genome, and converted into gene counts matrices using CellRanger 3.1.0. Downstream analysis was performed using the Seurat 3.0 package (*52*). Cells with fewer than 800 detected genes, more than 3000 detected genes, or more than 10% mitochondrial genes were discarded. CD8^+^ T cells (expressing *CD8A* and *CD8B* but not *TRDC* transcripts) were selected for further analysis. To make counts comparable among cells, gene counts were normalized to 10,000 reads per cell, then log-transformed. We identified highly variable genes for each individual, then integrated gene expression data from all individuals using Seurat’s integration anchor discovery algorithm (*53*). We performed principal components analysis (PCA) dimensionality reduction on the integrated data, then clustered cells with the Louvain algorithm and visualized the data using uniform manifold approximation and projection (UMAP). We identified canonical cell type marker genes that were conserved across conditions using the Wilcoxon rank-sum test implemented in the Seurat package’s “FindConservedMarkers” function.

### Quantification of scRNA-seq gene expression by Smart-seq2 and data analysis

Blood KIR^+^CD8^+^ T cells (live CD3^+^CD56^–^CD8^+^TCRαβ^+^KIR^+^ cells) were sorted into 96-well plates, and cDNA synthesis was performed using the Smart-seq2 protocol (*26*) with minor modifications described previously (*54*). cDNA products were purified with 0.65X AMPure XP beads (Beckman Coulter) on the Biomek FXᴾ Automated Workstation (Beckman Coulter) and eluted with 25 μl of water. Then 2 μl of the purified products were subjected to quality control using capillary electrophoresis on a Fragment Analyzer (Agilent Technologies) by Stanford Protein and Nucleic Acid Facility.

cDNA in 96-well plates was transferred into 384-well low volume serial dilution (LVSD) plates (TTP Labtech) and diluted to 160 ng/ml using a Mosquito X1 liquid handler (TTP Labtech). Illumina sequencing libraries were prepared as described previously (*55*) using a Mosquito HTS liquid handler (TTP Labtech). After library preparation, wells of each library plate were pooled using a Mosquito HTS liquid handler (TTP Labtech). Pooling was followed by two purifications using 0.65X and 1X AMPure XP beads (Beckman Coulter), respectively. Library quality was assessed by Agilent 2100 Bioanalyzer and normalized to 5 nM. Libraries were sequenced on the Hiseq4000 Sequencing System (Illumina) in Stanford Functional Genomics Facility, acquiring 150-bp paired-end reads. FASTQ files for each cell were extracted and generated, distinguished by the unique dual index adapters. Reads were aligned to the GRCh38 genome using STAR v2.6.1d. Transcript abundance was quantified using HTSeq v0.5.4p5.

Standard procedures for filtering, log-normalization, variable gene selection, dimensionality reduction, and clustering were performed using the Seurat 3.0 package (*52*). Briefly, cells with fewer than 800 detected genes, more than 5000 detected genes, or more than 15% mitochondrial genes were discarded. To make counts comparable among cells, gene counts were normalized to 10,000 reads per cell, then log-transformed. After PCA dimensionality reduction, cells were clustered by running the Louvain algorithm and visualized using UMAP. Differential expression analysis was performed using the Wilcoxon rank-sum test implemented in the Seurat package’s “FindAllMarkers” function. Significant DEGs were defined as those with log fold change >0.5 and Bonferroni-corrected *P* < 0.05.

### Single-cell TCR-seq

TCR sequencing (TCR-seq) was performed using our previously developed single-cell paired TCR-seq method (*27*). For the first TCR reaction, 1 μl of the cDNA products of Smart-seq2 was preamplified with HotStarTaq DNA polymerase (Qiagen) using multiplex PCR with multiple Vα and Vβ region primers, Cα and Cβ region primers. Three steps of PCR were performed followed by purification of 350- to 380-bp products using a Qiaquick gel extraction kit (Qiagen). The purified product was then sequenced on a Miseq platform (Illumina) acquiring 2 × 250 bp reads.

### Bulk TCRβ sequencing

KIR^+^CD8^+^ T cells were sorted from PBMCs of nine healthy subjects, and DNA was extracted using QIAamp DNA Micro Kit (Qiagen). Sequencing of the CDR3 regions of human TCRβ chains was performed using the immunoSEQ Assay by Adaptive Biotechnologies.

### GLIPH2 analysis

Single-cell TCR sequences of sorted KIR^+^ (6815 unique TCRs) and KIR^–^CD8^+^ T cells (1630 unique TCRs) from HCs (*N* = 10), MS (*N* = 2), SLE (*N* = 20), CeD (*N* = 5), T1D (*N* = 5), and COVID-19 (*N* = 5) patients and bulk TCRβ sequences of sorted KIR^+^CD8^+^ T cells from nine HCs (5607 unique TCRβ) along with their class I HLA alleles were used as inputs. The GLIPH2 analysis generated 982 clusters, and 668 of them were shared between any two sources. We further filtered the resulting GLIPH clusters to 62 specificity groups that contained TCRs from three or more individuals and exhibited significant bias of V-gene usage (*P* < 0.05), and some of them are shown in Table 1.

### In vitro cell proliferation assay

CD8^+^ T cells were purified from PBMCs of healthy donors using CD8 microbeads (Miltenyi) per manufacturer’s instructions, stained with flow antibodies, and live CD3^+^CD56^–^CD8^+^KIR^+^ or KIR^–^ T cells were sorted out by FACSAria Fusion flow cytometer (BD). The sorted KIR^+^ or KIR^–^CD8^+^ T cells were stimulated with anti-CD3/CD28 beads (Gibco) at 1:1 ratio (1 μl beads per 4 × 10^4^ cells) supplemented with 50 U/ml of IL-2 in 96-well plates for 18 hours. The CD8^–^ PBMCs were labeled with CellTrace Violet (CTV, ThermoFisher) per manufacturer’s instruction. 1 μg/ml of anti-CD3 (UCHT-1) was coated on 96-well plate in 50 μl of PBS per well at 4°C overnight. After removal of anti-CD3/CD28 microbeads, KIR^+^ and KIR^−^ CD8^+^ T cells were mixed with CTV-labeled CD8^−^ PBMCs at 1:30, 1:15, or 1:10 ratios and cultured in 96-well plate precoated with anti-CD3. After 3 days, cells were harvested and dilution of CTV in CD4^+^ T cells was analyzed by flow cytometry.

### LCMV-Armstrong infection

Two-month-old female *Klra6*^cre^DTA mice and ROSA-DTA (wild-type) littermates were infected with 2 × 10^5^ PFU LCMV-Armstrong through intraperitoneal injection. On day 30, mice were euthanized, and spleen lymphocytes were subjected to flow cytometric analysis. After kidneys were harvested, one of them was fixed with formalin and embedded in paraffin, and the other was embedded in optimal cutting temperature (OCT) compound and flash frozen.

### Murine influenza virus infection

Influenza A/PR/8/34 H1N1 was obtained from Charles River (cat. no. 10100374, lot no. 4XP201023), aliquoted and stored in –80°C. A new aliquot was thawed and diluted with sterile PBS for infection each time. Twenty-week-old male *Klra6*^cre^DTA mice and ROSA-DTA littermates were anesthetized and intratracheally infected with a sublethal dose of influenza A/PR/8/34 H1N1 in 20 μl of PBS. Mice were euthanized on day 60 and lungs were harvested, fixed with formalin, and embedded in paraffin.

### Immunohistochemistry

To evaluate the glomerular nephritis in the kidneys of LCMV-infected mice, tissue sections were generated from formalin-fixed paraffin-embedded (FFPE) kidneys and Periodic acid–Schiff (PAS) stain was performed by Stanford Animal Histology Services. For analysis of IgG deposition in kidney, frozen sections from mouse kidneys were stained with Alexa Fluor 488 Goat anti-Mouse IgG (H+L) (Invitrogen), and images were acquired using Leica SP8. Quantification of fluorescence intensity was performed using the ImageJ software. To assess immunopathology in the lungs of influenza-infected mice, tissue sections were generated from FFPE lung tissues and stained with hematoxylin and eosin (H&E).

### Quantification of viral load by RT-qPCR

RNA was extracted from the blood of LCMV-infected mice or the lungs of influenza-infected mice using RNeasy Mini Kit (Qiagen) and converted to cDNA using Applied Biosystems High-Capacity cDNA Reverse Transcription Kit (cat. no. 43-688-14). The generated cDNA was then amplified by quantitative PCR (qPCR) using Platinum SYBR Green qPCR SuperMix-UDG w/ROX (cat. no. 11744100) per manufacturer’s instructions with the following primers: Beta Actin primer 1: 5′-CGA GGC CCA GAG CAA GAG AG-3′, Beta Actin primer 2: 5′-CGG TTG GCC TTA GGG TTC AG-3′, LCMV GP forward primer: 5′-TGC CTG ACC AAA TGG ATG ATT-3′, LCMV GP reverse primer: 5′-CTG CTG TGT TCC CGA AAC ACT-3′, NS1 forward primer: 5′-TGT CAA GCT TTC AGG TAG ATT G-3′, NS-1 reverse primer: 5′-CTC TTA GGG ATT TCT GAT CTC-3′, M1 forward primer: 5′-AAG ACC AAT CCT GTC ACC TCT GA-3′, and M1 reverse primer: 5′-CAA AGC GTC TAC GCT GCA GTC C-3′.

### Statistical analysis

No specific statistical methods were used to predetermine sample size. No samples or data points were excluded from the analysis. All results are presented as the means ± SEMs. The significance of the difference between groups was analyzed as described in the figure legends. *P* values <0.05 were considered statistically significant. All statistical analyses were performed using GraphPad Prism Software version 9.1.0.

## Supplementary Material

20220308-1

## Supplementary Materials

### Other Supplementary Material for this manuscript includes the following:

Tables S1 to S7

MDAR Reproducibility Checklist

## Notes

View/request a protocol for this paper from *Bio-protocol*.
