# Supplementary material for: KIR+CD8+ T cells suppress pathogenic T cells and are active in autoimmune diseases and COVID-19
Source: Science. 2022 Mar 8;376(6590):eabi9591. doi: 10.1126/science.abi9591 (PMC8995031; doi:10.1126/science.abi9591)
Supplement: 20220308-1 [file science.abi9591.v1.pdf]

Cite as: J. Li *et al.*, *Science* 10.1126/science.abi9591 (2022).

KIR⁺CD8⁺ T cells suppress pathogenic T cells and are active in autoimmune diseases and COVID-19

Jing Li[1], Maxim Zaslavsky[2], Yapeng Su[3], Jing Guo[4], Michael J. Sikora[5], Vincent van Unen[1], Asbjørn Christophersen[6,7,8], Shin-Heng Chiou[1]†, Liang Chen[1], Jiefu Li[9], Xuhuai Ji[10], Julie Wilhelmy[1], Alana M. McSween[1]‡, Brad A. Palanski[11]§, Venkata Vamsee Aditya Mallajosyula[1], Nathan A. Bracey[1], Gopal Krishna R. Dhondalay[12], Kartik Bhamidipati[13], Joy Pai[13], Lucas B. Kipp[14], Jeffrey E. Dunn[14], Stephen L. Hauser[15], Jorge R. Oksenberg[15], Ansuman T. Satpathy[16], William H. Robinson[17,18], Cornelia L. Dekker[19], Lars M. Steinmetz[5,20,21], Chaitan Khosla[11,22,23], Paul J. Utz[1,18], Ludvig M. Sollid[6,7,8,24], Yueh-Hsiu Chien[1,4], James R. Heath[3,25], Nielsen Q. Fernandez-Becker[26], Kari C. Nadeau[1,12], Naresha Saligrama[1]¶*, Mark M. Davis[1,9,27]*

[1]Institute of Immunity, Transplantation and Infection, Stanford University School of Medicine, Stanford, CA, USA. [2]Program in Computer Science, Stanford University, Stanford, CA, USA. [3]Institute for Systems Biology, Seattle, WA, USA. [4]Department of Microbiology and Immunology, Stanford University School of Medicine, Stanford, CA, USA. [5]Department of Genetics, Stanford University School of Medicine, Stanford, CA, USA. [6]K.G. Jebsen Coeliac Disease Research Centre, University of Oslo, Oslo, Norway. [7]Institute of Clinical Medicine, University of Oslo, Oslo, Norway. [8]Department of Immunology, University of Oslo, Oslo, Norway. [9]The Howard Hughes Medical Institute, Stanford University School of Medicine, Stanford, CA, USA. [10]Human Immune Monitoring Center, Stanford University School of Medicine, Stanford, CA, USA. [11]Department of Chemistry, Stanford University, Stanford, CA, USA. [12]Sean N. Parker Center for Allergy and Asthma Research, Department of Medicine, Stanford University, Stanford, CA, USA. [13]Program in Immunology, Stanford University School of Medicine, Stanford, CA, USA. [14]Division of Neuroimmunology, Department of Neurology and Neurological Sciences, Stanford University School of Medicine, Stanford, CA, USA. [15]Department of Neurology and UCSF Weill Institute for Neurosciences, University of California, San Francisco, CA, USA. [16]Department of Pathology, Stanford University School of Medicine, Stanford, CA, USA. [17]VA Palo Alto Health Care System, Palo Alto, CA, USA. [18]Division of Immunology and Rheumatology, Department of Medicine, Stanford University, Stanford, CA, USA. [19]Department of Pediatrics, Stanford University School of Medicine, Stanford, CA, USA. [20]Stanford Genome Technology Center, Stanford University, Palo Alto, CA, USA. [21]European Molecular Biology Laboratory (EMBL), Genome Biology Unit, Heidelberg, Germany. [22]Department of Chemical Engineering, Stanford University, Stanford, CA, USA. [23]Stanford ChEM-H, Stanford University, Stanford, CA, USA. [24]Department of Immunology, Oslo University Hospital, Oslo, Norway. [25]Department of Bioengineering, University of Washington, Seattle, WA, USA. [26]Division of Gastroenterology and Hepatology, Department of Medicine, Stanford University, Stanford, CA, USA. [27]Department of Microbiology and Immunology, Stanford University School of Medicine, Stanford, CA, USA.

†Present address: Rutgers Cancer Institute of New Jersey, Robert Wood Johnson Medical School, Rutgers, The State University of New Jersey, New Brunswick, NJ, USA. ‡Present address: Immunology Graduate Program, University of Pennsylvania, Perelman School of Medicine, Philadelphia, PA, USA. §Present address: Department of Medicine, Division of Genetics, Brigham and Women's Hospital, and Department of Biological Chemistry and Molecular Pharmacology, Harvard Medical School, Boston, MA, USA. ¶Present address: Department of Neurology, Washington University School of Medicine in St. Louis, St. Louis, MO, USA.

***Corresponding author. Email: nareshas@wustl.edu (N.S.); mmdavis@stanford.edu (M.M.D.)**

Here we find that CD8⁺ T cells expressing inhibitory killer cell immunoglobulin-like receptors (KIRs) are the human equivalent of Ly49⁺CD8⁺ regulatory T cells in mice and are increased in the blood and inflamed tissues of patients with a variety of autoimmune diseases. Moreover, these CD8⁺ T cells efficiently eliminated pathogenic gliadin-specific CD4⁺ T cells from celiac disease patients' leukocytes in vitro. We also find elevated levels of KIR⁺CD8⁺ T cells, but not CD4⁺ regulatory T cells, in COVID-19 patients, which correlated with disease severity and vasculitis. Selective ablation of Ly49⁺CD8⁺ T cells in virus-infected mice led to autoimmunity post infection. Our results indicate that in both species, these regulatory CD8⁺ T cells act uniquely to suppress pathogenic T cells in autoimmune and infectious diseases.

Although most CD8⁺ T cells are geared toward the control of pathogen-infected or cancerous cells, there has been long-standing evidence in mice that a small subset can also suppress autoimmune responses (*1*). This regulatory function of CD8⁺ T cells was first implicated by the depletion of CD8⁺ T cells in experimental autoimmune encephalomyelitis (EAE), a mouse model of human multiple sclerosis (MS) (*2*, *3*). In particular, disruption of Qa-1-CD8 coreceptor binding in B6.Qa-1-D227K mice leads to spontaneous autoimmune diseases (*4*). The Ly49 family of inhibitory C-type lectin-like receptors, which are ubiquitous on natural killer (NK) cells, were identified as unique surface markers for this regulatory CD8⁺ T cell subset (*5*). Moreover, the transcription factor Helios is an essential control element for their differentiation and function in mice (*6*). Recently, our group found that clonally expanded CD8⁺ T cells in EAE recognized peptides bound to H2-D^b and that these peptides stimulate Ly49⁺CD8⁺ regulatory T cells and suppress disease (*7*). This extended the original observations beyond Qa-1 to encompass classical class I MHC interactions, suggesting a general mechanism of peripheral tolerance. Here, we identify CD8⁺ T cells expressing inhibitory killer cell immunoglobulin-like

receptors (KIRs), the functional counterpart of the mouse Ly49 family in humans (*8*), as a regulatory CD8$^+$ T cell subset in humans that suppresses pathogenic CD4$^+$ T cells in celiac disease (CeD), and likely other autoimmune disorders and infectious diseases as well.

Increased KIR$^+$CD8$^+$ T cells in human autoimmune diseases

Both mouse Ly49 and human KIR receptors are known to bind to class I MHC molecules (*8*). They typically have inhibitory tyrosine-based inhibition motifs (ITIMs) in their cytoplasmic tails and are ubiquitously expressed on NK cells, as well as a small subset (1 to 5%) of CD8$^+$ T cells (*5*). Therefore, we analyzed CD8$^+$ T cells expressing inhibitory KIRs (hereby designated as KIR$^+$CD8$^+$ T cells) (*9*, *10*) in the peripheral blood of patients with autoimmune diseases and age- and gender-matched healthy controls (HC). KIR3DL1 and KIR2DL3 were the two major KIR subtypes expressed by human CD8$^+$ T cells (fig. S1). The frequency of KIR$^+$CD8$^+$ T cells was significantly increased in the blood of patients with MS, systemic lupus erythematosus (SLE), or CeD, compared to healthy controls (Fig. 1A).

Next, we investigated whether KIR$^+$CD8$^+$ T cells were also present in the inflamed tissues of patients with these diseases. CeD patients with active disease had higher levels of KIR$^+$CD8$^+$ T cells in the gut than those in remission (on a gluten-free diet) as well as the non-CeD controls (Fig. 1B), indicating a strong correlation of KIR$^+$CD8$^+$ T cell levels with disease severity. In addition, the number of KIR$^+$CD8$^+$ T cells was markedly increased in the kidneys of patients with SLE compared to healthy kidneys (Fig. 1C), and in the synovial tissues of RA patients compared to those with osteoarthritis (OA) which is not thought to be an autoimmune disease. By contrast, the frequencies of synovial FOXP3$^+$CD4$^+$ regulatory T cells (Tregs) were similar between RA and OA (Fig. 1D).

KIR$^+$CD8$^+$ T cells are the functional and phenotypic equivalent of mouse Ly49$^+$CD8$^+$ T cells

We next asked whether KIR$^+$CD8$^+$ T cells are the functional counterpart of mouse Ly49$^+$ regulatory CD8$^+$ T cells. Previously we found that Ly49$^+$CD8$^+$ T cells suppress myelin oligodendrocyte glycoprotein (MOG)-specific pathogenic CD4$^+$ T cells in a perforin-dependent manner (*4*, *7*). Deamidated gliadin derived from dietary gluten is the antigen for CD4$^+$ T cells that drives autoimmune enteropathy in human CeD (*11*, *12*). Therefore, we explored whether KIR$^+$CD8$^+$ T cells can suppress gliadin-specific CD4$^+$ T cells from CeD patients. CD8$^-$ peripheral blood mononuclear cells (PBMCs) from HLA-DQ2.5$^+$ CeD patients were cultured with preactivated KIR$^+$ and KIR$^-$ CD8$^+$ T cells supplemented with deamidated gluten (fig. S2A). In the absence of KIR$^+$CD8$^+$ T cells, deamidated gluten profoundly stimulated the expansion of gliadin-specific CD4$^+$ T cells. Importantly, stimulated KIR$^+$CD8$^+$ T cells, but not KIR$^-$CD8$^+$ T cells or KIR$^+$NK cells, significantly reduced the number of gliadin-specific CD4$^+$ T cells (Fig. 2A and fig. S2C) without affecting the number of total CD4$^+$ T cells (fig. S2B). KIR$^+$CD8$^+$ T cells appeared to target only the pathogenic CD4$^+$ T cells, since they had no discernible effect on hemagglutinin (HA)-specific CD4$^+$ T cells induced by influenza A HA peptides (fig. S2D) or the proliferation of CD4$^+$ T cells following anti-CD3 stimulation (fig. S2E). The suppression by KIR$^+$CD8$^+$ T cells was contact-dependent since their inhibitory effects on gliadin-specific CD4$^+$ T cells were abrogated when they were separated from the CD8$^-$ PBMCs by a membrane insert (Fig. 2C). We also found increased Annexin V binding on gliadin-specific CD4$^+$ T cells in the presence of KIR$^+$CD8$^+$ T cells (Fig. 2B), indicating these cells can induce apoptosis of the pathogenic CD4$^+$ T cells. In the presence of high-dose IL-2, KIR$^+$CD8$^+$ T cells were still able to reduce the number of gliadin-specific CD4$^+$ T cells (fig. S2F). Thus, KIR$^+$CD8$^+$ T cells suppress pathogenic CD4$^+$ T cells through direct killing instead of a competition for IL-2, consistent with the perforin dependance of Ly49$^+$CD8$^+$ T cells in mice (*4*, *7*).

Previous studies have suggested that the regulatory function of mouse Ly49$^+$CD8$^+$ T cells is mediated by recognition of both classical (*7*) and non-classical MHC I (*4*, *5*, *13*) on their target cells. Pre-activated KIR$^+$CD8$^+$ T cells showed more potent suppression of gliadin-specific CD4$^+$ T cells than the untreated KIR$^+$CD8$^+$ T cells (Fig. 2D), indicating TCR activation is required to fully elicit their suppressive functions. Moreover, anti-HLA-ABC and anti-HLA-E blockade could partially reverse the suppression by KIR$^+$CD8$^+$ T cells (Fig. 2E). Thus, KIR$^+$CD8$^+$ T cells specifically eliminate gliadin-specific CD4$^+$ T cells from the leukocytes of CeD patients via the recognition of classical and/or non-classical MHC I molecules.

To further investigate whether KIR$^+$CD8$^+$ T cells are the phenotypic equivalent of mouse Ly49$^+$ T cells in humans, we performed RNA sequencing (RNA-seq) analysis on KIR$^+$ versus KIR$^-$ CD8$^+$ T cells from patients with MS to compare with mouse Ly49$^+$CD8$^+$ T cells in EAE, a mouse model of human MS. There were 778 differentially expressed genes (DEGs) between KIR$^+$ and KIR$^-$CD8$^+$ T cells (table S1). Notably, KIR$^+$CD8$^+$ T cells showed a marked up-regulation of cytotoxic molecules, NK cell-associated genes, and cell-trafficking molecules, in addition to inhibitory KIR receptor genes (fig. S3A). Furthermore, KIR$^+$CD8$^+$ T cells expressed higher transcript levels for Helios (encoded by *IKZF2*), a transcription factor associated with regulatory functions of both CD4$^+$ and CD8$^+$ T cells (*6*). KIR$^+$CD8$^+$ T cells also down-regulated naïve/memory T cell-associated molecules and the costimulatory receptor *CD28* (fig. S3A), which is one of the key features for regulatory CD8$^+$ T cell populations in mice and humans (*14*). Gene ontology enrichment analysis of these DEGs

showed enrichment for T cell activation, proliferation, migration, and differentiation (fig. S3B). Moreover, gene set enrichment analysis (GSEA) (*15*, *16*) revealed that approximately half of the top 200 genes up-regulated in Ly49$^+$CD8$^+$ T cells were also higher in KIR$^+$CD8$^+$ T cells (fig. S3C). Previously, we found Ly49$^+$CD8$^+$ T cells expressed 16 out of 60 of the genes conserved in CD4$^+$ Tregs (*7*). These same Treg signature genes (*17*) were also enriched in KIR$^+$CD8$^+$ T cells (fig. S3D). Thus, human KIR$^+$CD8$^+$ T cells share many similarities with Ly49$^+$CD8$^+$ T cells from EAE mice.

RNA-seq analysis of KIR$^+$ versus KIR$^-$ CD8$^+$ T cells from healthy subjects and patients with different autoimmune diseases (including MS, SLE, and CeD) identified a set of 963 DEGs. Many of them overlapped with the DEGs previously defined in MS (fig. S3E and table S2), but larger-fold changes were observed in patients with higher frequencies of KIR$^+$CD8$^+$ T cells (fig. S4A). Consistent with the transcriptional profiles, KIR$^+$CD8$^+$ T cells had higher protein expression of granzyme B, perforin, CX3CR1, KLRG1, CD244, TIGIT, T-bet, and Helios and lower levels of CCR7, CD27, and CD28 (fig. S4B). Similar to the circulating KIR$^+$CD8$^+$ T cells, both kidney and synovial KIR$^+$CD8$^+$ T cells up-regulated *KLRG1*, *CD244*, *TIGIT*, *CX3CR1*, *PRF1*, *GZMB*, and *IKZF2*, while down-regulating *CD28* and *CCR7* (fig. S5). Thus, KIR$^+$CD8$^+$ T cells appear to be the functional and phenotypic equivalent of mouse Ly49$^+$CD8$^+$ T cells in humans, with many conserved features in both healthy subjects and those with autoimmune diseases.

Since the KIR$^+$CD8$^+$ T cells we focused on in this study express inhibitory KIR receptors, which contain intracellular ITIMs, we asked how these KIR receptors contribute to the differentiation and/or functionality of KIR$^+$CD8$^+$ T cells. CD8$^+$ T cells with low KIR3DL1 or KIR2DL3 expression displayed higher surface CX3CR1 and intracellular granzyme B and perforin compared to those with high KIR expression (fig. S6, A to B). RNA-seq followed by gene ontology enrichment analysis confirmed that CD8$^+$ T cells with low KIR expression displayed enhanced cytotoxicity and T cell activation (fig. S6, C to D). Thus, the high expression of inhibitory KIR receptors suppresses the activation and effector functions of the KIR$^+$CD8$^+$ T cells. This may allow for the precise control of their activity to avoid bystander suppression.

Increased KIR$^+$CD8$^+$ T cells in SARS-CoV-2- and influenza-infected patients

Although it was previously accepted that most self-specific T cells were eliminated in the thymus, recent work has shown that many such cells survive and populate the periphery of both humans and mice (*18*, *19*). We have hypothesized that this occurs because the threat of infectious diseases (*20*) necessitates a complete T cell repertoire (*18*, *21*) such that even self-reactive T cells might be needed in the response to a particular pathogen. Consistent with this are classic experiments showing that infectious diseases or treatments that mimic them can activate self-specific T cells (*22*). This led us to hypothesize that KIR$^+$CD8$^+$ T cells might be elevated during an infection to control autoreactive T cells. We first analyzed 56 patients with coronavirus disease 2019 (COVID-19). We found that KIR$^+$CD8$^+$ T cells were substantially elevated in many of these patients and higher levels correlated with more severe disease (Fig. 3A). Moreover, the highest frequencies of KIR$^+$CD8$^+$ T cells were found in patients with vasculitis or embolism and to a lesser extent in those with acute respiratory distress syndrome (ARDS) (Fig. 3B and fig. S7, C to D), which are common complications of this disease likely caused by excessive inflammation. Thus, increased levels of KIR$^+$CD8$^+$ T cells may be prognostic for autoimmune-related pathologies during a severe acute respiratory syndrome coronavirus 2 (SARS-CoV-2) infection. By contrast, we did not observe any difference in the levels of CD25^{hi}CD127^{lo}CD4$^+$ Tregs or KIR$^+$ NK cells in COVID-19 patients compared to healthy donors or in COVID-19 patients with different disease severities or complications (fig. S7, A to D, and Fig. 3B). Utilizing publicly available single-cell RNA-seq data (*23*), we also found an increased frequency of KIR$^+$CD8$^+$ T cells in the bronchoalveolar lavage fluid of COVID-19 patients with moderate or severe disease compared to that from healthy controls (Fig. 3C). In addition, an increased frequency of KIR$^+$CD8$^+$ T cells but not CD4$^+$ Tregs was observed in the peripheral blood of influenza-infected patients (Fig. 3D), suggesting that KIR$^+$CD8$^+$ T cells are generally induced as part of an infectious disease response.

Commonality and heterogeneity of KIR$^+$CD8$^+$ T cells

We next performed single-cell RNA-seq analysis on peripheral blood CD8$^+$ T cells from healthy subjects, MS patients and COVID-19 patients (*24*) using the 10X Genomics platform (*25*). KIR$^+$CD8$^+$ T cells from different conditions formed a distinct cluster with high expression of effector genes as well as *KIR* transcripts (Fig. 4, A to B, and table S3). Compared to KIR$^-$ effector CD8$^+$ T cells, KIR$^+$ effector CD8$^+$ T cells expressed higher levels of *IKZF2* and NK cell-associated genes (e.g., *TYROBP*, *KLRC2*, *KLRC3*, *NCR1*, and *NCR3*), while expressing lower levels of *IL7R*, *CD27*, and *KLRB1* (table S3). Thus, these findings reveal the commonality of KIR$^+$CD8$^+$ T cells across physiological and diseased status as well as their uniqueness relative to other CD8$^+$ T cells.

In order to better understand the similarity and heterogeneity of KIR$^+$CD8$^+$ T cells under different circumstances, we performed single-cell RNA-seq on 4512 KIR$^+$CD8$^+$ T cells sorted from healthy subjects and patients with MS, SLE, or CeD using the Smart-seq2 protocol (*26*) and analyzed their T-cell receptor (TCR) α and β sequences (*27*). Unsupervised clustering of these KIR$^+$CD8$^+$ T cells identified six clusters,

with Clusters 1 to 3 mostly containing expanded KIR$^+$CD8$^+$ T cells (≥2 cells expressing same TCR) and Clusters 5 and 6 consisting of unexpanded cells expressing unique TCRs (Fig. 4C and fig. S8, A to B). Expanded KIR$^+$ cells in Clusters 1 to 3 had higher transcripts for cytotoxic molecules (e.g., *GZMH*, *GZMB*, and *PRF1*) and genes associated with effector T cells (e.g., *FCGR3A*, *FGFBP2*, and *CX3CR1*). Cluster 2, which was more restricted to expanded KIR$^+$ cells from MS patients, showed higher levels of type I IFN responding genes. Cluster 3—specific to expanded KIR$^+$ cells from a subset of HC and SLE patients—showed higher expression of genes involved in glycolysis (Fig. 4, D to F, and table S4). Cells in Cluster 4 were in a transitional state with a loss of memory-associated features. Clusters 5 and 6 displayed memory and naïve signatures, respectively (Fig. 4F and table S4), and accounted for a small proportion of total KIR$^+$CD8$^+$ T cells (fig. S8A). T cell clones expressing identical TCRs could be found in different clusters, indicating possible lineage relationships. In addition, clonally expanded KIR$^+$CD8$^+$ T cells in COVID-19 patients identified from the previous 10X Genomics scRNA-seq (*24*) displayed a higher expression of cytotoxic genes while downregulating naïve- or memory-associated genes compared to unexpanded KIR$^+$CD8$^+$ T cells (fig. S8, C to D). In the assay with celiac PBMCs, CCR7$^-$ effector KIR$^+$CD8$^+$ T cells displayed stronger suppressive activity against gliadin-specific CD4$^+$ T cells than CCR7$^+$KIR$^+$CD8$^+$ T cells (fig. S2G), which was consistent with their up-regulated cytotoxic functions as revealed by the scRNA-seq. Thus, in parallel with clonal expansion, KIR$^+$CD8$^+$ T cells may lose their naïve or memory attributes, enter the differentiation program for effector T cells, and then suppress pathogenic CD4$^+$ T cells via cytotoxicity. There are common features shared by KIR$^+$CD8$^+$ T cells from healthy subjects and different diseases, yet there is also heterogeneity associated with different diseases or treatments.

We next compared the diversity of KIR$^+$CD8$^+$ TCRs to KIR$^-$CD8$^+$ TCRs from the same individuals (N=26) and found that TCRs of KIR$^+$CD8$^+$ T cells had significantly lower Shannon–Wiener index and Chao estimates than KIR$^-$CD8$^+$ T cells (Fig. 5, A to B). Thus, TCR repertoire of KIR$^+$CD8$^+$ T cells is less diverse, consistent with a previous report that KIR$^+$CD8$^+$ T cells display a more restricted TCR Vβ chain usage (*9*). In order to compare the antigen specificities of KIR$^+$CD8$^+$ T cells from different disease types, we also analyzed the TCR sequences using GLIPH2 (*28*), which is an algorithm to cluster TCRs that recognize the same antigen in most cases. TCRs of KIR$^+$CD8$^+$ T cells from healthy donors and different diseases could be grouped into the same GLIPH clusters, although with different extent of clonal expansion (Table 1), indicating they may recognize the same antigens that commonly exist under physiological and different pathological conditions. Thus, expanded KIR$^+$CD8$^+$ T cells have shared phenotypes and antigen specificity independent of disease types. Although the analysis above shows that the TCR repertoire of KIR$^+$CD8$^+$ T cells is less diverse than KIR$^-$CD8$^+$ T cells generally, it is still considerably diverse, utilizing multiple classical HLAs and HLA-E, and probably many antigenic peptides as well.

Regulatory CD8$^+$ T cells suppress autoimmunity developed after virus infection

We next studied the impact of selective ablation of Ly49$^+$CD8$^+$ T cells on virus-infected mice. Since Ly49F (encoded by *Klra6*) is expressed on 90% Ly49$^+$CD8$^+$ T cells but only a small fraction of NK cells (*5*, *29*), we generated a *Klra6*^{cre} mouse line. In *Klra6*^{cre}R26R-EYFP mice, all of the YFP$^+$ cells expressed CD3 or NK1.1, indicating the Cre expression is restricted to NK, T, and NKT cells (fig. S9, A to B). In *Klra6*^{cre}DTA mice, there was a 50 to 75% decrease of Ly49$^+$CD8$^+$ T cells in the spleen and lymph nodes, whereas Ly49$^+$ NK cells did not show a significant reduction (fig. S9C), consistent with the preferential expression of Ly49F on CD8$^+$ T cells. *Klra6*^{cre}DTA mice did not appear to spontaneously develop any autoimmune disorders or exhibit changes in their frequencies of effector T cells, T follicular helper (Tfh) cells, or germinal center (GC) B cells for up to 8 months at age (fig. S9, D to E). When mice were infected with LCMV-Armstrong or influenza A/PR/8/34 H1N1 viruses, there was a surge of Ly49$^+$CD8$^+$ T cells in the blood of wild-type mice (Fig. 6, A and E), consistent with our previous observations of increased KIR$^+$CD8$^+$ T cells in patients with acute SARS-CoV-2 or influenza infection. However, the frequency of Ly49$^+$CD8$^+$ T cells in *Klra6*^{cre}DTA mice remained very low at all times after viral challenge, whereas Ly49$^+$ NK cells in *Klra6*^{cre}DTA mice showed only minor reduction compared to DTA (wild-type) mice (figs. S10A and S11A), indicating a selective and efficient ablation of Ly49$^+$CD8$^+$ T cells.

With either LCMV or influenza infection, *Klra6*^{cre}DTA mice showed no difference in viral clearance (figs. S10B and S11B) or levels of effector and virus-specific CD4$^+$ and CD8$^+$ T cell responses (figs. S10C and S11C) compared to controls. However, *Klra6*^{cre}DTA mice but not DTA mice developed autoimmune pathology characterized by increased numbers of Tfh and GC B cells in the spleen (Fig. 6B) as well as glomerular nephritis (Fig. 6C) and IgG deposition (Fig. 6D) in the kidney 30 days post LCMV-Armstrong infection. Similarly, influenza-infected *Klra6*^{cre}DTA mice displayed more severe inflammation and pathology characterized by peribronchial and interstitial accumulation of inflammatory cells, pulmonary consolidation, and fibrous tissue hyperplasia in the lung 60 days post influenza infection (Fig. 6E). Thus, this population of regulatory CD8$^+$ T cells appears to suppress autoimmunity that can develop after viral infection rather than having any discernable role in viral clearance.

Discussion

Here we identify KIR$^+$CD8$^+$ T cells as an important regulatory CD8$^+$ T cell subset in humans. Previous studies have shown that KIR$^+$CD8$^+$ T cells are terminally differentiated cells and display a restricted TCR repertoire (*9*, *30*), which is consistent with our findings here. Correlations between KIR$^+$CD8$^+$ T cells and tumor immune surveillance (*31*, *32*) or chronic viral infections (*33*, *34*) have been reported, but the suppressive functions of this population have not been clearly defined previously. Here we demonstrate the regulatory function of KIR$^+$CD8$^+$ T cells toward pathogenic CD4$^+$ T cells via an in vitro functional assay using PBMCs from celiac patients. This effect of KIR$^+$CD8$^+$ T cells seems specific to self-reactive or otherwise pathogenic T cells, but not CD4$^+$ T cells recognizing foreign antigens. Similar to the perforin- or Fas–FasL-dependent suppression of self-reactive CD4$^+$ T cells by murine Ly49$^+$CD8$^+$ T cells (*4*, *7*, *35*), human KIR$^+$CD8$^+$ T cells likely target pathogenic CD4$^+$ T cells via direct killing, since KIR$^+$CD8$^+$ T cells significantly up-regulate cytotoxic molecules and suppress gliadin-specific CD4$^+$ T cells in a contact-dependent manner by inducing apoptosis. In addition, the destruction of pathogenic CD4$^+$ T cells by KIR$^+$CD8$^+$ T cells appears to depend on recognition of both classical and non-classical MHC I molecules, since the blockade of either HLA-ABC or HLA-E can reverse the suppression by KIR$^+$CD8$^+$ T cells. The MHC restrictions of the KIR$^+$ regulatory CD8$^+$ T cells at work vary between individuals, though. Since KIR receptors deliver inhibitory signals through their ITIMs to suppress the activation and functions of KIR$^+$CD8$^+$ T cells, antibody-dependent blockade of KIR3DL1 or KIR2DL3 may further enhance the suppressive activity of KIR$^+$CD8$^+$ T cells toward pathogenic CD4$^+$ T cells.

We frequently observed an increased frequency of KIR$^+$CD8$^+$ T cells in the blood as well as in the inflamed tissues of patients with autoimmune disease. This increase positively correlated with disease activity in CeD intestinal biopsies. The expansion of KIR$^+$CD8$^+$ T cells in the context of autoimmune diseases may act as a negative feedback mechanism to ameliorate pathogenesis by killing autoreactive T cells. Moreover, increased KIR$^+$CD8$^+$ T cells were found in SARS-CoV-2- or influenza-infected patients and were associated with autoimmune-related complications in COVID-19 patients. This suggests that increase of KIR$^+$CD8$^+$ T cells is a general mechanism induced during an infection, which in classical murine studies has been seen to "break tolerance", that is, allow the activation of self-reactive T cells that normally require innate immune signals in addition to their cognate antigens (*36*). In mice, there was also a surge of circulating Ly49$^+$CD8$^+$ T cells following LCMV-Armstrong or influenza A/PR/8/34 H1N1 infection. Selective ablation of Ly49$^+$CD8$^+$ T cells did not interfere with antiviral immune responses but led to exacerbated autoimmunity subsequent to virus infection. This is in line with the autoimmune phenotypes secondary to LCMV infection in *Helios*$^{-/-}$ mice in which both CD8$^+$ and CD4$^+$ regulatory T cells are defective (*6*). Therefore, we hypothesize that a major role of these CD8$^+$ regulatory T cells is to control autoreactive T cells that are activated during an infection, likely because they are cross-reactive to foreign antigens. This would allow an organism to have the benefit of a complete T cell repertoire, while limiting damage from the autoreactive clones. This type of peripheral tolerance is distinct from and likely complementary to the one mediated by CD4$^+$ Tregs, which represent a separate lineage of T cells and does not appear to be generally active in the human infectious diseases analyzed here or in murine infections (*37*, *38*).

Thus, although there is still much more to learn about KIR$^+$CD8$^+$ T cells, and their murine equivalents, the data presented here and in previous reports indicate that they represent an important element in peripheral tolerance and in our understanding of the relationship between autoimmunity and infectious diseases. Further characterization of this pathway and how it may break down in autoimmune diseases and severe infections like COVID-19, will be important challenges for the future. Likewise, our findings on the KIR$^+$CD8$^+$ T cells and their properties described here may be useful in understanding key cellular dynamics in immune dysregulation and in potential therapeutic approaches to suppress undesirable self-reactivity in autoimmune or infectious diseases.

Materials and methods

Human samples

Our study cohort of patients with autoimmune disorders met classification criteria for systemic lupus erythematosus (SLE) (*39*), celiac disease (CeD) (*40*), or multiple sclerosis (MS) (*41*), respectively. Collection of blood or biopsies from patients with SLE, CeD, or MS was covered by IRB-14734, IRB-20362, and IRB-36061. Blood samples from patients during influenza virus infection were obtained from patients who had influenza-like symptoms and were tested positive for influenza A virus at the Emergency Department or the Express Outpatient Clinic at Stanford Hospital, which is covered by IRB-22442. Blood from healthy subjects was requested from Stanford Blood Center or drawn from healthy volunteers under IRB-40146. The protocols listed above have been approved by the Research Compliance Office of Stanford University. PBMCs from MS patients were also obtained from the Multiple Sclerosis Center at the University of California, San Francisco (UCSF) with the protocol approved by the committee on Human Research at UCSF. Informed written consent was obtained from all participants. Detailed information of the healthy controls and patients with autoimmune diseases included in the study is provided as table S5. PBMCs were isolated from the blood through density gradient centrifugation

(Ficoll-Paque, GE Healthcare). Duodenal biopsies from CeD patients were treated twice with 6 mM EDTA in calcium/magnesium-free HBSS for 30 min at 37°C. Supernatants containing the epithelial fractions were combined, washed, and kept on ice until staining. The remaining tissues were minced and incubated with 200 μg/ml of Liberase TL and 20 U/ml of DNase I in IMDM for 30 min at 37°C. Digested cell suspensions were passed through 100-μm cell strainer, washed with complete media, and combined with the epithelial fraction for staining.

For sample collection of COVID-19 patients, enrollment included any adult with RT-PCR-positive COVID-19. Informed consent was obtained from each patient or from the patient's legally authorized representative if the patient was unable to provide consent. Participants were excluded if they were taking any experimental medications (i.e., those medications not approved by a regulatory agency for use in COVID-19). COVID-19 severity of illness was defined as described in the literature (*42*). Collection of blood from COVID-19 patients was covered by IRB-55689 and NCT04373148. Handling of COVID-19 PBMCs for flow cytometric analysis was covered under APB-3343-MD0620. The IRB and APB protocols mentioned above have been approved by the Research Compliance Office of Stanford University. Clinical metadata was obtained from Stanford clinical data electronic medical record system as per consented participant permission and definitions and diagnoses of disease were used according to Harrison's Principles of Internal Medicine, 20e. Clinical metadata for the COVID-19 patients in this study is presented in table S6.

Mice
R26R-EYFP mice (Stock No: 006148) and ROSA-DTA mice (Stock No: 009669) were obtained from the Jackson Laboratory. *Klra6*^{cre} mice were generated by Stanford Transgenic, Knockout and Tumor model Center (TKTC). *Klra6* reporter mice were generated by crossing *Klra6*^{cre} mice to R26R-EYFP mice. *Klra6*^{cre}DTA mice were generated by crossing *Klra6*^{cre} mice to ROSA-DTA mice. ROSA-DTA (wild-type) littermates were used as controls in the experiments described here. All mice were housed in the specific pathogen-free animal facilities at Stanford University. Experiments in this study were covered by the following animal protocols approved by the Animal Care and Use Committee of Stanford University: APLAC-10081, APLAC-34021 and APLAC-32763.

Flow cytometric analysis
The fluorescent dye-conjugated antibodies used for staining were listed in table S7. Frozen cell samples were thawed and washed in 10% FBS with benzonase (Sigma-Aldrich, 25 U/ml) in RPMI. After 450*g* centrifugation, cells were treated with FcR block (Biolegend, 10 μg/ml) in FACS buffer (0.5% BSA, 2 mM EDTA in PBS) for 10 min followed by staining with antibodies against surface molecules (30 min, 4°C). For intracellular staining, cells were fixed and permeabilized with the Intracellular Fixation & Permeabilization Buffer Set (eBioscience), followed by staining with antibodies against intracellular antigens (30 min, 4°C). Cells were acquired on an LSR II flow cytometer (BD), and data was analyzed using FlowJo X. Dead cells were excluded based on viability dye staining (LIVE/DEAD Fixable Near-IR Dead Cell Stain, ThermoFisher).

Functional assay
Chymotryptic gluten digests were deamidated with recombinant human transglutaminase 2, as described previously (*43*). PBMCs were isolated from blood of HLA-DQ2.5^{+} CeD patients on Day 0. CD8^{+} T cells were purified from PBMCs using CD8 microbeads (Miltenyi) per manufacturer's instructions, stained with flow antibodies, and live CD3^{+}CD56^{-}CD8^{+}KIR^{+} or KIR^{-} T cells were sorted out by FACSAria Fusion flow cytometer (BD). The sorted KIR^{+} and KIR^{-} CD8^{+} T cells were stimulated with anti-CD3/CD28 beads (Gibco) at 1:1 ratio (1 μl of beads per 4×10^{4} cells) supplemented with 50 U/ml of IL-2 in 96-well plates for 18 hours. KIR^{+} and KIR^{-} NK cells were sorted for PBMCs and rested overnight. The CD8^{-} PBMCs were stimulated with 250 μg/ml of deamidated gluten or 10 μg/ml of Influenza A HA 306-318 peptide (PKYVKQNTLKLAT) or left unstimulated at 3×10^{5} to 1×10^{6} cells/100 μl per well supplemented with 50 U/ml or 300 U/ml of IL-2. X-VIVO 15 with Gentamicin L-Gln (Lonza) supplemented with 10% human AB serum (Sigma-Aldrich) was used as culture medium. After 18 hours, anti-CD3/CD28 beads were removed from CD8^{+} T cells by a magnet and KIR^{+} or KIR^{-} CD8^{+} T cells or NK cells were added to the culture of CD8^{-} PBMCs at a 1:30 ratio. In the MHC I blockade experiments, 10 μg/ml of anti-HLA-ABC (W6/32, Biolegend), anti-HLA-E (3D12, eBioscience), or isotype controls were added to the culture. In the transwell assay, CD8^{-} PBMCs were cultured with 250 μg/ml of deamidated gluten and 50 U/ml of IL-2 in the lower chamber, while pre-activated KIR^{+}CD8^{+} T cells were added to the upper chamber of the Millicell-96 Cell Culture Insert Plate (Millipore Sigma, Cat# PSHT004R1). On Day 3, 50 U/ml of IL-2 was added to the cultures. Cells were harvested on Day 6 and stained with 10 μg/ml of HLA-DQ2.5 tetramers (*44*) complexed with four disease-relevant and immunodominant gliadin T cell epitopes (DQ2.5-glia-α1a, QLQPFPQPELPY; DQ2.5-glia-α2, PQPELPYPQPE; DQ2.5-glia-ω1, QQPFPQPEQPFP; DQ2.5-glia-ω2, FPQPEQPFPWQP) (*45*), or 10 μg/ml of HLA-DR4 tetramers complexed with Influenza A HA 306-318 peptide for 45 min at room temperature. Magnetic bead enrichment of tetramer^{+} CD4^{+} T cells was performed as previously described (*46*). Cells were washed with FACS buffer and then stained with antibodies against

surface molecules for 30 min at 4°C. After two washes with FACS buffer, 10% of the cells were reserved for FACS analysis while 90% were labeled with anti-PE microbeads and subjected to magnetic bead enrichment of PE-conjugated tetramer-positive cells using a single MACS column according to the manufacturer's protocol (Miltenyi). Cells were also harvested on Day 3 to measure Annexin V binding (BD) on gliadin-specific $CD4^+$ T cells. All cells were acquired on an LSR II flow cytometer (BD), gated on live $CD3^+CD4^+CD8^-TCR\alpha\beta^+$ cells, and analyzed using FlowJo X software. The frequency of tetramer-positive cells was calculated by dividing the number of post-enrichment $tetramer^+$ $CD4^+$ T cells by the number of $CD4^+$ T cells in the pre-enrichment sample multiplied by 9 (to account for the fact that 90% of the cells were used for the enrichment).

Bulk RNA-seq gene-expression quantification and data analysis

Live KIR^+ or KIR^- $CD8^+$ T cells were bulk sorted directly into 500 µl of TRIzol (ThermoFisher, Cat. #15596026) by FACSAria Fusion flow cytometer (BD). Total RNA was extracted from TRIzol samples using chloroform separation and isopropanol precipitation and then RNAeasy Plus Mini kit (Qiagen) for clean-up. After analysis on the 2100 Bioanalyzer, the sequencing libraries were prepared using the SMARTer Stranded RNA-seq Kit (Clontech). The resulting library was sequenced on the HiSeq 4000 platform (Illumina) in Stanford Functional Genomics Facility. For each sample in the bulk RNA sequencing library, 75-base-pair paired-end reads were acquired from the sequencer. We aligned the reads to the human reference genome (NCBI GRCh38) using STAR v2.7.0e (*47*). Gene counts were quantified and normalized (TPM) with Salmon (*48*). Differentially expressed genes were determined via the DESeq function (adjusted $P<0.05$, fold change >2) in the DESeq2 R package (*49*). Heatmaps were generated with seaborn.clustermap in python. Gene Ontology analysis plots were generated with the R package 'clusterProfiler'. To generate gene sets for gene set enrichment analysis (GSEA), we selected the top 200 genes up-regulated in $Ly49^+$ $CD8^+$ T cells compared to $Ly49^-$ $CD8^+$ T cells in EAE mice (*7*), and the previously reported $CD4^+$ Treg signature genes identified in mice (*17*). These mouse genes were converted to homolog genes in humans and constituted as gene sets for the subsequent GSEA analysis (*15*, *16*) in human KIR^+ versus KIR^- $CD8^+$ T cells.

Single-cell RNA-seq analysis of kidneys and synovial tissues

The unique molecular identifier (UMI) count matrixes of cells in kidneys (accession code SDY997) (*50*) or synovial tissues (accession code SDY998) (*51*) generated by CEL-Seq2 were downloaded from the ImmPort repository and downstream analysis was performed using the Seurat 3.0 package (*52*). Cells with fewer than 1000 detected genes, more than 5000 detected genes or more than 25% mitochondrial genes were discarded. $CD8^+$ T cells (expressing *CD3D*, *CD3E*, *CD8A*, and *CD8B* transcripts) and $CD4^+$ T cells (expressing *CD3D*, *CD3E*, and *CD4* transcripts) were selected for standard downstream procedures of log-normalization, variable gene selection, and data scaling.

Single-cell RNA-seq analysis of bronchoalveolar immune cells

Filtered expression matrix of single-cell RNA-seq of immune cells from the bronchoalveolar lavage fluid of six severe and three moderate COVID-19 patients and three healthy controls generated by 10X Genomics (*23*) were downloaded from Gene Expression Omnibus under the accession number GSE145926. $CD8^+$ T cells were identified for downstream analysis using the Seurat 3.0 package (*52*).

Single-cell RNA-seq analysis of blood $CD8^+$ T cells by 10X Genomics

Single cell RNA-seq of T cells from the blood of healthy subjects (N=10), MS patients (N=6), and COVID-19 patients (N=25, ArrayExpress: E-MTAB-9357) (*24*) from the microfluidic droplet platform (10X Genomics Chromium Single Cell 5′-paired-end chemistry) were de-multiplexed, aligned to the GRCh38 reference genome, and converted into gene counts matrices using CellRanger 3.1.0. Downstream analysis was performed using the Seurat 3.0 package (*52*). Cells with fewer than 800 detected genes, more than 3000 detected genes or more than 10% mitochondrial genes were discarded. $CD8^+$ T cells (expressing *CD8A* and *CD8B* but not *TRDC* transcripts) were selected for further analysis. To make counts comparable among cells, gene counts were normalized to 10,000 reads per cell, then log-transformed. We identified highly variable genes for each individual, then integrated gene expression data from all individuals using Seurat's integration anchor discovery algorithm (*53*). We performed PCA dimensionality reduction on the integrated data, then clustered cells with the Louvain algorithm and visualized the data using UMAP. We identified canonical cell type marker genes that were conserved across conditions using the Wilcoxon rank-sum test implemented in the Seurat package's 'FindConservedMarkers' function.

Quantification of single-cell RNA-seq gene expression by Smart-seq2 and data analysis

Blood KIR^+ $CD8^+$ T cells (Live $CD3^+CD56^-CD8^+TCR\alpha\beta^+KIR^+$ cells) were sorted into 96-well plates and cDNA synthesis was performed using the Smart-seq2 protocol (*26*) with minor modifications described previously (*54*). cDNA products were purified with 0.65X AMPure XP beads (Beckman Coulter) on

the Biomek FX^P Automated Workstation (Beckman Coulter) and eluted with 25 μl of water. Then 2 μl of the purified products were subjected to quality control using capillary electrophoresis on a Fragment Analyzer (Agilent Technologies) by Stanford Protein and Nucleic Acid Facility.

cDNA in 96-well plates was transferred into 384-well Low Volume Serial Dil. (LVSD) plates (TTP Labtech) and diluted to 160 ng/ml using a Mosquito X1 liquid handler (TTP Labtech). Illumina sequencing libraries were prepared as described previously (*55*) using a Mosquito HTS liquid handler (TTP Labtech). After library preparation, wells of each library plate were pooled using a Mosquito HTS liquid handler (TTP labtech). Pooling was followed by two purifications using 0.65X and 1X AMPure XP beads (Beckman Coulter), respectively. Library quality was assessed by Agilent 2100 Bioanalyzer and normalized to 5 nM. Libraries were sequenced on the Hiseq4000 Sequencing System (Illumina) in Stanford Functional Genomics Facility, acquiring 150-bp paired-end reads. FASTQ files for each cell were extracted and generated, distinguished by the unique dual index adaptors. Reads were aligned to the GRCh38 genome using STAR v2.6.1d. Transcript abundance was quantified using HTSeq v0.5.4p5.

Standard procedures for filtering, log-normalization, variable gene selection, dimensionality reduction and clustering were performed using the Seurat 3.0 package (*52*). Briefly, cells with fewer than 800 detected genes, more than 5000 detected genes or more than 15% mitochondrial genes were discarded. To make counts comparable among cells, gene counts were normalized to 10,000 reads per cell, then log-transformed. Following PCA dimensionality reduction, cells were clustered by running the Louvain algorithm and visualized using UMAP. Differential expression analysis was performed using the Wilcoxon rank-sum test implemented in the Seurat package's 'FindAllMarkers' function. Significantly differentially expressed genes were defined as those with log fold change >0.5 and Bonferroni-corrected *P*-value<0.05.

Single-cell TCR-seq

TCR-seq was performed using our previously developed single-cell paired TCR sequencing method (*27*). For the first TCR reaction, 1 μl of the cDNA products of Smart-seq2 was preamplified with HotStarTaq DNA polymerase (Qiagen) using multiplex PCR with multiple Vα and Vβ region primers, Cα and Cβ region primers. Three steps of PCR were performed followed by purification of 350-380 bp products using a Qiaquick gel extraction kit (Qiagen). The purified product was then sequenced on a Miseq platform (Illumina) acquiring 2×250 bp reads.

Bulk TCRβ sequencing

KIR^+CD8^+ T cells were sorted from PBMCs of nine healthy subjects, and DNA was extracted using QIAamp DNA Micro Kit (Qiagen). Sequencing of the CDR3 regions of human TCRβ chains was performed using the immunoSEQ Assay by Adaptive Biotechnologies.

GLIPH2 analysis

Single-cell TCR sequences of sorted KIR^+ (6815 unique TCRs) and KIR^- $CD8^+$ T cells (1630 unique TCRs) from healthy controls (N=10), MS (N=2), SLE (N=20), CeD (N=5), T1D (N=5), and COVID-19 (N=5) patients and bulk TCRβ sequences of sorted KIR^+CD8^+ T cells from nine healthy controls (5607 unique TCRβ) along with their class I HLA alleles were used as inputs. The GLIPH2 analysis generated 982 clusters and 668 of them were shared between any two sources. We further filtered the resulting GLIPH clusters to 62 specificity groups that contained TCRs from three or more individuals and exhibited significant bias of V-gene usage ($P<0.05$) and some of them are shown in Table 1.

In vitro cell proliferation assay

$CD8^+$ T cells were purified from PBMCs of healthy donors using CD8 microbeads (Miltenyi) per manufacturer's instructions, stained with flow antibodies, and live $CD3^+CD56^-$ $CD8^+KIR^+$ or KIR^- T cells were sorted out by FACSAria Fusion flow cytometer (BD). The sorted KIR^+ or KIR^- $CD8^+$ T cells were stimulated with anti-CD3/CD28 beads (Gibco) at 1:1 ratio (1 μl beads per $4×10^4$ cells) supplemented with 50 U/ml of IL-2 in 96-well plates for 18 hours. The $CD8^-$ PBMCs were labeled with CellTrace Violet (CTV, ThermoFisher) per manufacturer's instruction. 1 μg/ml of anti-CD3 (UCHT-1) was coated on 96-well plate in 50 μl of PBS per well at 4°C overnight. After removal of anti-CD3/CD28 microbeads, KIR^+ and KIR^- $CD8^+$ T cells were mixed with CTV-labeled $CD8^-$ PBMCs at 1:30, 1:15, or 1:10 ratios and cultured in 96-well plate precoated with anti-CD3. After 3 days, cells were harvested and dilution of CTV in $CD4^+$ T cells was analyzed by flow cytometry.

LCMV-Armstrong infection

Two-month-old female *Klra6*^cre^DTA mice and ROSA-DTA (wild-type) littermates were infected with $2×10^5$ PFU LCMV-Armstrong via intraperitoneal injection. On day 30, mice were sacrificed and spleen lymphocytes were subjected to flow cytometric analysis. After kidneys were harvested, one of them was fixed with formalin and embedded in paraffin, while the other was embedded in OCT and flash frozen.

Murine influenza virus infection

Influenza A/PR/8/34 H1N1 was obtained from Charles River (Cat# 10100374, Lot# 4XP201023), aliquoted and stored in –80°C. A new aliquot was thawed and diluted with sterile PBS for infection each time. Twenty-week-old male *Klra6*^cre^DTA mice and ROSA-DTA littermates were anesthetized and

intratracheally infected with a sublethal dose of influenza A/PR/8/34 H1N1 in 20 µl of PBS. Mice were sacrificed on Day 60 and lungs were harvested, fixed with formalin, and embedded in paraffin.

Immunohistochemistry

To evaluate the glomerular nephritis in the kidneys of LCMV-infected mice, tissue sections were generated from FFPE kidneys and Periodic acid-Schiff (PAS) stain was performed by Stanford Animal Histology Services. For analysis of IgG deposition in kidney, frozen sections from mouse kidneys were stained with Alexa Fluor 488 Goat anti-Mouse IgG (H+L) (Invitrogen) and images were acquired using Leica SP8. Quantification of fluorescence intensity was performed using the ImageJ software. To assess immunopathology in the lungs of influenza-infected mice, tissue sections were generated from FFPE lung tissues and stained with hematoxylin and eosin (H&E).

Quantification of viral load by RT-qPCR

RNA was extracted from the blood of LCMV-infected mice or the lung of influenza-infected mice using RNeasy Mini Kit (Qiagen) and converted to cDNA using Applied Biosystems High-Capacity cDNA Reverse Transcription Kit (Cat no. 43-688-14). The generated cDNA was then amplified by quantitative PCR using Platinum SYBR Green qPCR SuperMix-UDG w/ROX (Cat no. 11744100) per manufacturer's instructions with the following primers: Beta Actin primer 1: 5′-CGA GGC CCA GAG CAA GAG AG-3′, Beta Actin primer 2: 5′-CGG TTG GCC TTA GGG TTC AG-3′, LCMV GP forward primer: 5′-TGC CTG ACC AAA TGG ATG ATT-3′, LCMV GP reverse primer: 5′-CTG CTG TGT TCC CGA AAC ACT-3′, NS1 forward primer: 5′-TGT CAA GCT TTC AGG TAG ATT G-3′, NS-1 reverse primer: 5′-CTC TTA GGG ATT TCT GAT CTC-3′, M1 forward primer: 5′-AAG ACC AAT CCT GTC ACC TCT GA-3′, M1 reverse primer: 5′-CAA AGC GTC TAC GCT GCA GTC C-3′.

Statistical analysis

No specific statistical methods were used to predetermine sample size. No samples or data points were excluded from the analysis. All results are presented as the mean ± SEM. The significance of the difference between groups was analyzed as described in the figure legends. *P*-values<0.05 were considered statistically significant. All statistical analyses were performed using GraphPad Prism Software version 9.1.0.

REFERENCES AND NOTES

1. H. Nakagawa, L. Wang, H. Cantor, H. J. Kim, New Insights Into the Biology of CD8 Regulatory T Cells. *Adv. Immunol.* **140**, 1–20 (2018). doi:10.1016/bs.ai.2018.09.001 Medline
2. D. R. Koh, W.-P. Fung-Leung, A. Ho, D. Gray, H. Acha-Orbea, T.-W. Mak, Less mortality but more relapses in experimental allergic encephalomyelitis in CD8$^{-/-}$ mice. *Science* **256**, 1210–1213 (1992). doi:10.1126/science.256.5060.1210 Medline
3. H. Jiang, S. I. Zhang, B. Pernis, Role of CD8^{+} T cells in murine experimental allergic encephalomyelitis. *Science* **256**, 1213–1215 (1992). doi:10.1126/science.256.5060.1213 Medline
4. H. J. Kim, B. Verbinnen, X. Tang, L. Lu, H. Cantor, Inhibition of follicular T-helper cells by CD8^{+} regulatory T cells is essential for self tolerance. *Nature* **467**, 328–332 (2010). doi:10.1038/nature09370 Medline
5. H. J. Kim, X. Wang, S. Radfar, T. J. Sproule, D. C. Roopenian, H. Cantor, CD8^{+} T regulatory cells express the Ly49 Class I MHC receptor and are defective in autoimmune prone B6-Yaa mice. *Proc. Natl. Acad. Sci. U.S.A.* **108**, 2010–2015 (2011). doi:10.1073/pnas.1018974108 Medline
6. H. J. Kim, R. A. Barnitz, T. Kreslavsky, F. D. Brown, H. Moffett, M. E. Lemieux, Y. Kaygusuz, T. Meissner, T. A. W. Holderried, S. Chan, P. Kastner, W. N. Haining, H. Cantor, Stable inhibitory activity of regulatory T cells requires the transcription factor Helios. *Science* **350**, 334–339 (2015). doi:10.1126/science.aad0616 Medline
7. N. Saligrama, F. Zhao, M. J. Sikora, W. S. Serratelli, R. A. Fernandes, D. M. Louis, W. Yao, X. Ji, J. Idoyaga, V. B. Mahajan, L. M. Steinmetz, Y.-H. Chien, S. L. Hauser, J. R. Oksenberg, K. C. Garcia, M. M. Davis, Opposing T cell responses in experimental autoimmune encephalomyelitis. *Nature* **572**, 481–487 (2019). doi:10.1038/s41586-019-1467-x Medline
8. M. M. Rahim, A. P. Makrigiannis, Ly49 receptors: Evolution, genetic diversity, and impact on immunity. *Immunol. Rev.* **267**, 137–147 (2015). doi:10.1111/imr.12318 Medline
9. N. K. Björkström, V. Béziat, F. Cichocki, L. L. Liu, J. Levine, S. Larsson, R. A. Koup, S. K. Anderson, H.-G. Ljunggren, K.-J. Malmberg, CD8 T cells express randomly selected KIRs with distinct specificities compared with NK cells. *Blood* **120**, 3455–3465 (2012). doi:10.1182/blood-2012-03-416867 Medline
10. E. Estefanía, R. Flores, N. Gómez-Lozano, H. Aguilar, M. López-Botet, C. Vilches, Human KIR2DL5 is an inhibitory receptor expressed on the surface of NK and T lymphocyte subsets. *J. Immunol.* **178**, 4402–4410 (2007). doi:10.4049/jimmunol.178.7.4402 Medline
11. V. Abadie, L. M. Sollid, L. B. Barreiro, B. Jabri, Integration of genetic and immunological insights into a model of celiac disease pathogenesis. *Annu. Rev. Immunol.* **29**, 493–525 (2011). doi:10.1146/annurev-immunol-040210-092915 Medline
12. H. Sjöström, K. E. Lundin, O. Molberg, R. Körner, S. N. McAdam, D. Anthonsen, H. Quarsten, O. Norén, P. Roepstorff, E. Thorsby, L. M. Sollid, Identification of a gliadin T-cell epitope in coeliac disease: General importance of gliadin deamidation for intestinal T-cell recognition. *Scand. J. Immunol.* **48**, 111–115 (1998). doi:10.1046/j.1365-3083.1998.00397.x Medline
13. H. Sheng, I. Marrero, I. Maricic, S. S. Fanchiang, S. Zhang, D. B. Sant'Angelo, V. Kumar, Distinct PLZF^{+}CD8$\alpha\alpha^{+}$ Unconventional T Cells Enriched in Liver Use a Cytotoxic Mechanism to Limit Autoimmunity. *J. Immunol.* **203**, 2150–2162 (2019). doi:10.4049/jimmunol.1900832 Medline
14. Y. Vuddamalay, J. P. van Meerwijk, CD28^{-} and CD28^{low}CD8^{+} Regulatory T Cells: Of Mice and Men. *Front. Immunol.* **8**, 31 (2017). doi:10.3389/fimmu.2017.00031 Medline
15. A. Subramanian, P. Tamayo, V. K. Mootha, S. Mukherjee, B. L. Ebert, M. A. Gillette, A. Paulovich, S. L. Pomeroy, T. R. Golub, E. S. Lander, J. P. Mesirov, Gene set enrichment analysis: A knowledge-based approach for interpreting genome-wide expression profiles. *Proc. Natl. Acad. Sci. U.S.A.* **102**, 15545–15550 (2005). doi:10.1073/pnas.0506580102 Medline
16. V. K. Mootha, C. M. Lindgren, K.-F. Eriksson, A. Subramanian, S. Sihag, J. Lehar, P. Puigserver, E. Carlsson, M. Ridderstråle, E. Laurila, N. Houstis, M. J. Daly, N. Patterson, J. P. Mesirov, T. R. Golub, P. Tamayo, B. Spiegelman, E. S. Lander, J. N. Hirschhorn, D. Altshuler, L. C. Groop, PGC-1α-responsive genes involved in oxidative phosphorylation are coordinately downregulated in human diabetes. *Nat. Genet.* **34**, 267–273 (2003). doi:10.1038/ng1180 Medline
17. D. Zemmour, R. Zilionis, E. Kiner, A. M. Klein, D. Mathis, C. Benoist, Single-cell gene expression reveals a landscape of regulatory T cell phenotypes shaped by the TCR. *Nat. Immunol.* **19**, 291–301 (2018). doi:10.1038/s41590-018-0051-0 Medline
18. W. Yu, N. Jiang, P. J. R. Ebert, B. A. Kidd, S. Müller, P. J. Lund, J. Juang, K. Adachi,

T. Tse, M. E. Birnbaum, E. W. Newell, D. M. Wilson, G. M. Grotenbreg, S. Valitutti, S. R. Quake, M. M. Davis, Clonal Deletion Prunes but Does Not Eliminate Self-Specific αβ $CD8^+$ T Lymphocytes. *Immunity* **42**, 929–941 (2015). doi:10.1016/j.immuni.2015.05.001 Medline
19. L. F. Su, B. A. Kidd, A. Han, J. J. Kotzin, M. M. Davis, Virus-specific $CD4^+$ memory-phenotype T cells are abundant in unexposed adults. *Immunity* **38**, 373–383 (2013). doi:10.1016/j.immuni.2012.10.021 Medline
20. L. Quintana-Murci, Human Immunology through the Lens of Evolutionary Genetics. *Cell* **177**, 184–199 (2019). doi:10.1016/j.cell.2019.02.033 Medline
21. M. M. Davis, P. Brodin, Rebooting Human Immunology. *Annu. Rev. Immunol.* **36**, 843–864 (2018). doi:10.1146/annurev-immunol-042617-053206 Medline
22. P. S. Ohashi, S. Oehen, K. Buerki, H. Pircher, C. T. Ohashi, B. Odermatt, B. Malissen, R. M. Zinkernagel, H. Hengartner, Ablation of "tolerance" and induction of diabetes by virus infection in viral antigen transgenic mice. *Cell* **65**, 305–317 (1991). doi:10.1016/0092-8674(91)90164-T Medline
23. M. Liao, Y. Liu, J. Yuan, Y. Wen, G. Xu, J. Zhao, L. Cheng, J. Li, X. Wang, F. Wang, L. Liu, I. Amit, S. Zhang, Z. Zhang, Single-cell landscape of bronchoalveolar immune cells in patients with COVID-19. *Nat. Med.* **26**, 842–844 (2020). doi:10.1038/s41591-020-0901-9 Medline
24. Y. Su, D. Chen, D. Yuan, C. Lausted, J. Choi, C. L. Dai, V. Voillet, V. R. Duvvuri, K. Scherler, P. Troisch, P. Baloni, G. Qin, B. Smith, S. A. Kornilov, C. Rostomily, A. Xu, J. Li, S. Dong, A. Rothchild, J. Zhou, K. Murray, R. Edmark, S. Hong, J. E. Heath, J. Earls, R. Zhang, J. Xie, S. Li, R. Roper, L. Jones, Y. Zhou, L. Rowen, R. Liu, S. Mackay, D. S. O'Mahony, C. R. Dale, J. A. Wallick, H. A. Algren, M. A. Zager, ISB-Swedish COVID19 Biobanking Unit, W. Wei, N. D. Price, S. Huang, N. Subramanian, K. Wang, A. T. Magis, J. J. Hadlock, L. Hood, A. Aderem, J. A. Bluestone, L. L. Lanier, P. D. Greenberg, R. Gottardo, M. M. Davis, J. D. Goldman, J. R. Heath, Multi-Omics Resolves a Sharp Disease-State Shift between Mild and Moderate COVID-19. *Cell* **183**, 1479–1495.e20 (2020). doi:10.1016/j.cell.2020.10.037 Medline
25. G. X. Zheng, J. M. Terry, P. Belgrader, P. Ryvkin, Z. W. Bent, R. Wilson, S. B. Ziraldo, T. D. Wheeler, G. P. McDermott, J. Zhu, M. T. Gregory, J. Shuga, L. Montesclaros, J. G. Underwood, D. A. Masquelier, S. Y. Nishimura, M. Schnall-Levin, P. W. Wyatt, C. M. Hindson, R. Bharadwaj, A. Wong, K. D. Ness, L. W. Beppu, H. J. Deeg, C. McFarland, K. R. Loeb, W. J. Valente, N. G. Ericson, E. A. Stevens, J. P. Radich, T. S. Mikkelsen, B. J. Hindson, J. H. Bielas, Massively parallel digital transcriptional profiling of single cells. *Nat. Commun.* **8**, 14049 (2017). doi:10.1038/ncomms14049 Medline
26. S. Picelli, O. R. Faridani, Å. K. Björklund, G. Winberg, S. Sagasser, R. Sandberg, Full-length RNA-seq from single cells using Smart-seq2. *Nat. Protoc.* **9**, 171–181 (2014). doi:10.1038/nprot.2014.006 Medline
27. A. Han, J. Glanville, L. Hansmann, M. M. Davis, Linking T-cell receptor sequence to functional phenotype at the single-cell level. *Nat. Biotechnol.* **32**, 684–692 (2014). doi:10.1038/nbt.2938 Medline
28. H. Huang, C. Wang, F. Rubelt, T. J. Scriba, M. M. Davis, Analyzing the *Mycobacterium tuberculosis* immune response by T-cell receptor clustering with GLIPH2 and genome-wide antigen screening. *Nat. Biotechnol.* **38**, 1194–1202 (2020). doi:10.1038/s41587-020-0505-4 Medline
29. I. Tassi, R. Presti, S. Kim, W. M. Yokoyama, S. Gilfillan, M. Colonna, Phospholipase C-γ2 is a critical signaling mediator for murine NK cell activating receptors. *J. Immunol.* **175**, 749–754 (2005). doi:10.4049/jimmunol.175.2.749 Medline
30. S. A. Schattgen, K. Guion, J. C. Crawford, A. Souquette, A. M. Barrio, M. J. T. Stubbington, P. G. Thomas, P. Bradley, Integrating T cell receptor sequences and transcriptional profiles by clonotype neighbor graph analysis (CoNGA). *Nat. Biotechnol.* **40**, 54–63 (2022). doi:10.1038/s41587-021-00989-2 Medline
31. L. Gimeno, E. M. Serrano-López, J. A. Campillo, M. A. Cánovas-Zapata, O. S. Acuña, F. García-Cózar, M. V. Martínez-Sánchez, M. D. Martínez-Hernández, M. F. Soto-Ramírez, P. López-Cubillana, J. Martínez-Escribano, J. Martínez-García, S. Corbalan-García, M. R. Álvarez-López, A. Minguela, KIR+ CD8+ T Lymphocytes in Cancer Immunosurveillance and Patient Survival: Gene Expression Profiling. *Cancers* **12**, 2991 (2020). doi:10.3390/cancers12102991 Medline
32. S. Chakraborty, P. Bhattacharjee, A. K. Panda, K. Kajal, S. Bose, G. Sa, Providence of the $CD25^+KIR^+CD127^-FOXP3^-CD8^+$ T-cell subset determines the dynamics of tumor immune surveillance. *Immunol. Cell Biol.* **96**, 1035–1048 (2018). doi:10.1111/imcb.12166 Medline
33. W. Lu, S. Chen, C. Lai, M. Lai, H. Fang, H. Dao, J. Kang, J. Fan, W. Guo, L. Fu, J.-M. Andrieu, Suppression of HIV Replication by $CD8^+$ Regulatory T-Cells in Elite Controllers. *Front. Immunol.* **7**, 134 (2016). doi:10.3389/fimmu.2016.00134 Medline
34. G. Alter, S. Rihn, H. Streeck, N. Teigen, A. Piechocka-Trocha, K. Moss, K. Cohen, A. Meier, F. Pereyra, B. Walker, M. Altfeld, Ligand-independent exhaustion of killer immunoglobulin-like receptor-positive $CD8^+$ T cells in human immunodeficiency virus type 1 infection. *J. Virol.* **82**, 9668–9677 (2008). doi:10.1128/JVI.00341-08 Medline
35. K. Akane, S. Kojima, T. W. Mak, H. Shiku, H. Suzuki, $CD8^+CD122^+CD49d^{low}$ regulatory T cells maintain T-cell homeostasis by killing activated T cells via Fas/FasL-mediated cytotoxicity. *Proc. Natl. Acad. Sci. U.S.A.* **113**, 2460–2465 (2016). doi:10.1073/pnas.1525098113 Medline
36. Q. Yin, W. Yu, C. L. Grzeskowiak, J. Li, H. Huang, J. Guo, L. Chen, F. Wang, F. Zhao, L. von Boehmer, T. J. Metzner, J. T. Leppert, Y. H. Chien, C. J. Kuo, M. M. Davis, Nanoparticle-enabled innate immune stimulation activates endogenous tumor-infiltrating T cells with broad antigen specificities. *Proc. Natl. Acad. Sci. U.S.A.* **118**, e2016168118 (2021). doi:10.1073/pnas.2016168118 Medline
37. R. J. Betts, A. W. Ho, D. M. Kemeny, Partial depletion of natural $CD4^+CD25^+$ regulatory T cells with anti-CD25 antibody does not alter the course of acute influenza A virus infection. *PLOS ONE* **6**, e27849 (2011). doi:10.1371/journal.pone.0027849 Medline
38. M. C. Rogers, K. D. Lamens, N. Shafagati, M. Johnson, T. D. Oury, S. Joyce, J. V. Williams, $CD4^+$ Regulatory T Cells Exert Differential Functions during Early and Late Stages of the Immune Response to Respiratory Viruses. *J. Immunol.* **201**, 1253–1266 (2018). doi:10.4049/jimmunol.1800096 Medline
39. M. C. Hochberg, Updating the American College of Rheumatology revised criteria for the classification of systemic lupus erythematosus. *Arthritis Rheum.* **40**, 1725 (1997). doi:10.1002/art.1780400928 Medline
40. J. F. Ludvigsson, J. C. Bai, F. Biagi, T. R. Card, C. Ciacci, P. J. Ciclitira, P. H. R. Green, M. Hadjivassiliou, A. Holdoway, D. A. van Heel, K. Kaukinen, D. A. Leffler, J. N. Leonard, K. E. A. Lundin, N. McGough, M. Davidson, J. A. Murray, G. L. Swift, M. M. Walker, F. Zingone, D. S. Sanders, BSG Coeliac Disease Guidelines Development Group, Diagnosis and management of adult coeliac disease: Guidelines from the British Society of Gastroenterology. *Gut* **63**, 1210–1228 (2014). doi:10.1136/gutjnl-2013-306578 Medline
41. C. H. Polman, S. C. Reingold, B. Banwell, M. Clanet, J. A. Cohen, M. Filippi, K. Fujihara, E. Havrdova, M. Hutchinson, L. Kappos, F. D. Lublin, X. Montalban, P. O'Connor, M. Sandberg-Wollheim, A. J. Thompson, E. Waubant, B. Weinshenker, J. S. Wolinsky, Diagnostic criteria for multiple sclerosis: 2010 revisions to the McDonald criteria. *Ann. Neurol.* **69**, 292–302 (2011). doi:10.1002/ana.22366 Medline
42. G. Chen, D. Wu, W. Guo, Y. Cao, D. Huang, H. Wang, T. Wang, X. Zhang, H. Chen, H. Yu, X. Zhang, M. Zhang, S. Wu, J. Song, T. Chen, M. Han, S. Li, X. Luo, J. Zhao, Q. Ning, Clinical and immunological features of severe and moderate coronavirus disease 2019. *J. Clin. Invest.* **130**, 2620–2629 (2020). doi:10.1172/JCI137244 Medline
43. V. Abadie, S. M. Kim, T. Lejeune, B. A. Palanski, J. D. Ernest, O. Tastet, J. Voisine, V. Discepolo, E. V. Marietta, M. B. F. Hawash, C. Ciszewski, R. Bouziat, K. Panigrahi, I. Horwath, M. A. Zurenski, I. Lawrence, A. Dumaine, V. Yotova, J.-C. Grenier, J. A. Murray, C. Khosla, L. B. Barreiro, B. Jabri, IL-15, gluten and HLA-DQ8 drive tissue destruction in coeliac disease. *Nature* **578**, 600–604 (2020). doi:10.1038/s41586-020-2003-8 Medline
44. H. Quarsten, S. N. McAdam, T. Jensen, H. Arentz-Hansen, Molberg Ø, K. E. A. Lundin, L. M. Sollid, Staining of celiac disease-relevant T cells by peptide-DQ2 multimers. *J. Immunol.* **167**, 4861–4868 (2001). doi:10.4049/jimmunol.167.9.4861 Medline
45. L. M. Sollid, S. W. Qiao, R. P. Anderson, C. Gianfrani, F. Koning, Nomenclature and listing of celiac disease relevant gluten T-cell epitopes restricted by HLA-DQ molecules. *Immunogenetics* **64**, 455–460 (2012). doi:10.1007/s00251-012-0599-z Medline
46. T. J. Scriba, M. Purbhoo, C. L. Day, N. Robinson, S. Fidler, J. Fox, J. N. Weber, P. Klenerman, A. K. Sewell, R. E. Phillips, Ultrasensitive detection and phenotyping of $CD4^+$ T cells with optimized HLA class II tetramer staining. *J. Immunol.* **175**, 6334–6343 (2005). doi:10.4049/jimmunol.175.10.6334 Medline
47. A. Dobin, C. A. Davis, F. Schlesinger, J. Drenkow, C. Zaleski, S. Jha, P. Batut, M.

Chaisson, T. R. Gingeras, STAR: Ultrafast universal RNA-seq aligner. *Bioinformatics* **29**, 15–21 (2013). doi:10.1093/bioinformatics/bts635 Medline
48. R. Patro, G. Duggal, M. I. Love, R. A. Irizarry, C. Kingsford, Salmon provides fast and bias-aware quantification of transcript expression. *Nat. Methods* **14**, 417–419 (2017). doi:10.1038/nmeth.4197 Medline
49. M. I. Love, W. Huber, S. Anders, Moderated estimation of fold change and dispersion for RNA-seq data with DESeq2. *Genome Biol.* **15**, 550 (2014). doi:10.1186/s13059-014-0550-8 Medline
50. A. Arazi, D. A. Rao, C. C. Berthier, A. Davidson, Y. Liu, P. J. Hoover, A. Chicoine, T. M. Eisenhaure, A. H. Jonsson, S. Li, D. J. Lieb, F. Zhang, K. Slowikowski, E. P. Browne, A. Noma, D. Sutherby, S. Steelman, D. E. Smilek, P. Tosta, W. Apruzzese, E. Massarotti, M. Dall'Era, M. Park, D. L. Kamen, R. A. Furie, F. Payan-Schober, W. F. Pendergraft 3rd, E. A. McInnis, J. P. Buyon, M. A. Petri, C. Putterman, K. C. Kalunian, E. S. Woodle, J. A. Lederer, D. A. Hildeman, C. Nusbaum, S. Raychaudhuri, M. Kretzler, J. H. Anolik, M. B. Brenner, D. Wofsy, N. Hacohen, B. Diamond, Accelerating Medicines Partnership in SLE network, The immune cell landscape in kidneys of patients with lupus nephritis. *Nat. Immunol.* **20**, 902–914 (2019). doi:10.1038/s41590-019-0398-x Medline
51. F. Zhang, K. Wei, K. Slowikowski, C. Y. Fonseka, D. A. Rao, S. Kelly, S. M. Goodman, D. Tabechian, L. B. Hughes, K. Salomon-Escoto, G. F. M. Watts, A. H. Jonsson, J. Rangel-Moreno, N. Meednu, C. Rozo, W. Apruzzese, T. M. Eisenhaure, D. J. Lieb, D. L. Boyle, A. M. Mandelin 2nd, Accelerating Medicines Partnership Rheumatoid Arthritis and Systemic Lupus Erythematosus (AMP RA/SLE) Consortium, B. F. Boyce, E. DiCarlo, E. M. Gravallese, P. K. Gregersen, L. Moreland, G. S. Firestein, N. Hacohen, C. Nusbaum, J. A. Lederer, H. Perlman, C. Pitzalis, A. Filer, V. M. Holers, V. P. Bykerk, L. T. Donlin, J. H. Anolik, M. B. Brenner, S. Raychaudhuri, Defining inflammatory cell states in rheumatoid arthritis joint synovial tissues by integrating single-cell transcriptomics and mass cytometry. *Nat. Immunol.* **20**, 928–942 (2019). doi:10.1038/s41590-019-0378-1 Medline
52. A. Butler, P. Hoffman, P. Smibert, E. Papalexi, R. Satija, Integrating single-cell transcriptomic data across different conditions, technologies, and species. *Nat. Biotechnol.* **36**, 411–420 (2018). doi:10.1038/nbt.4096 Medline
53. T. Stuart, A. Butler, P. Hoffman, C. Hafemeister, E. Papalexi, W. M. Mauck 3rd, Y. Hao, M. Stoeckius, P. Smibert, R. Satija, Comprehensive Integration of Single-Cell Data. *Cell* **177**, 1888–1902.e21 (2019). doi:10.1016/j.cell.2019.05.031 Medline
54. H. Huang, M. J. Sikora, S. Islam, R. R. Chowdhury, Y. H. Chien, T. J. Scriba, M. M. Davis, L. M. Steinmetz, Select sequencing of clonally expanded $CD8^+$ T cells reveals limits to clonal expansion. *Proc. Natl. Acad. Sci. U.S.A.* **116**, 8995–9001 (2019). doi:10.1073/pnas.1902649116 Medline
55. Tabula Muris Consortium, Overall coordination, Logistical coordination, Organ collection and processing, Library preparation and sequencing, Computational data analysis, Cell type annotation, Writing group, Supplemental text writing group, Principal investigators, Single-cell transcriptomics of 20 mouse organs creates a *Tabula Muris*. *Nature* **562**, 367–372 (2018). doi:10.1038/s41586-018-0590-4 Medline
56. Davis Lab, davis-lab-stanford/kir-cd8: 2022-01 - v1.0.0, version 1.0.0, Zenodo (2022); https://doi.org/10.5281/zenodo.5835290.

ACKNOWLEDGMENTS

We thank members of Davis lab for helpful discussions. We also thank J. Coller in Stanford Functional Genomics Facility for NGS sequencing (NIH award S10OD018220), R. Sinha, S. D. Conley, and I. L. Weissman for assistance in single-cell RNA-seq library preparation, L. Chen for help in python scripts, D. Chen in the Institute for Systems Biology for help with the COVID-19 10X single-cell RNA-seq data processing, R. Wittman and R. Puri from the Stanford occupational health clinic, and I. Chang, J. Krempski, E. Do, M. Manohar, A. Fernandez, W. Zhang for collecting and processing blood samples from COVID-19 patients. We thank Z. He and J. Fitzpatrick for data management and entry of COVID-19 patients, D. Furman, B. Gaudillière, and D. Feyaerts for their help in obtaining COVID-19 PBMCs, and J. J. Goronzy for providing the LCMV-Armstrong strain. We thank the Stanford Protein and Nucleic Acid Facility for performing fragment analysis, the Stanford Shared FACS facility for assistance in flow cytometric analysis and cell sorting, Stanford Animal Histology Services for the histology, and the Stanford Cell Sciences Imaging Facility for assistance in microscopy. **Funding:** We are grateful for funding by the National Institute of Allergy and Infectious Diseases (NIAID) U19-AI057229 and the Howard Hughes Medical Institute to M.M.D. The collection of MS samples was supported by grant NMSS RG-1611-26299. This work was partially supported by pilot funding from the Stanford Diabetes Research Center (P30DK116074). The project described was also supported, in part, by Award Number 1S10OD010580-01A1 from the National Center for Research Resources (NCRR). Its contents are solely the responsibility of the authors and do not necessarily represent the official views of the NCRR or the National Institutes of Health (NIH). M.Z. was supported by the National Science Foundation Graduate Research Fellowship. J.R.H. and Y.S. were funded by the Wilke Family Foundation, Merck and the Biomedical Advanced Research and Development Authority under Contract HHSO10201600031C. L.M.S. and M.J.S. were supported by NIAID under award number R01AI139550. C.L.D. received funding from NIAID under award numbers U19-AI057229 and U19-AI090019. C.K. was funded by NIH R01DK063158. K.C.N. was funded by the Sunshine Foundation and the Sean N Parker Center at Stanford University. N.S. was supported by a Postdoctoral Fellowship from National Multiple Sclerosis Society (NMSS) and also a Career Transition Grant from NMSS. S.L.H. is supported by the NIH/NINDS (R35NS111644) and the Valhalla Foundation. P.J.U. was supported by National Institute of Allergy and Infectious Diseases of the National Institutes of Health, R01 AI125197-04 and the Henry Gustav Floren Trust. M.M.D. and L.M.S. were supported by the NIH (R01AI139550). **Author contributions:** J.L., N.S., and M.M.D conceived the study. J.L. and N.S. performed the experiments. M.Z., J.L. S.H.C., J.P., and A.T.S. analyzed the single-cell RNA-seq data. M.J.S., L.C., and L.M.S. contributed to analysis of bulk RNA-seq data. X.J. prepared the bulk RNA-seq libraries. A.C. and L.M.S. provided the HLA-DQ2.5 molecules. J.L. prepared samples for bulk TCRβ sequencing. J.W. and A.M.M. performed single cell TCR-seq. Y.S. and J.R.H. generated the single-cell RNA-seq data of COVID-19 patients. V.V.A.M. prepared the HLA-DR4 molecules. B.A.P. and C.K. provided deamidated gluten. L.B.K., J.E.D., S.L.H., and J.R.O. provided PBMCs from MS patients. K.B., W.H.R., and P.J.U. provided PBMCs from SLE patients. K.C.N. and N.Q.F. provided blood and biopsy samples from CeD patients. K.C.N. and G.K.R.D. provided PBMCs and clinical metadata of COVID-19 patients. C.L.D. provided PBMCs from influenza-infected patients. J.L. and M.M.D. wrote the manuscript with helpful input from all the authors. **Competing interests:** N.S., M.M.D., and J.L. are co-inventors on patent application 62/882,810, which includes discoveries described in this manuscript. S.L.H. serves on the board of directors for Neurona and on scientific advisory boards for Accure, Alector, Annexon, and Molecular Stethoscope; and has received travel reimbursement and writing assistance from F. Hoffmann-La Roche and Novartis for CD20-related meetings and presentations. M.M.D. is involved in a start-up company, Mozart Therapeutics, Inc., for the clinical applications of these discoveries. L.M.S. has served as consultant for Mozart Therapeutics, Inc. J.D. has received honoraria for consulting services within the last three years from Alexion, Biogen, Bristol Myers Squibb, Celgene, Genentech, Genzyme, Janssen Pharmaceuticals, Novartis. J.H. is a PACT Phrama Board Member for Isoplexis Corp and on the board for Research Funding from Merck and Gilead. Other co-authors declare that they have no competing interests. **Data and materials availability:** Raw and processed RNA-seq data that support the findings of this study have been deposited in the Gene Expression Omnibus under the accession number GSE193442. All custom-made code can be accessed at https://github.com/davis-lab-stanford/kir-cd8 and has been deposited into a Zenodo record (*56*). Biotinylated HLA-DQ molecules is available under a material transfer agreement with the University of Oslo from Ludvig M. Sollid. All other data are available in the main text or the supplementary materials. This work is licensed under a Creative Commons Attribution 4.0 International (CC BY 4.0) license, which permits unrestricted use, distribution, and reproduction in any medium, provided the original work is properly cited. To view a copy of this license, visit https://creativecommons.org/licenses/by/4.0/. This license does not apply to figures/photos/artwork or other content included in the article that is credited to a third party; obtain authorization from the rights holder before using such material.

SUPPLEMENTARY MATERIALS
science.org/doi/10.1126/science.abi9591
Figs. S1 to S11
Tables S1 to S7
MDAR Reproducibility Checklist

12 April 2021; resubmitted 12 October 2021
Accepted 1 March 2022
Published online 8 March 2022
10.1126/science.abi9591

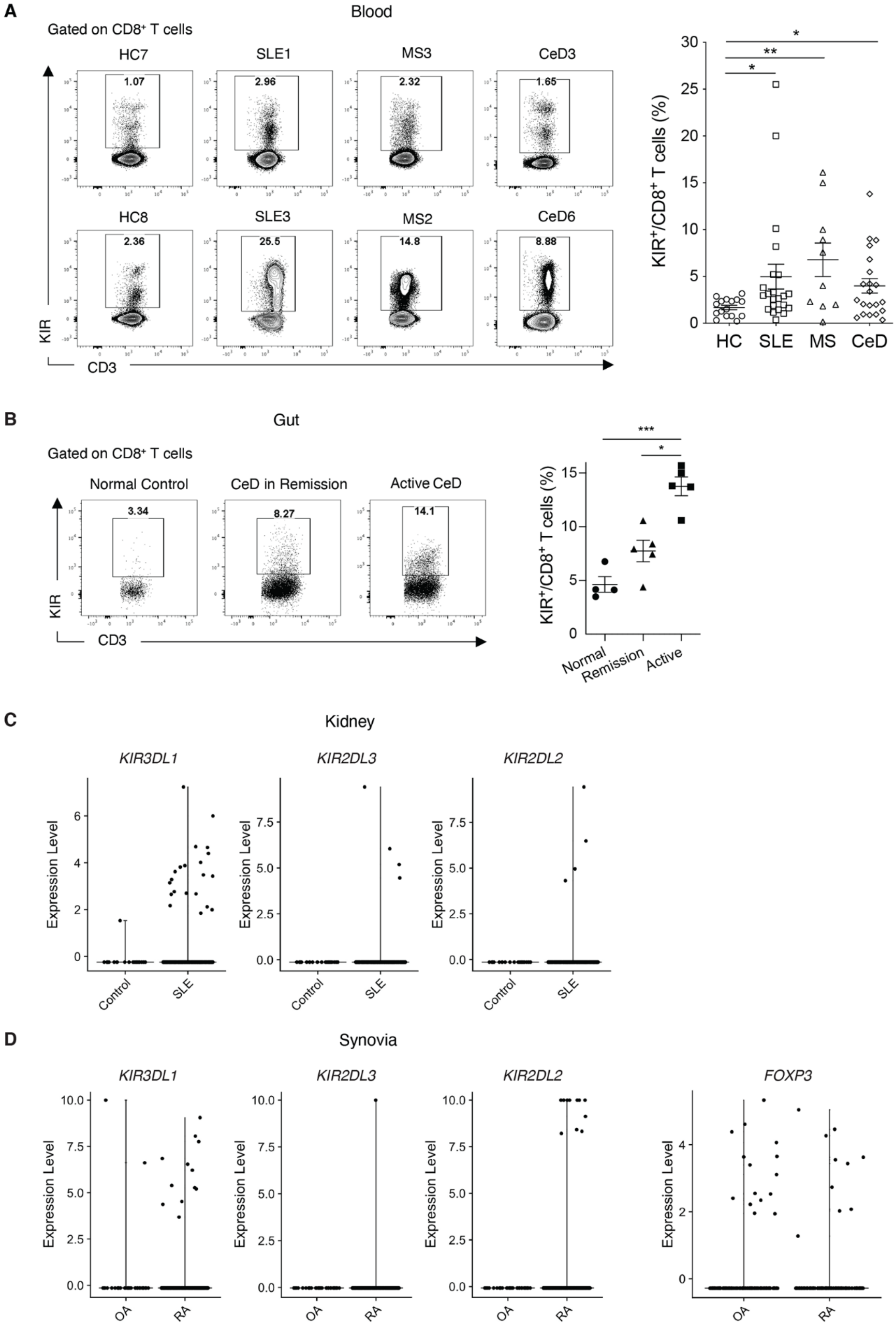
A
Blood
Gated on CD8⁺ T cells
HC7
SLE1
MS3
CeD3
1.07
2.96
2.32
1.65
HC8
SLE3
MS2
CeD6
2.36
25.5
14.8
8.88
KIR
CD3
KIR⁺/CD8⁺ T cells (%)
HC
SLE
MS
CeD
B
Gut
Gated on CD8⁺ T cells
Normal Control
CeD in Remission
Active CeD
3.34
8.27
14.1
KIR
CD3
KIR⁺/CD8⁺ T cells (%)
Normal
Remission
Active
C
Kidney
KIR3DL1
KIR2DL3
KIR2DL2
Expression Level
Control
SLE
D
Synovia
KIR3DL1
KIR2DL3
KIR2DL2
FOXP3
Expression Level
OA
RA

Fig. 1. Increased KIR$^+$CD8$^+$ T cells in patients with autoimmune diseases. (**A**) Representative contour plots and a summary histogram showing the frequency of KIR$^+$CD8$^+$ T cells in the peripheral blood of healthy controls (HC, N=16) and patients with systemic lupus erythematous (SLE, N=22), multiple sclerosis (MS, N=10) or celiac disease (CeD, N=21) analyzed by flow cytometry. KIR$^+$ cells were detected by PE-conjugated antibodies against KIR2DL1 (clone#143211), KIR2DL2/L3 (Dx27), KIR2DL5 (UP-R1), KIR3DL1 (Dx9), and KIR3DL2 (clone#539304). *P<0.05, **P<0.01, Kruskal–Wallis test followed by multiple comparisons test. (**B**) Representative contour plots and a summary histogram showing the frequency of KIR$^+$CD8$^+$ T cells in the duodenum of normal controls (N=4), CeD in remission (N=5), and active CeD (N=5) analyzed by flow cytometry. *P<0.05, ***P<0.001, Kruskal–Wallis test followed by multiple comparisons test. (**C**) Expression of *KIR* transcripts (*KIR3DL1*, *KIR2DL3* and *KIR2DL2*) in CD8$^+$ T cells from healthy kidneys (control, N=6) versus SLE nephritis kidneys (N=20) is shown. (**D**) Expression of *KIR* transcripts (*KIR3DL1*, *KIR2DL3* and *KIR2DL2*) in synovial CD8$^+$ T cells and expression of *FOXP3* in synovial CD4$^+$ T cells from rheumatoid arthritis (RA, N=18) and osteoarthritis (OA, N=3) are shown.

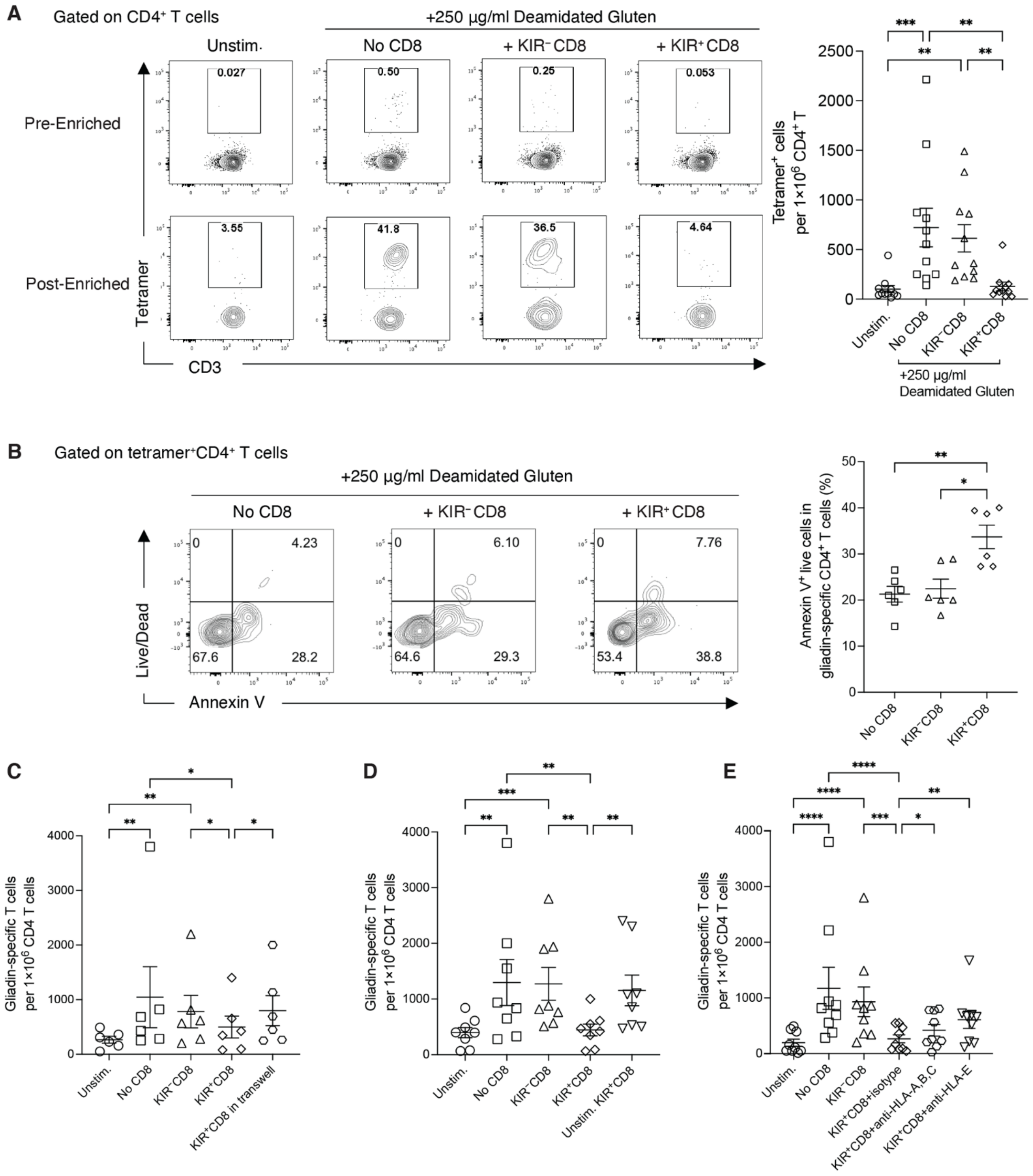
A
Gated on CD4+ T cells
+250 μg/ml Deamidated Gluten
Unstim.
No CD8
+ KIR− CD8
+ KIR+ CD8
Pre-Enriched
Post-Enriched
Tetramer
CD3
0.027
0.50
0.25
0.053
3.55
41.8
36.5
4.64
Tetramer+ cells per 1×10^6 CD4+ T
Unstim.
No CD8
KIR−CD8
KIR+CD8
+250 μg/ml Deamidated Gluten
B
Gated on tetramer+CD4+ T cells
+250 μg/ml Deamidated Gluten
No CD8
+ KIR− CD8
+ KIR+ CD8
Live/Dead
Annexin V
67.6
28.2
4.23
64.6
29.3
6.10
53.4
38.8
7.76
Annexin V+ live cells in gladin-specific CD4+ T cells (%)
C
D
E
Gliadin-specific T cells per 1×10^6 CD4 T cells
Unstim.
No CD8
KIR−CD8
KIR+CD8
KIR+CD8 in transwell
Unstim. KIR+CD8
KIR+CD8+isotype
KIR+CD8+anti-HLA-A,B,C
KIR+CD8+anti-HLA-E

Fig. 2. Elimination of gliadin-specific CD4⁺ T cells by KIR⁺CD8⁺ T cells. (**A**) Representative contour plots showing tetramer bound $CD4^+$ T cells after enrichment and summary of the number of gliadin-specific $CD4^+$ T cells per 1×10^6 $CD4^+$ T cells on Day 6. Experiments were repeated using PBMCs from 11 CeD patients. $^{**}P<0.01$, $^{***}P<0.001$, Friedman test followed by multiple comparisons test. (**B**) Representative contour plots and a summary graph displaying Annexin V binding of gliadin-specific $CD4^+$ T cells from the culture harvested on Day 3 (N=6). $^{*}P<0.05$, $^{**}P<0.01$, Friedman test followed by multiple comparisons test. (**C**) Frequency of gliadin-specific $CD4^+$ T cells from the cell cultures in the presence or absence of KIR^- or KIR^+CD8^+ T cells or with KIR^+CD8^+ T cells separated by a 4-μm insert in a transwell plate (N=6). $^{*}P<0.05$, $^{**}P<0.01$, Friedman test followed by multiple comparisons test. (**D**) Frequency of gliadin-specific $CD4^+$ T cells from the PBMC cultures in the presence or absence of KIR^- or KIR^+CD8^+ T cells with or without pre-activation (N=8). $^{**}P<0.01$, $^{***}P<0.001$, Friedman test followed by multiple comparisons test. (**E**) Frequency of gliadin-specific $CD4^+$ T cells from the PBMC cultures in the presence or absence of pre-activated KIR^- or KIR^+ $CD8^+$ T cells with isotype control, anti-HLA-ABC, or anti-HLA-E blockade (N=9). $^{*}P<0.05$, $^{**}P<0.01$, $^{***}P<0.001$, $^{****}P<0.0001$, Friedman test followed by multiple comparisons test.

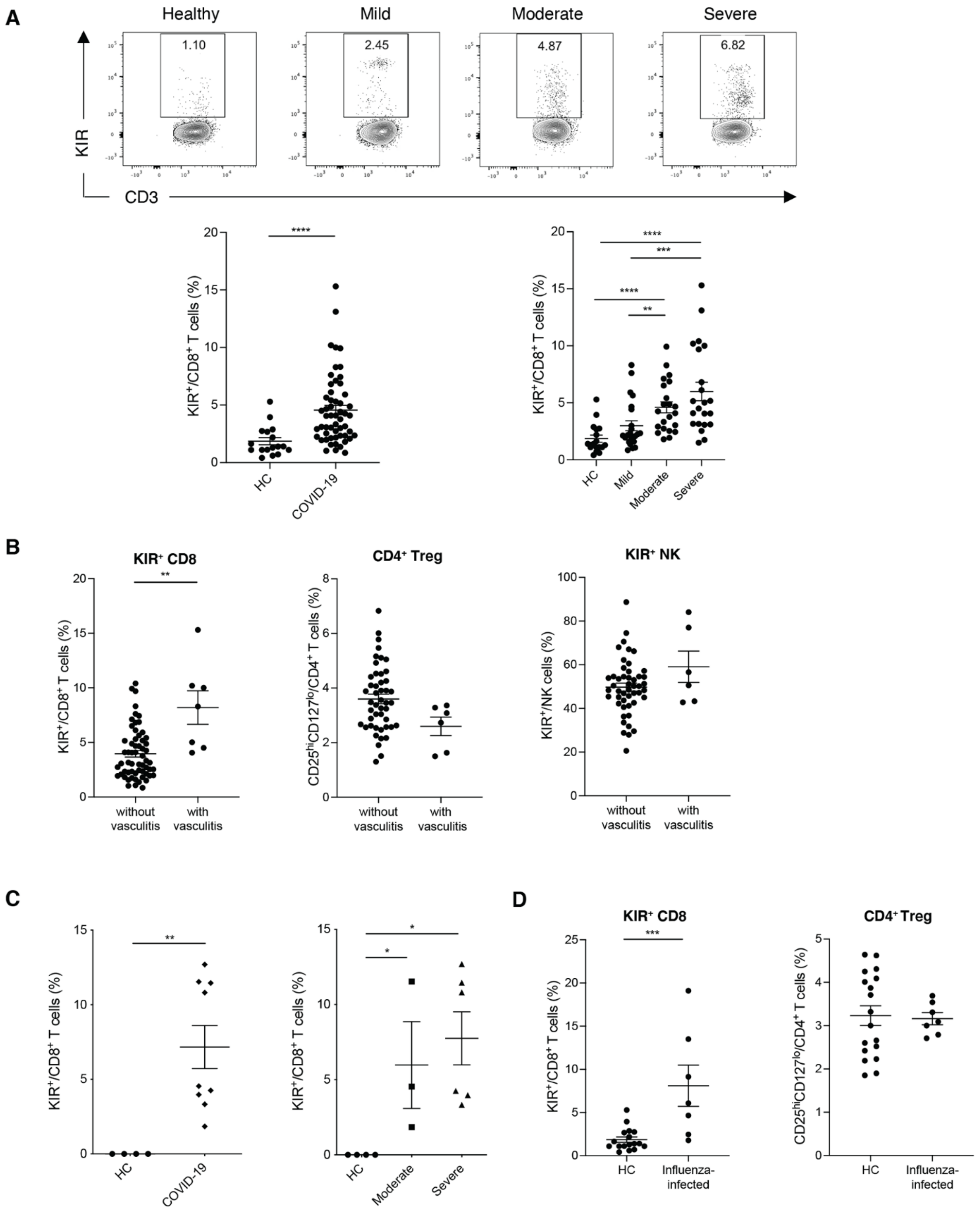
A
Healthy
Mild
Moderate
Severe
1.10
2.45
4.87
6.82
KIR
CD3
KIR+/CD8+ T cells (%)
HC
COVID-19
Mild
Moderate
Severe
B
KIR+ CD8
CD4+ Treg
KIR+ NK
CD25hiCD127lo/CD4+ T cells (%)
KIR+/NK cells (%)
without vasculitis
with vasculitis
C
D
Influenza-infected

Fig. 3. Increased KIR$^+$CD8$^+$ T cells in infectious diseases. (**A**) Representative contour plots and summary scatter plots showing the percentage of KIR$^+$ cells in CD8$^+$ T cells from the blood of 17 healthy controls and 53 COVID-19 patients with varying disease severity (mild: N=23, moderate: N=17, severe: N=13). left: ****$P<0.0001$, Mann–Whitney test; right: **$P<0.01$, ***$P<0.001$, ****$P<0.0001$, Kruskal–Wallis test followed by multiple comparisons test. (**B**) Frequency of KIR$^+$CD8$^+$ T cells, CD4$^+$ Tregs (CD25^{hi}CD127^{lo}), and KIR$^+$ NK cells in the blood of COVID-19 patients with or without vasculitis. **$P<0.01$, Mann–Whitney test. (**C**) Frequency of CD8$^+$ T cells expressing *KIR* transcripts (*KIR3DL1*, *KIR3DL2*, *KIR2DL3*, or *KIR2DL1*) in the bronchoalveolar lavage fluid of healthy controls (N=4) versus COVID-19 patients (N=9) (left, **$P<0.01$, Mann–Whitney test) and healthy controls versus COVID-19 patients with moderate (N=3) or severe (N=6) disease (right, *$P<0.05$, Kruskal–Wallis test followed by multiple comparisons test). (**D**) Frequency of KIR$^+$CD8$^+$ T cells and CD4$^+$ Tregs (CD25^{hi}CD127^{low}) in the blood of healthy controls (N=17) versus influenza-infected patients (N=7). **$P<0.01$, Mann–Whitney test.

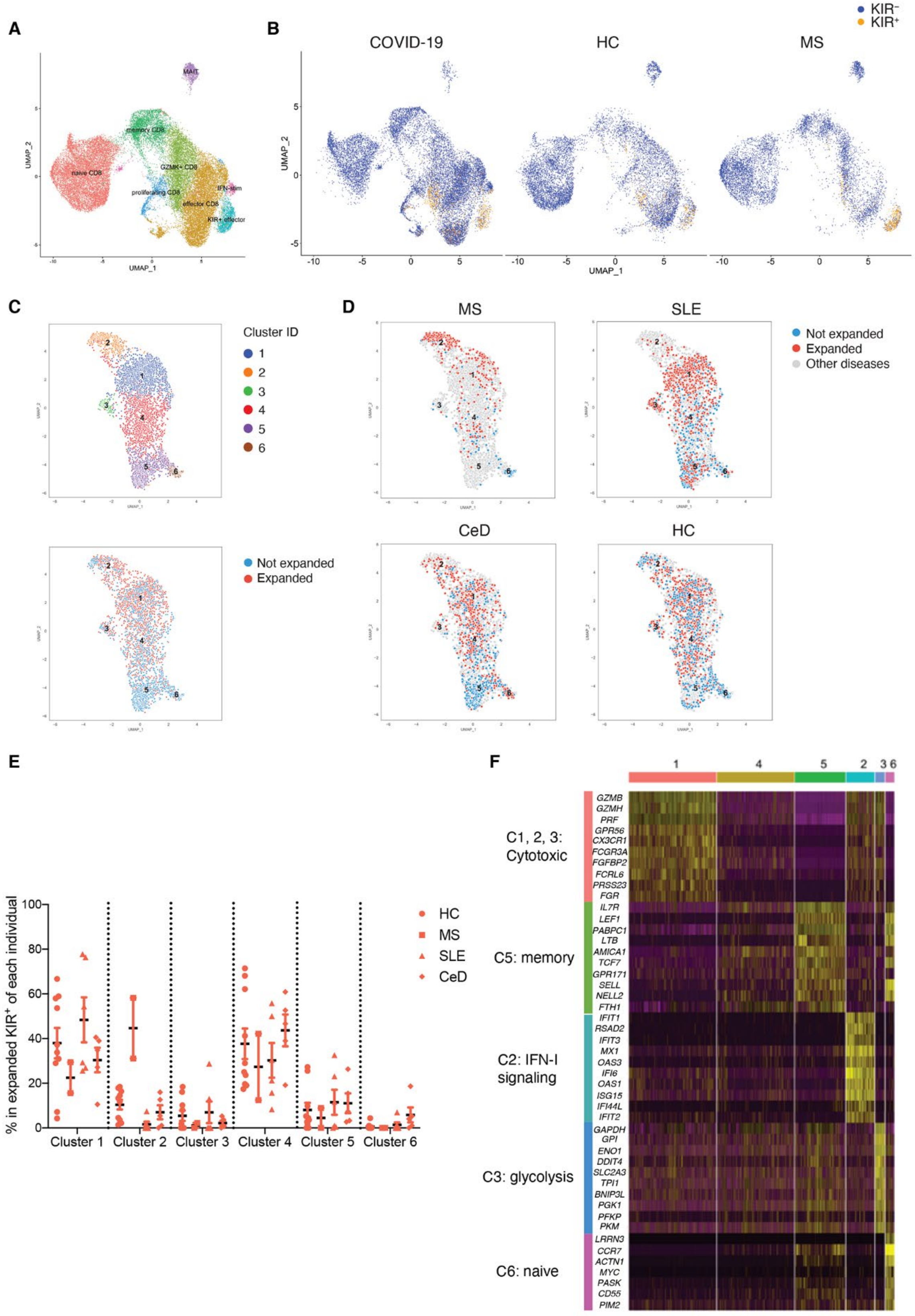
A
B
C
D
E
F
COVID-19
HC
MS
KIR⁻
KIR⁺
UMAP_1
UMAP_2
MAIT
memory CD8
naive CD8
GZMK+ CD8
IFN-stim
proliferating CD8
effector CD8
KIR+ effector
Cluster ID
Not expanded
Expanded
Other diseases
SLE
CeD
% in expanded KIR⁺ of each individual
Cluster 1
Cluster 2
Cluster 3
Cluster 4
Cluster 5
Cluster 6
C1, 2, 3: Cytotoxic
C5: memory
C2: IFN-I signaling
C3: glycolysis
C6: naive
GZMB
GZMH
PRF
GPR56
CX3CR1
FCGR3A
FGFBP2
FCRL6
PRSS23
FGR
IL7R
LEF1
PABPC1
LTB
AMICA1
TCF7
GPR171
SELL
NELL2
FTH1
IFIT1
RSAD2
IFIT3
MX1
OAS3
IFI6
OAS1
ISG15
IFI44L
IFIT2
GAPDH
GPI
ENO1
DDIT4
SLC2A3
TPI1
BNIP3L
PGK1
PFKP
PKM
LRRN3
CCR7
ACTN1
MYC
PASK
CD55
PIM2

Fig. 4. Single-cell RNA-seq analysis of KIR⁺CD8⁺ T cells in the blood. (**A** and **B**) Single-cell RNA-seq analysis of total CD8⁺ T cells from the blood of healthy subjects (N=10), MS patients (N=6), and COVID-19 patients (N=25) by 10X Genomics. (**A**) UMAP plot of the eight subpopulations identified by unsupervised clustering. (**B**) UMAP plots showing the distribution of KIR⁺CD8⁺ T cells (expressing *KIR3DL1*, *KIR3DL2*, *KIR2DL1*, or *KIR2DL3* transcripts) and KIR⁻CD8⁺ T cells from healthy controls (HC), MS patients and COVID-19 patients. (**C** to **F**) KIR⁺CD8⁺ T cells in the blood of HC (N=10) and patients with MS (N=2), SLE (N=6), and CeD (N=5) were sorted for single-cell RNA-seq using the Smart-seq2 protocol and analyzed using the R package 'Seurat'. (**C**) UMAP plots showing KIR⁺CD8⁺ T cells segregated into six clusters (upper) and the distribution of expanded (≥2 cells expressing same TCR) and unexpanded (cell expressing unique TCR) cells (lower). (**D**) UMAP plots of KIR⁺CD8⁺ T cells from MS, SLE, CeD, and HC are shown, with expanded and unexpanded cells annotated with different colors (expanded: red; unexpanded: blue; other diseases: gray). (**E**) Cluster compositions of expanded KIR⁺CD8⁺ T cells from each individual. (**F**) Heatmap showing expression of the top-10 genes differentially expressed in each cluster, with the categories of each group of genes annotated on the left.

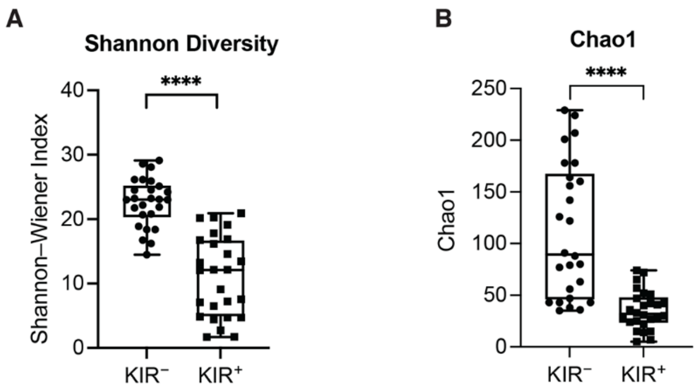

Fig. 5. Analysis of T cell receptor sequences of KIR⁺CD8⁺ T cells. (**A** and **B**) Summary histograms showing Shannon–Wiener Index (A) and Chao estimates (B) of TCRs of KIR⁻ versus KIR⁺ CD8⁺ T cells from 26 subjects including 11 healthy donors, two MS, five SLE, three CeD, and five T1D patients as evaluated by VDJtools. ****P<0.0001, Wilcoxon matched-pairs signed-rank test.

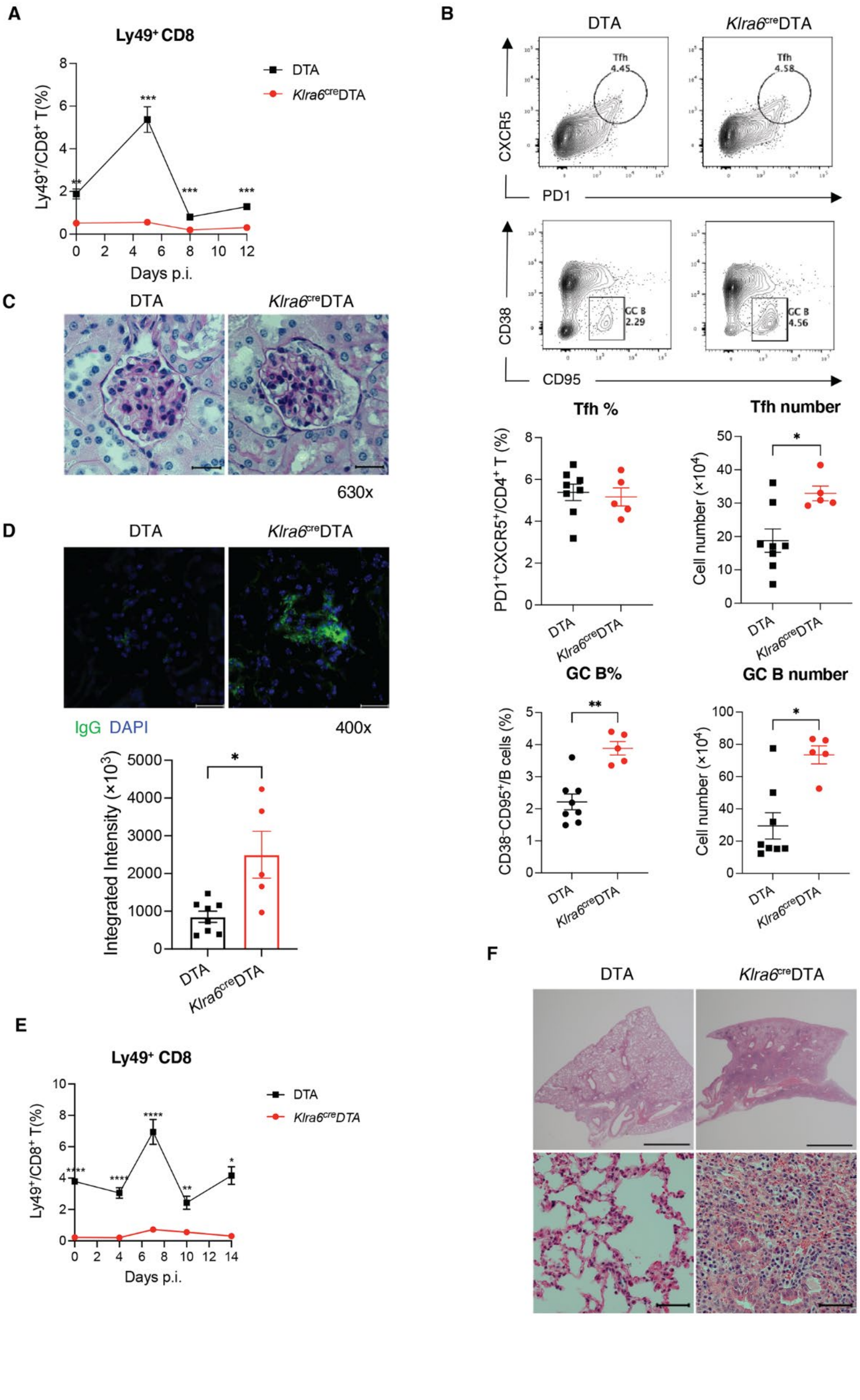
A
Ly49+ CD8
Ly49+/CD8+ T (%)
Days p.i.
DTA
Klra6creDTA
B
DTA
Klra6creDTA
CXCR5
PD1
Tfh
4.45
4.58
CD38
CD95
GC B
2.29
4.56
Tfh %
Tfh number
PD1+CXCR5+/CD4+ T (%)
Cell number (×10^4)
GC B%
GC B number
CD38−CD95+/B cells (%)
C
630x
D
IgG DAPI
400x
Integrated Intensity (×10^3)
E
F

Fig. 6. Exacerbated autoimmunity in *Klra6*[cre]DTA mice after viral infection. (**A**) Frequency of $Ly49^+CD8^+$ T cells in the blood of DTA mice (N=8) and *Klra6*[cre]DTA mice (N=5) 0, 5, 8, and 12 days post LCMV-Armstrong infection. **$P<0.01$, ***$P<0.001$, RM two-way ANOVA followed by multiple comparisons test. (**B**) Representative contour plots and summarized scatter plots displaying frequency and absolute number of $PD1^+CXCR5^+CD4^+$ T cells (Tfh) and $CD38^-CD95^+$ germinal center (GC) B cells in the spleen of DTA mice (N=8) and *Klra6*[cre]DTA mice (N=5) 30 days after LCMV-Armstrong infection. *$P<0.05$, **$P<0.01$, Mann–Whitney test. (**C**) Representative kidney pathology of day 30 LCMV-infected DTA mice and *Klra6*[cre]DTA mice assessed by PAS staining (630X, scale bar: 20 μm). (**D**) IgG deposition in glomeruli of the kidneys of DTA mice (N=8) and *Klra6*[cre]DTA mice (N=5) 30 days post LCMV infection accessed by immunofluorescence staining (400X, scale bar: 20 μm) and quantified by ImageJ. *$P<0.05$, Mann–Whitney test. (**E**) Frequency of $Ly49^+CD8^+$ T cells in the blood of DTA mice (N=6) and *Klra6*[cre]DTA mice (N=5) after influenza A/PR/8/34 H1N1 infection. *$P<0.05$, **$P<0.01$, ****$P<0.0001$, RM two-way ANOVA followed by multiple comparisons test. (**F**) Microscopy of representative hematoxylin and eosin staining of lung sections from DTA mice and *Klra6*[cre]DTA mice 60 days post influenza infection (upper panel: 20X, scale bar, 2 mm; lower panel: 630X, scale bar, 50 μm). Representative data from two independent experiments are shown. The mean ± SEM is indicated.

Table 1. Selected specificity groups from the GLIPH2 analysis that contained TCRβ sequences from three or more individuals and exhibited significant bias of V-gene usage (*P*<0.05). Significant motif residues are highlighted in red in CDR3 alignments.

Group ID	Subject: Condition	CDR3β	TRBV	TRBJ	%	HLA-A	HLA-B	HLA-C
Group I motif-PFGE	CD229:CeD	CASGTGPFGELFF	TRBV10-2	TRBJ2-2	78.801	A*26:01/A*32:01	B*27:02/B*38:01!	C*02:02/C*12:03
	HC1505:HC	CASGTGPFGELFF	TRBV10-2	TRBJ2-2	54.545	A*11:01/A*34:01	B*38:02!/B*40:02	C*07:02/C*15:02!
	OHC018:COVID-19	CASSPFGETQYF	TRBV11-2	TRBJ2-5	0.427	A*24:02/A*29:02	B*08:01/B*51:01	C*07:01/C*15:02!
	0033:COVID-19	CASSPFGETQYF	TRBV25-1	TRBJ2-5	14.67	A*02:01/A*03:01	B*07:02/B*49:01	C*07:01/C*07:02
Group II global-S%DSGGTDT	T1D25:T1D	CASSFDSGGTDTQYF	TRBV7-2	TRBJ2-3	3.053	A*03:01!/A*03:01!	B*40:01/B*49:01!	C*03:04/C*07:01
	OHC015:COVID-19	CASSFDSGGTDTQYF	TRBV7-3	TRBJ2-3	0.629	A*02:01/A*11:02	B*27:04/B*35:01	C*03:03/C*12:02
	0033:COVID-19	CASSLDSGGTDTQYF	TRBV7-2	TRBJ2-3	0.016	A*02:01/A*03:01!	B*07:02/B*49:01!	C*07:01/C*07:02
Group III global-%EGLAGLQET	BBS660:SLE	CASSEGLAGLQETQYF	TRBV27	TRBJ2-5	7.286	A*24:02/A*25:01	B*08:01!/B*15:01	C*03:03/C*07:01
	CD224:CeD	CASSEGLAGLQETQYF	TRBV27	TRBJ2-5	0.541	A*01:01/A*02:01	B*08:01!/B*08:01!	C*07:01/C*07:01
	BBS660:SLE_KIR-	CASSEGLAGLQETQYF	TRBV27	TRBJ2-5	1.136	A*24:02/A*25:01	B*08:01!/B*15:01	C*03:03/C*07:01
	T1D51:T1D	CASGEGLAGLQETQYF	TRBV27	TRBJ2-5	0.313	A*02:01/A*23:17	B*08:01!/B*40:02	C*04:01/C*18:01
Group IV motif-SQDF	0033:COVID-19	CASSQDFWGRYEQYF	TRBV4-3	TRBJ2-7	0.016	A*02:01/A*03:01!	B*07:02/B*49:01	C*07:01/C*07:02
	KBSLE20:SLE	CASSQDFFNEQFF	TRBV4-1	TRBJ2-1	0.204	A*02:06/A*30:01	B*13:02!/B*15:25	C*04:03/C*06:02
	0033:COVID-19	CASSQDFEGGYEQYF	TRBV4-2	TRBJ2-7	0.016	A*02:01/A*03:01!	B*07:02/B*49:01	C*07:01/C*07:02
	HC0231:HC	CASSQDFRGLSSRSYNEQFF	TRBV3-1	TRBJ2-1	0.004	A*03:01!/A*03:01!	B*35:03/B*52:01	C*12:02/C*12:03
	KBSLE20:SLE	CASSQDFLNEQFF	TRBV4-1	TRBJ2-1	0.815	A*02:06/A*30:01	B*13:02!/B*15:25	C*04:03/C*06:02
	KBSLE20:SLE	CASSQDFYNEQFF	TRBV4-1	TRBJ2-1	0.407	A*02:06/A*30:01	B*13:02!/B*15:25	C*04:03/C*06:02
	HC4084:HC	CASSQDFGGIYGYTF	TRBV14-1	TRBJ1-2	0.061	A*03:02!/A*11:01!	B*35:01/B*35:03	C*04:01/C*04:01
	HC2061:HC_KIR-	CASSQDFSRPYGYTF	TRBV4-3	TRBJ1-2	2.439	A*11:01!/A*11:01!	B*27:05/B*55:01	C*01:02/C*03:03
	KBSLE20:SLE	CASSQDFRNEQYF	TRBV4-1	TRBJ2-7	0.407	A*02:06/A*30:01	B*13:02!/B*15:25	C*04:03/C*06:02
	KBSLE20:SLE	CASSQDFHNEQFF	TRBV4-1	TRBJ2-1	0.204	A*02:06/A*30:01	B*13:02!/B*15:25	C*04:03/C*06:02
	KBSLE20:SLE	CASSQDFGGEQFF	TRBV4-1	TRBJ2-1	0.407	A*02:06/A*30:01	B*13:02!/B*15:25	C*04:03/C*06:02
	KBSLE07:SLE	CASSQDFRGGNTEAFF	TRBV3-1	TRBJ1-1	0.214	A*02:03/A*11:01!	B*13:01!/B*18:01	C*03:04/C*07:04
	HC0230:HC	CASSQDFPGQEETQYF	TRBV4-2	TRBJ2-5	0.787	A*02:06/A*33:03	B*13:01!/B*58:01	C*03:02/C*03:04
	KBSLE20:SLE	CASSQDFNNEQFF	TRBV4-1	TRBJ2-1	1.629	A*02:06/A*30:01	B*13:02!/B*15:25	C*04:03/C*06:02
	HC1505:HC	CASSQDFLLQGDLEEQYF	TRBV3-1	TRBJ2-7	0.023	A*11:01!/A*34:01	B*38:02/B*40:02	C*07:02/C*15:02
	HC0231:HC	CASSQDFSYNSPLHF	TRBV3-1	TRBJ1-6	0.011	A*03:01!/A*03:01!	B*35:03/B*52:01	C*12:02/C*12:03
Group V motif-PFF	KBSLE22:SLE	CASSPFFGDTEAFF	TRBV6-5	TRBJ1-1	0.84	A*33:03!/A*66:02	B*44:03!/B*58:01!	C*03:02/C*07:01
	BBS706:SLE	CASSPFFPGGPYEQYF	TRBV6-5	TRBJ2-7	22.609	A*02:01/A*02:01	B*44:02!/B*44:02!	C*05:01/C*05:01
	BBS706:SLE	CASSPFFPGDPYEQYF	TRBV6-5	TRBJ2-7	0.29	A*02:01/A*02:01	B*44:02!/B*44:02!	C*05:01/C*05:01
	HC0230:HC	CASSFPFFAEAFF	TRBV11-3	TRBJ1-1	0.11	A*02:06/A*33:03!	B*13:01/B*58:01!	C*03:02/C*03:04
Group IV motif-SQEI	OHC014:COVID-19	CASSQEIRGWDTEAFF	TRBV3-1	TRBJ1-1	0.011	A*11:01/A*23:01	B*35:02/B*49:01!	C*04:01/C*07:01
	HC1505:HC	CASSQEIMTAYGYTF	TRBV4-1	TRBJ1-2	0.023	A*11:01/A*34:01	B*38:02/B*40:02	C*07:02/C*15:02
	BBS706:SLE_KIR-	CASSQEIAGSSYNEQFF	TRBV3-1	TRBJ2-1	0.242	A*02:01/A*02:01	B*44:02/B*44:02	C*05:01!/C*05:01!
	0033:COVID-19	CASSQEISQPQHF	TRBV4-1	TRBJ1-5	0.016	A*02:01/A*03:01	B*07:02/B*49:01!	C*07:01/C*07:02
	HC2419:HC	CASSQEIGRATDTQYF	TRBV4-2	TRBJ2-3	0.215	A*02:06/A*24:02	B*40:01/B*48:01	C*08:01/C*12:03
	KBSLE18:SLE	CASSQEIPITGNTIYF	TRBV4-1	TRBJ1-3	0.238	A*30:01/A*32:01	B*15:01/B*44:02	C*04:01/C*05:01!
	HC2164:HC	CASSQEIAGGETEAFF	TRBV4-2	TRBJ1-1	0.006	A*02:01/A*24:02	B*15:13/B*44:02	C*05:01!/C*08:01
	0033:COVID-19	CASSQEIGVVETQYF	TRBV3-1	TRBJ2-5	0.423	A*02:01/A*03:01	B*07:02/B*49:01!	C*07:01/C*07:02
	HC0231:HC	CASSQEIGQLNQPQHF	TRBV4-1	TRBJ1-5	0.004	A*03:01/A*03:01	B*35:03/B*52:01	C*12:02/C*12:03
