# Supplementary material for: KIR+CD8+ T cells suppress pathogenic T cells and are active in autoimmune diseases and COVID-19
Source: Science. 2022 Mar 8;376(6590):eabi9591. doi: 10.1126/science.abi9591 (PMC8995031; doi:10.1126/science.abi9591)
Supplement: Supplementary file 2 — Figs. S1 to S11 [file science.abi9591_sm.pdf]

Science AAAS

Supplementary Materials for

KIR^{+}CD8^{+} T cells suppress pathogenic T cells and are active in autoimmune diseases and COVID-19

Jing Li *et al*.

Corresponding authors: Naresha Saligrama, nareshas@wustl.edu; Mark M. Davis, mmdavis@stanford.edu

Science **376**, eabi9591 (2022)
DOI: 10.1126/science.abi9591

The PDF file includes:

Figs. S1 to S11

Other Supplementary Material for this manuscript includes the following:

Tables S1 to S7
MDAR Reproducibility Checklist

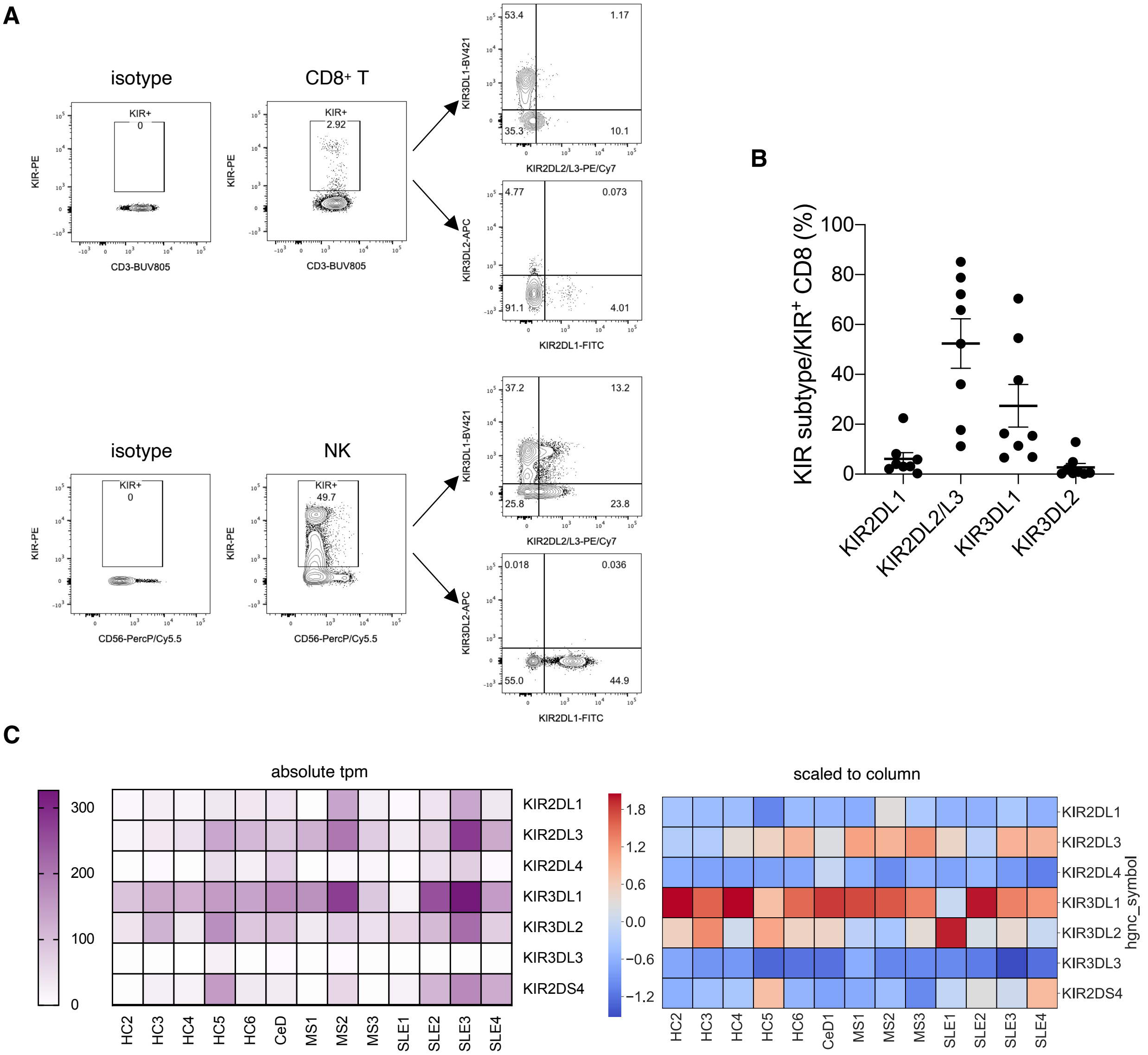

Fig. S1. KIR3DL1 and KIR2DL3 are the two major KIR subtypes expressed on CD8$^+$ T cells. (A) Representative plots showing expression of KIR subtypes among KIR$^+$CD8$^+$ T cells and KIR$^+$ NK cells in the peripheral blood, detected by flow cytometry. Total PBMCs were stained with PE-conjugated antibodies against KIR2DL1/S5, KIR2DL2/L3/S2, KIR2DL5, KIR3DL1, KIR3DL2 and FITC-conjugated anti-KIR2DL1/S5, PE/Cy7-conjugated anti-KIR2DL2/L3/S2, BV421-conjugated anti-KIR3DL1, APC-conjugated anti-KIR3DL2, together with other surface antibodies. (**B**) A summary scatter plot showing the percentage of KIR2DL1$^+$, KIR2DL2/L3$^+$, KIR3DL1$^+$ and KIR3DL2$^+$ cells among KIR$^+$CD8$^+$ T cells from the blood of healthy controls (N=8). (**C**) Transcript levels of different KIR genes in sorted KIR$^+$CD8$^+$ T cells detected by RNA sequencing. Left panel shows absolute tpm (transcript per million reads) of the KIR genes in each individual, while right panel shows normalized expression level of the KIR genes (scaled to column) among different donors.

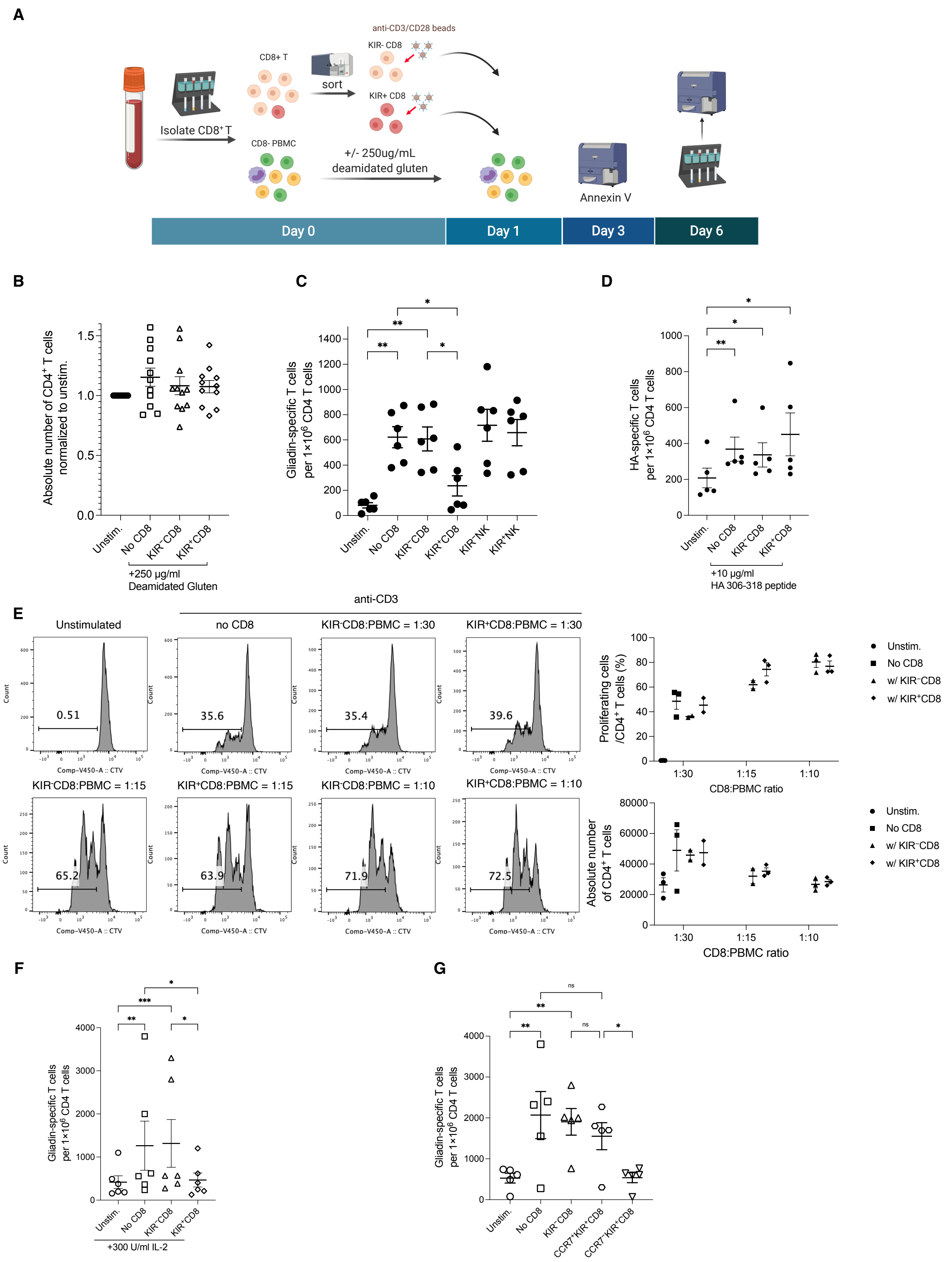
A
anti-CD3/CD28 beads
CD8+ T
KIR- CD8
KIR+ CD8
sort
Isolate CD8⁺ T
CD8- PBMC
+/- 250ug/mL deamidated gluten
Annexin V
Day 0
Day 1
Day 3
Day 6
B
Absolute number of CD4⁺ T cells normalized to unstim.
Unstim.
No CD8
KIR⁻CD8
KIR⁺CD8
+250 µg/ml Deamidated Gluten
C
Gliadin-specific T cells per 1×10⁶ CD4 T cells
KIR⁻NK
KIR⁺NK
D
HA-specific T cells per 1×10⁶ CD4 T cells
+10 µg/ml HA 306-318 peptide
E
anti-CD3
Unstimulated
no CD8
KIR⁻CD8:PBMC = 1:30
KIR⁺CD8:PBMC = 1:30
KIR⁻CD8:PBMC = 1:15
KIR⁺CD8:PBMC = 1:15
KIR⁻CD8:PBMC = 1:10
KIR⁺CD8:PBMC = 1:10
Count
Comp-V450-A :: CTV
0.51
35.6
35.4
39.6
65.2
63.9
71.9
72.5
Proliferating cells /CD4⁺ T cells (%)
Absolute number of CD4⁺ T cells
CD8:PBMC ratio
Unstim.
No CD8
w/ KIR⁻CD8
w/ KIR⁺CD8
F
Gliadin-specific T cells per 1×10⁶ CD4 T cells
+300 U/ml IL-2
G
CCR7⁺KIR⁺CD8
CCR7⁻KIR⁺CD8
ns

Fig. S2. KIR^+CD8^+ T cells target gliadin-specific pathogenic $CD4^+$ T cells specifically. (**A**) Experimental schematic. (**B**) The absolute number of total $CD4^+$ T cells in the cultures of Day 6 (N=11) as described in Fig. 3A. (**C**) Frequency of gliadin-specific $CD4^+$ T cells in the co-cultures of sorted KIR^-CD8^+ T cells, KIR^+CD8^+ T cells, KIR^- NK or KIR^+ NK with $CD8^-$ PBMCs from CeD patients (N=6) on Day 6 in responses to stimulation with 250 μg/ml of deamidated gluten. **P*<0.05, ***P*<0.01, Friedman test followed by multiple comparisons test. (**D**) Frequency of HA-specific $CD4^+$ T cells (detected by DRB1*04: Influenza A HA 306-318 tetramers) in the co-cultures of pre-activated KIR^- or KIR^+ $CD8^+$ T cells with $CD8^-$ PBMCs from HLA-$DR4^+$ healthy donors (N=5) on Day 6 in responses to stimulation with 10 μg/ml of HA 306-318 peptides. **P*<0.05, ***P*<0.01, Friedman test followed by multiple comparisons test. (**E**) Representative figures and summary histograms showing the proliferation and absolute numbers of $CD4^+$ T cells without stimulation or cultured with/without pre-activated KIR^- or KIR^+ $CD8^+$ T cells at different $CD8^+$ T: $CD8^-$PBMC ratios in the presence of plate-bound anti-CD3. (**F**) Frequency of gliadin-specific $CD4^+$ T cells in the co-cultures with sorted KIR^- or KIR^+CD8^+ T cells from CeD patients (N=6) supplemented with 300 U/ml of IL-2 upon gliadin stimulation. **P*<0.05, ***P*<0.01, ***P*<0.001, Friedman test followed by multiple comparisons test. (**G**) Frequency of gliadin-specific $CD4^+$ T cells co-cultured with sorted KIR^-CD8^+ T cells, $CCR7^+KIR^+CD8^+$ T cells or $CCR7^-KIR^+CD8^+$ T cells from five CeD patients upon gliadin stimulation. **P*<0.05, ***P*<0.01, Friedman test followed by multiple comparisons test.

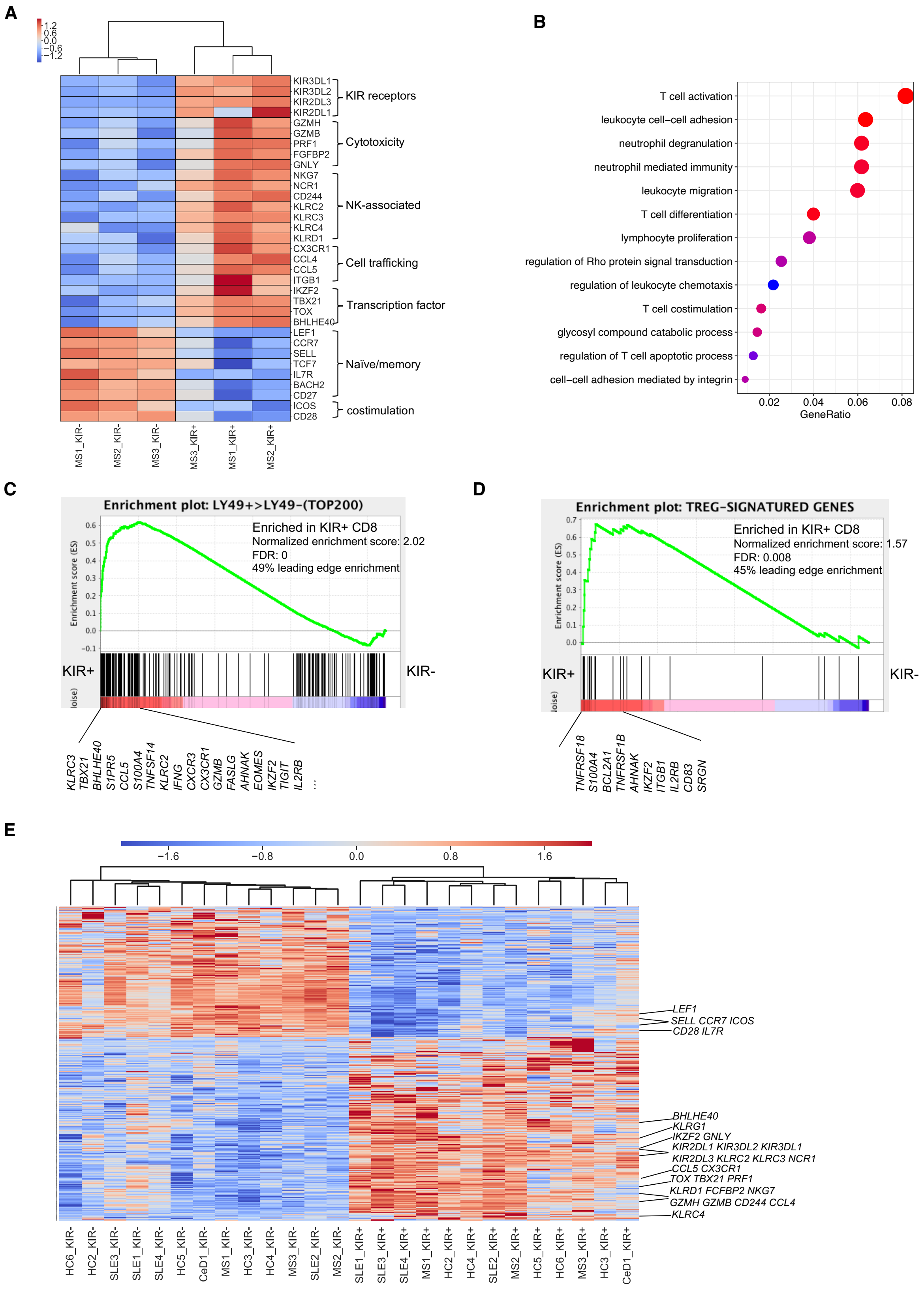
A
KIR receptors
KIR3DL1 KIR3DL2 KIR2DL3 KIR2DL1
Cytotoxicity
GZMH GZMB PRF1 FGFBP2 GNLY
NK-associated
NKG7 NCR1 CD244 KLRC2 KLRC3 KLRC4 KLRD1
Cell trafficking
CX3CR1 CCL4 CCL5 ITGB1
Transcription factor
IKZF2 TBX21 TOX BHLHE40
Naïve/memory
LEF1 CCR7 SELL TCF7 IL7R BACH2 CD27
costimulation
ICOS CD28
MS1_KIR- MS2_KIR- MS3_KIR- MS3_KIR+ MS1_KIR+ MS2_KIR+
B
T cell activation
leukocyte cell−cell adhesion
neutrophil degranulation
neutrophil mediated immunity
leukocyte migration
T cell differentiation
lymphocyte proliferation
regulation of Rho protein signal transduction
regulation of leukocyte chemotaxis
T cell costimulation
glycosyl compound catabolic process
regulation of T cell apoptotic process
cell−cell adhesion mediated by integrin
GeneRatio
Count
p.adjust
C
Enrichment plot: LY49+>LY49-(TOP200)
Enriched in KIR+ CD8
Normalized enrichment score: 2.02
FDR: 0
49% leading edge enrichment
Enrichment score (ES)
KIR+
KIR-
KLRC3 TBX21 BHLHE40 S1PR5 CCL5 S100A4 TNFSF14 KLRC2 IFNG CXCR3 CX3CR1 GZMB FASLG AHNAK EOMES IKZF2 TIGIT IL2RB ...
D
Enrichment plot: TREG-SIGNATURED GENES
Enriched in KIR+ CD8
Normalized enrichment score: 1.57
FDR: 0.008
45% leading edge enrichment
Enrichment score (ES)
KIR+
KIR-
TNFRSF18 S100A4 BCL2A1 TNFRSF1B AHNAK IKZF2 ITGB1 IL2RB CD83 SRGN
E
LEF1
SELL CCR7 ICOS
CD28 IL7R
BHLHE40
KLRG1
IKZF2 GNLY
KIR2DL1 KIR3DL2 KIR3DL1
KIR2DL3 KLRC2 KLRC3 NCR1
CCL5 CX3CR1
TOX TBX21 PRF1
KLRD1 FCFBP2 NKG7
GZMH GZMB CD244 CCL4
KLRC4
HC6_KIR- HC2_KIR- SLE3_KIR- SLE1_KIR- SLE4_KIR- HC5_KIR- CeD1_KIR- MS1_KIR- HC3_KIR- HC4_KIR- MS3_KIR- SLE2_KIR- MS2_KIR- SLE1_KIR+ SLE3_KIR+ SLE4_KIR+ MS1_KIR+ HC2_KIR+ HC4_KIR+ SLE2_KIR+ MS2_KIR+ HC5_KIR+ HC6_KIR+ MS3_KIR+ HC3_KIR+ CeD1_KIR+

Fig. S3. Bulk RNA-seq analysis of KIR$^+$ versus KIR$^-$ CD8$^+$ T cells. (**A**) Heatmap displaying transcript levels of selected differentially expressed genes (DEGs) in KIR$^+$ vs. KIR$^-$ CD8$^+$ T cells sorted from MS patients (N=3). (**B**) Gene Ontology (GO) enrichment analysis of DEGs between KIR$^+$ and KIR$^-$ CD8$^+$ T cells from MS patients. **C**-**D**, Gene set enrichment analysis (GSEA) on KIR$^+$ versus KIR$^-$ CD8$^+$ T cells from MS patients using the top 200 genes up-regulated in Ly49$^+$ CD8$^+$ T cells compared to Ly49$^-$ cells (**C**) or signature genes of CD4$^+$ regulatory T (Treg) cells (**D**) as reference gene sets. (**E**) Heatmap showing expression of the 963 DEGs in KIR$^+$ and KIR$^-$ CD8$^+$ T cells from healthy subjects and patients with autoimmune diseases (MS, CeD or SLE) determined by bulk RNA-seq. Genes overlapping with DEGs defined in MS patients are annotated.

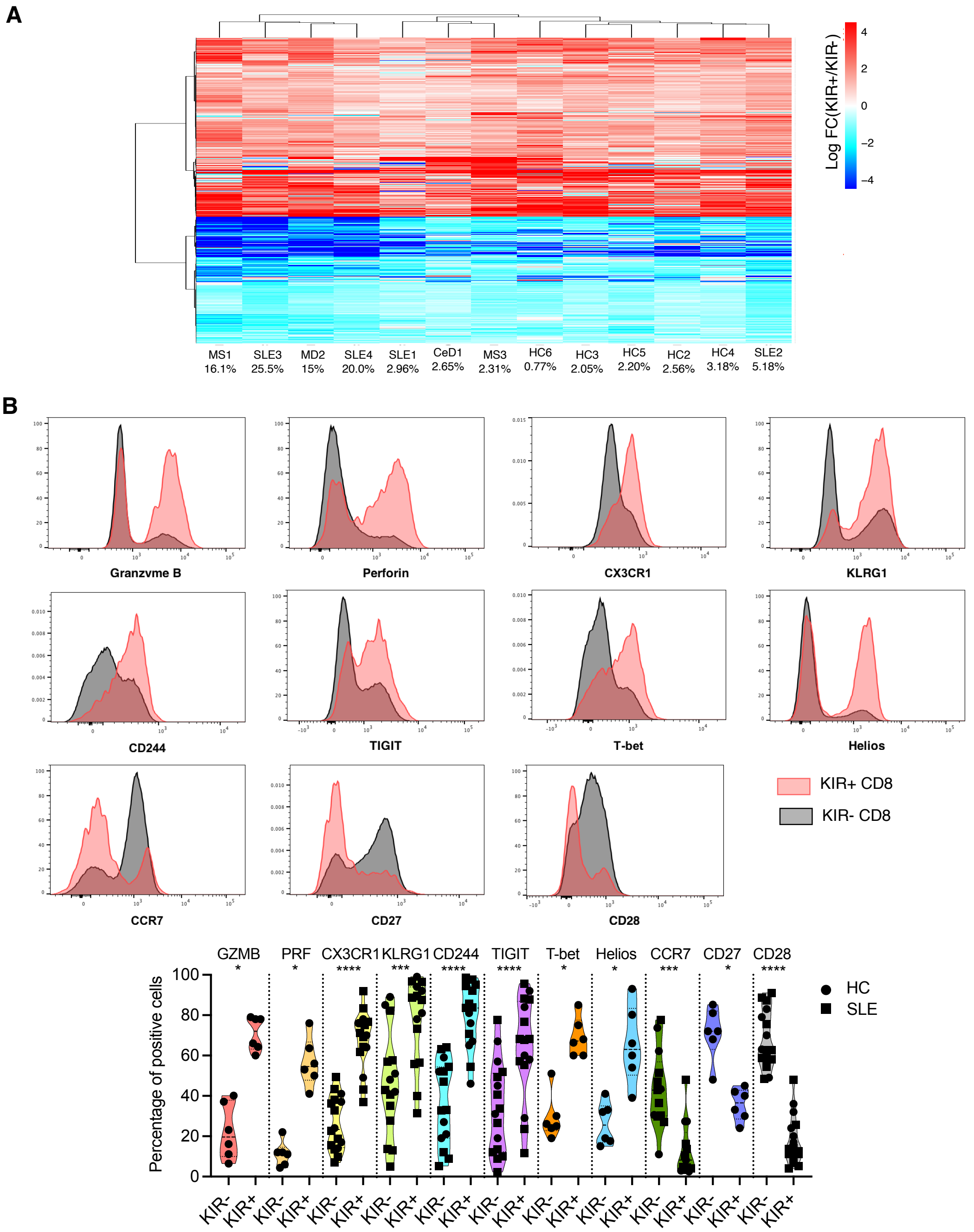

Fig. S4. Phenotypic analysis of KIR$^+$ versus KIR$^-$ CD8$^+$ T cells in the blood. (**A**) Heatmap showing fold changes of the 963 DEGs between KIR$^+$ and KIR$^-$ CD8$^+$ T cells from all individuals. Columns show samples annotated with the frequency of KIR$^+$CD8$^+$ T cells in total CD8$^+$ T cells, and rows and columns are ordered based on hierarchical clustering. Normalized fold changes are centered for each gene. (**B**) Protein expression of featured DEGs (Granzyme B (GZMB), perforin (PRF), CX3CR1, KLRG1, CD244, TIGIT, T-bet, Helios, CCR7, CD27 and CD28) in KIR$^+$ and KIR$^-$ CD8$^+$ T cells from the blood of healthy controls (HC) and patients with systemic lupus erythematosus (SLE) measured by flow cytometry. *P<0.05, ***P<0.001, ****P<0.0001, Wilcoxon matched-pairs signed rank test.

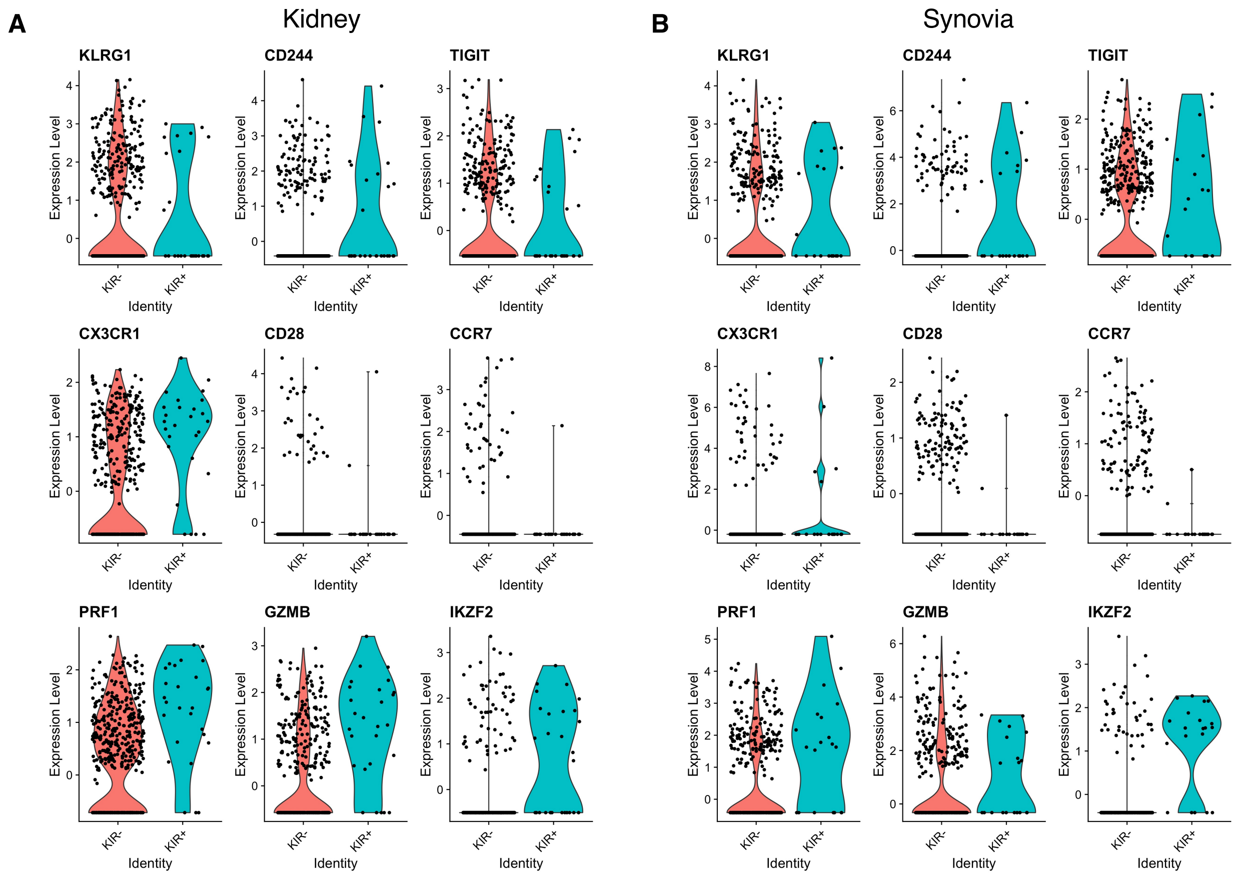

Fig. S5. Phenotypic analysis of KIR^{+}CD8^{+} T cells in inflamed tissues. KIR^{+} versus KIR^{-} CD8^{+} T cells in the kidney of SLE patients or synovial tissues from rheumatoid arthritis (RA) were analyzed using the single cell RNA-seq data generated by AMP RA/SLE program. The UMI count matrix was imported and CD8^{+} T cells (expressing *CD3E*, *CD8A* and *CD8B* transcripts) were selected for further analysis. Violin plots showing expression of *KLRG1*, *CD244*, *TIGIT*, *CX3CR1*, *CD28*, CCR7, *PRF1*, *GZMB* and *IKZF2* transcripts in KIR^{+} (expressing any of the *KIR* transcripts: *KIR3DL1*, *KIR2DL3*, *KIR2DL2*, *KIR2DL1* or *KIR3DL2*) versus KIR^{-} CD8^{+} T cells in the SLE kidney (**A**) and RA synovia (**B**).

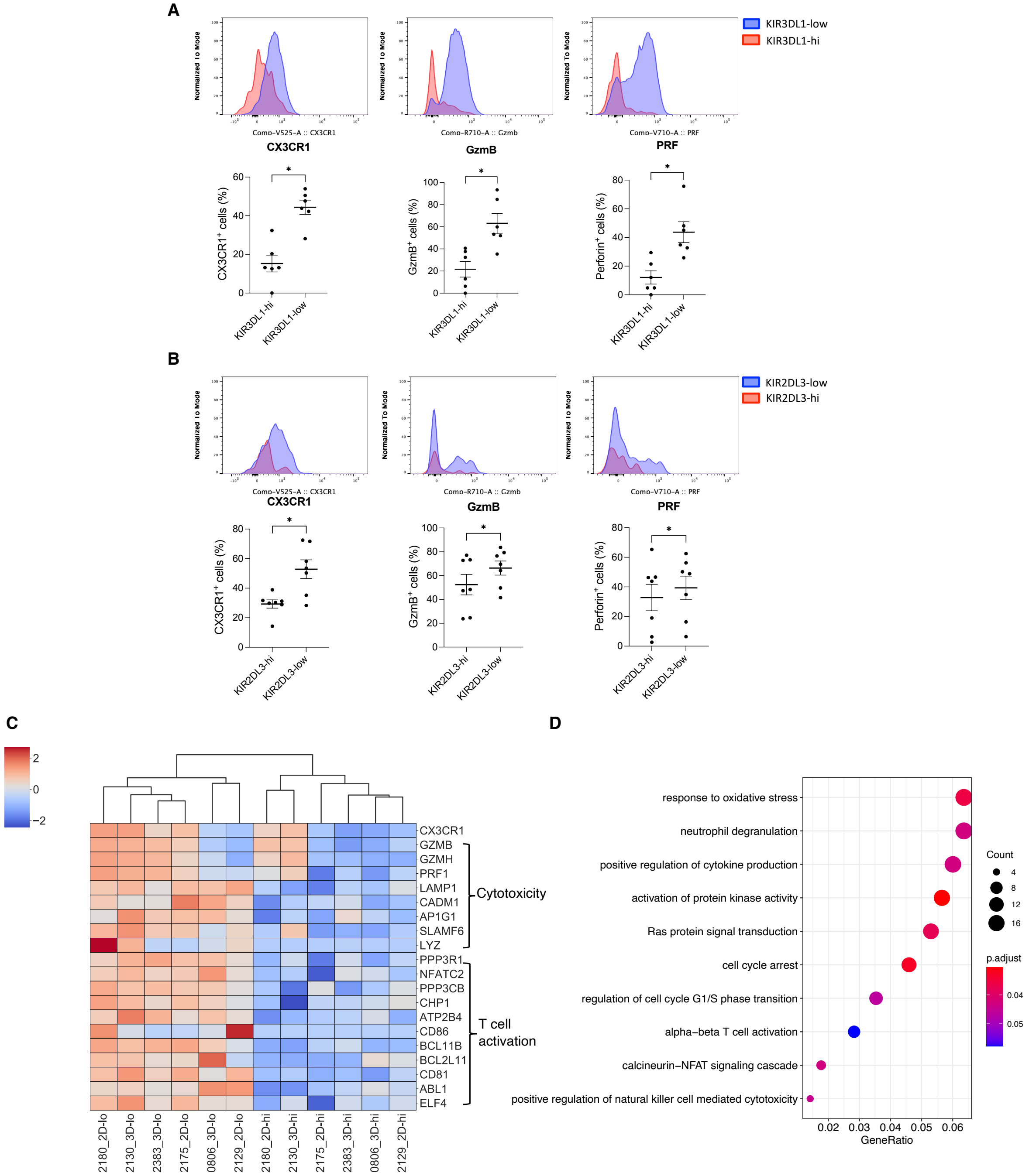

Fig. S6. Comparison of KIR^{hi} versus KIR^{lo} CD8$^+$ T cells in the blood. (**A**) Expression of surface CX3CR1 and intracellular granzyme B and perforin in CD8$^+$ T cells with high and low KIR3DL1 expression from PBMCs of healthy subjects (N=7). **P*<0.05, Wilcoxon matched-pairs signed rank test. (**B**) Expression of surface CX3CR1 and intracellular granzyme B and perforin in CD8$^+$ T cells with high and low KIR2DL2/3 expression from PBMCs of healthy subjects (N=7). **P*<0.05, Wilcoxon matched-pairs signed rank test. (**C**) Heatmap displaying transcript levels of selected differentially expressed genes (DEGs) in KIR3DL1^{hi} or KIR2DL2/3^{hi} vs. KIR3DL1^{lo} or KIR2DL2/3^{lo} CD8$^+$ T cells sorted from healthy subjects (N=6). (**D**) Gene Ontology (GO) enrichment analysis of genes upregulated in KIR3DL1^{hi} or KIR2DL2/3^{hi} CD8$^+$ T cells.

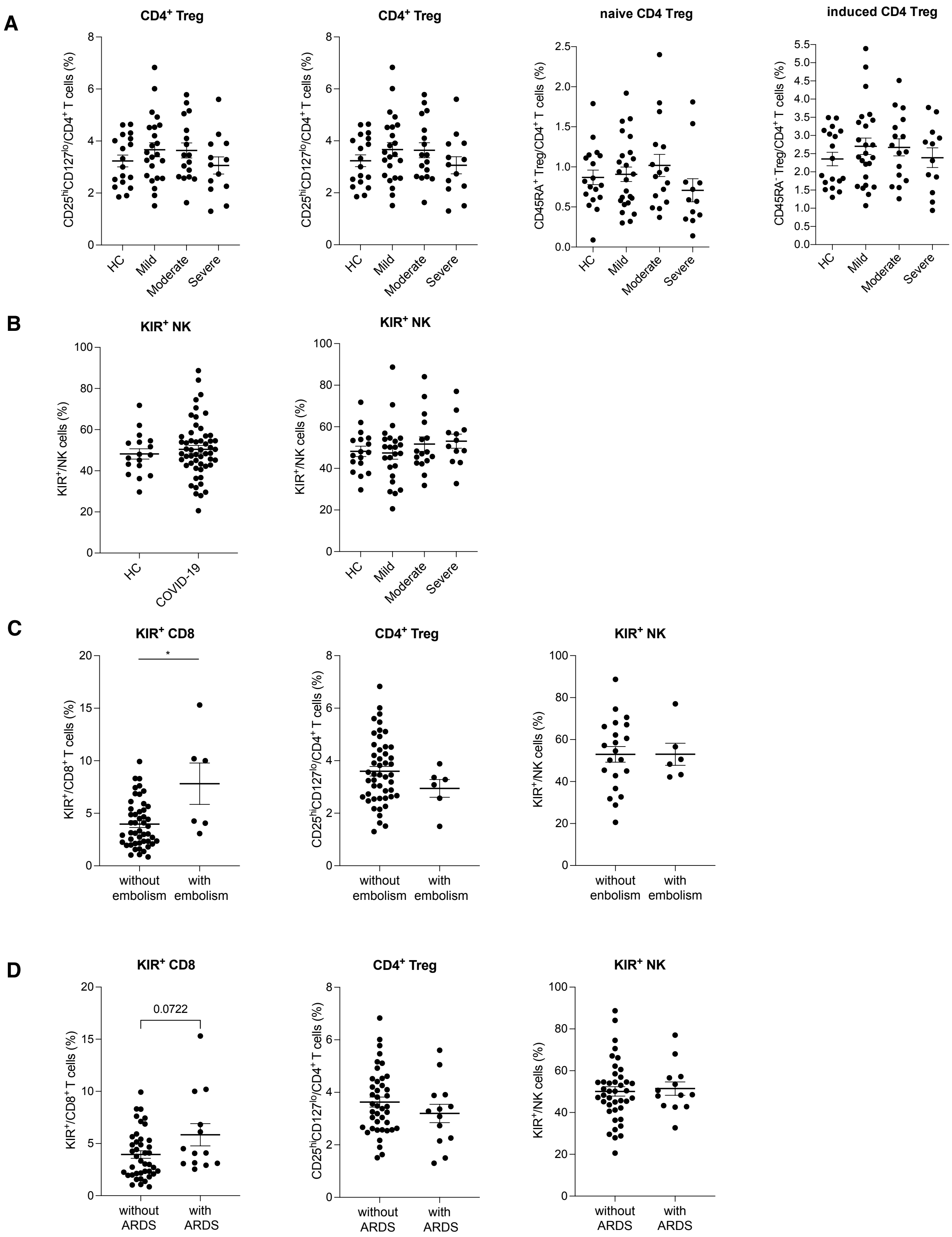
A
CD4+ Treg
CD25hiCD127lo/CD4+ T cells (%)
naive CD4 Treg
CD45RA+ Treg/CD4+ T cells (%)
induced CD4 Treg
CD45RA- Treg/CD4+ T cells (%)
HC
Mild
Moderate
Severe
B
KIR+ NK
KIR+/NK cells (%)
COVID-19
C
KIR+ CD8
KIR+/CD8+ T cells (%)
*
without embolism
with embolism
without enbolism
D
0.0722
without ARDS
with ARDS

Fig. S7. Flow cytometric analysis of PBMCs from COVID-19 patients. (**A**) Summary histograms showing the frequency of $CD4^+$ regulatory T cells ($CD25^{hi}CD127^{lo}$) as well as subpopulations of $CD4^+$ Tregs in the peripheral blood of healthy controls (HC, N=17) and COVID-19 patients with varying disease severity (Mild: 23, Moderate: 17, Severe: 13). (**B**) Summary histograms showing the frequency of KIR^+ NK cells in the peripheral blood of healthy controls (HC, N=17) and COVID-19 patients with varying disease severity (Mild: 23, Moderate: 17, Severe: 13). (**C**) Frequency of KIR^+CD8^+ T cells, $CD4^+$ Tregs ($CD25^{hi}CD127^{lo}$) and KIR^+ NK cells in COVID-19 patients with or without embolism. *$P<0.05$, Mann–Whitney test. (**D**) Frequency of KIR^+CD8^+ T cells, $CD4^+$ Tregs ($CD25^{hi}CD127^{lo}$) and KIR^+ NK cells in COVID-19 patients with or without ARDS. Mann–Whitney test.

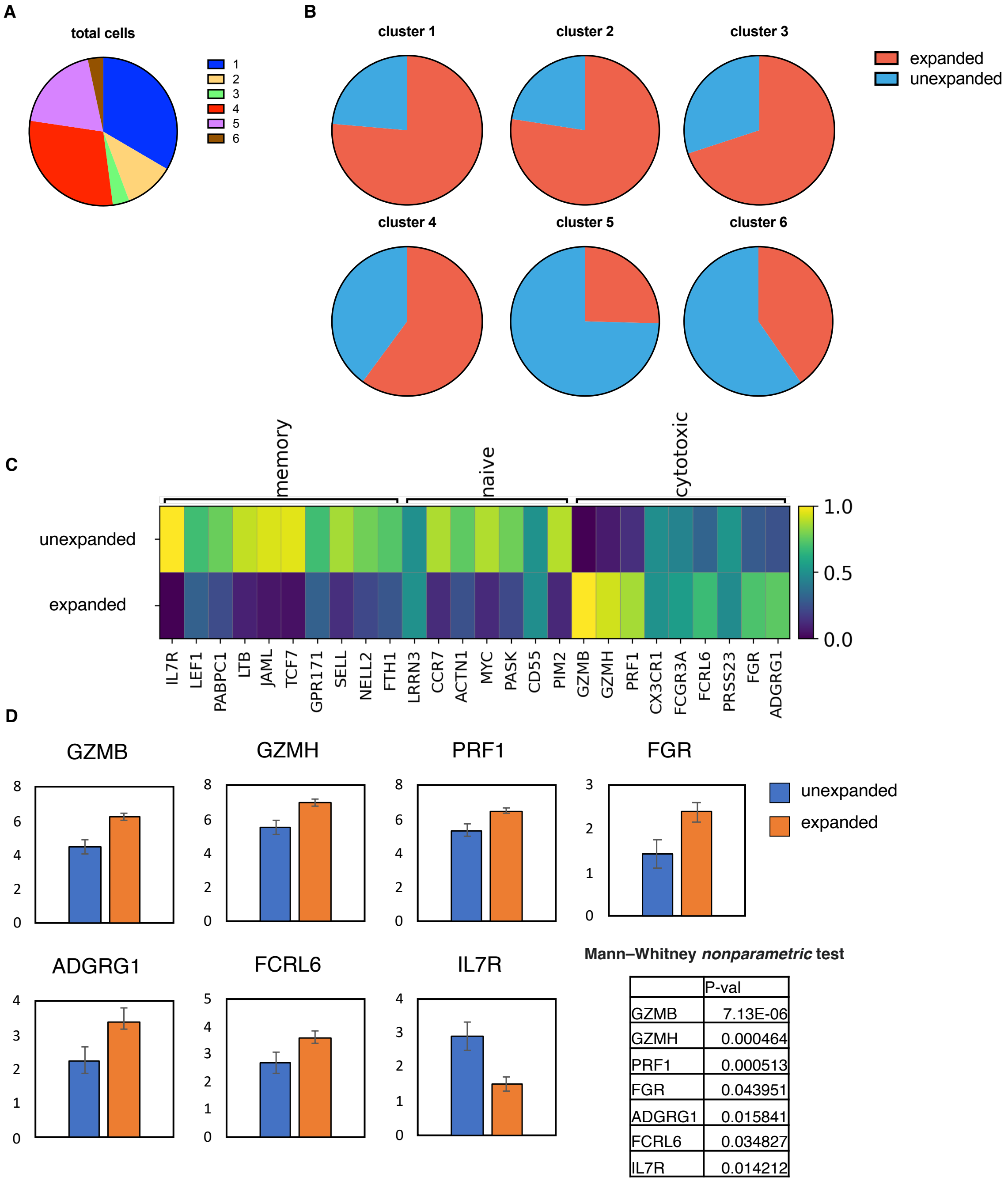

	P-val
GZMB	7.13E-06
GZMH	0.000464
PRF1	0.000513
FGR	0.043951
ADGRG1	0.015841
FCRL6	0.034827
IL7R	0.014212

Fig. S8. Single cell RNA-seq analysis of KIR$^+$CD8$^+$ T cells. (**A**) Pie chart depicting the composition of the six clusters identified by unsupervised clustering in total KIR$^+$CD8$^+$ T cells. (**B**) Pie charts showing the composition of expanded and unexpanded KIR$^+$CD8$^+$ T cells in each cluster. **C**-**D**, Single-cell RNA-seq analysis of expanded versus unexpanded KIR$^+$CD8$^+$ T cells from COVID-19 patients by 10X Genomics. (**C**) Heatmap displaying normalized expression of naïve-, memory- or cytotoxic-associated genes in unexpanded versus expanded KIR$^+$CD8$^+$ T cells from the blood of COVID-19 patients. (**D**) Bar graphs showing mRNA expression of *GZMB*, *GZMH*, *PRF1*, *FGR, ADGRG1, FCRL6* and *IL7R* in unexpanded and expanded KIR$^+$CD8$^+$ T cells in the peripheral blood from COVID-19 patients.

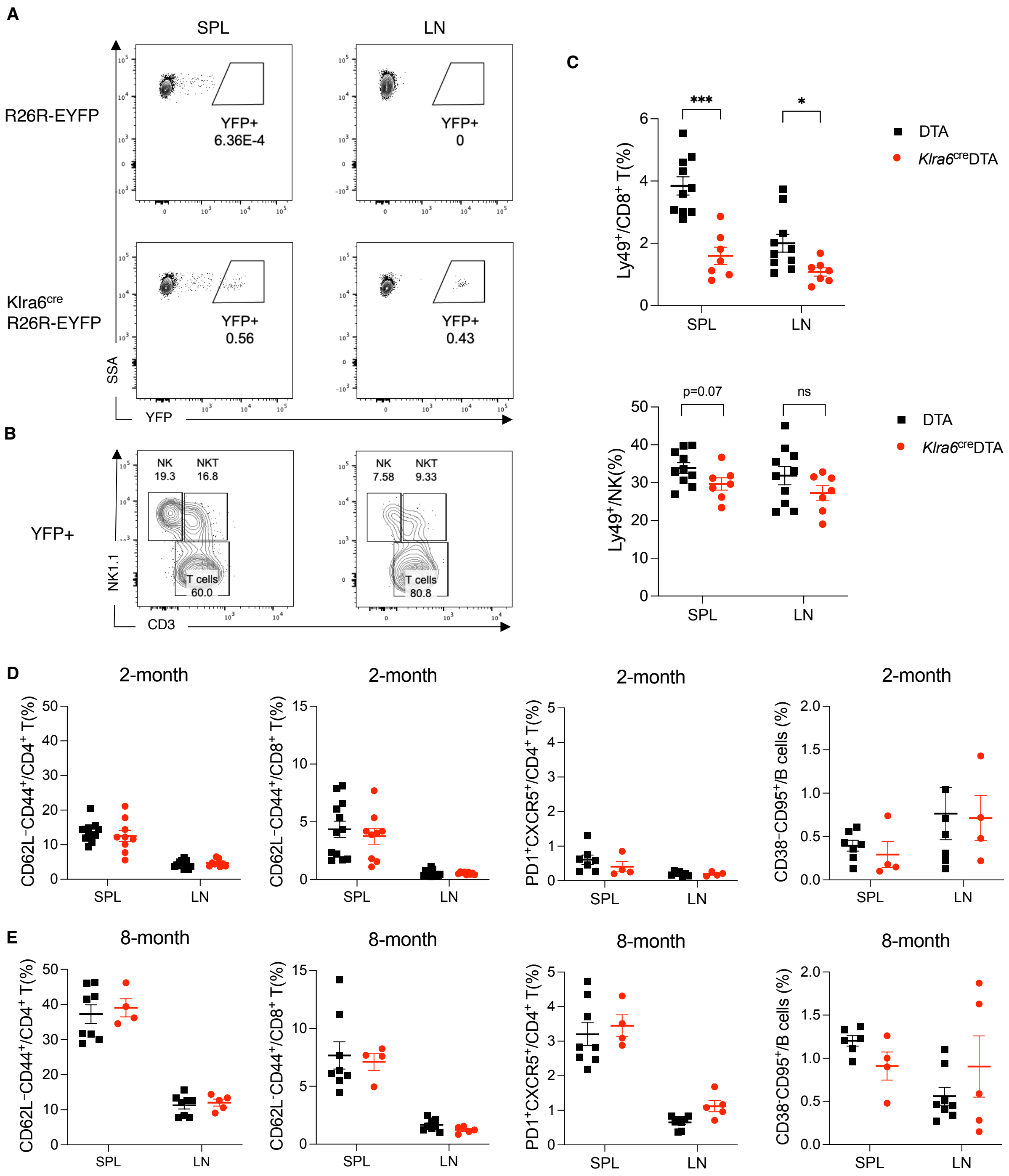

A
SPL
LN
R26R-EYFP
YFP+
6.36E-4
YFP+
0
Klra6cre
R26R-EYFP
YFP+
0.56
YFP+
0.43
SSA
YFP
B
YFP+
NK
19.3
NKT
16.8
T cells
60.0
NK
7.58
NKT
9.33
T cells
80.8
NK1.1
CD3
C
***
*
Ly49+/CD8+ T(%)
SPL
LN
DTA
Klra6creDTA
p=0.07
ns
Ly49+/NK(%)
D
2-month
CD62L-CD44+/CD4+ T(%)
CD62L-CD44+/CD8+ T(%)
PD1+CXCR5+/CD4+ T(%)
CD38-CD95+/B cells (%)
E
8-month

Fig. S9. Characterization of *Klra6*^{cre}R26R-EYFP and *Klra6*^{cre}DTA mice. (**A**) Representative FACS plots showing the frequency of YFP^+ cells in the spleen and lymph nodes of *Klra6*^{cre}R26R-EYFP mice. (**B**) Representative plots showing the proportion of $CD3^+$ or $NK1.1^+$ cells among YFP^+ cells from the spleen and lymph nodes of *Klra6*^{cre}R26R-EYFP mice. (**C**) Summary histograms of the frequency of $Ly49^+CD8^+$ T cells and $Ly49^+$ NK cells in the spleen and lymph nodes of 2-month old DTA mice (N=10) and *Klra6*^{cre}DTA mice (N=7). **P*<0.05, ****P*<0.001, Mann–Whitney test. (**D**) Summary graphs showing the frequency of $CD62L^-CD44^+$ effector $CD8^+$ T cells, $CD62L^-CD44^+$ effector $CD4^+$ T cells, $PD\text{-}1^+CXCR5^+CD4^+$ T cells (Tfh) and $CD38^-CD95^+$ B (Germinal Center B) cells in the spleen and lymph nodes of 2-month old DTA mice (N=12 or 7) and *Klra6*^{cre}DTA mice (N=9 or 4). (**E**) Summary graphs showing the frequency of $CD62L^-CD44^+$ effector $CD8^+$ T cells, $CD62L^-CD44^+$ effector $CD4^+$ T cells, $PD\text{-}1^+CXCR5^+CD4^+$ T cells (Tfh) and $CD38^-CD95^+$ B (Germinal Center B) cells in the spleen and lymph nodes of 8-month old DTA mice (N=5) and *Klra6*^{cre}DTA mice (N=8). Representative data from two independent experiments are shown. The mean±SEM is indicated.

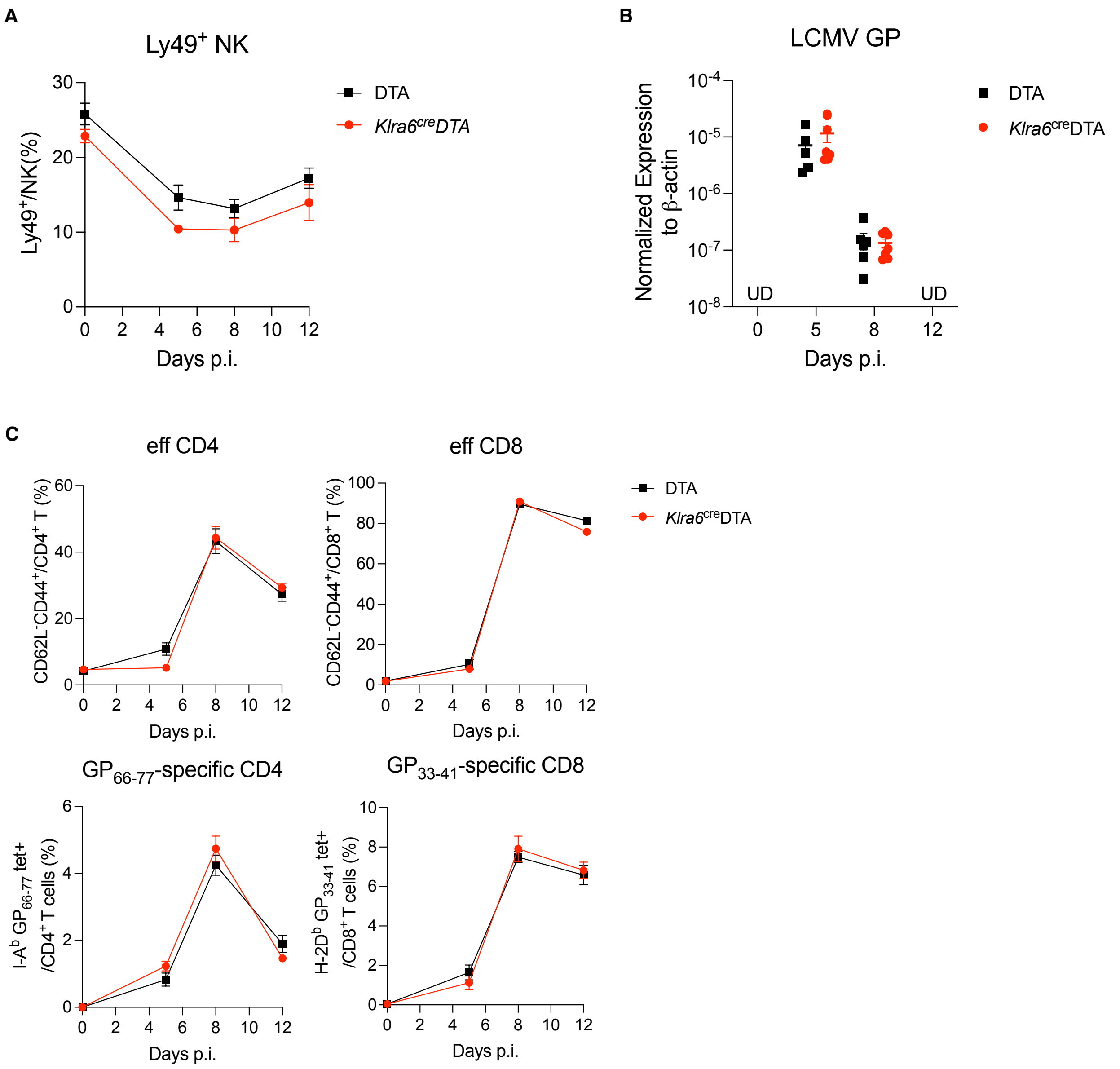

Fig. S10. Analysis of *Klra6*^{cre}DTA mice versus DTA mice upon LCMV-Armstrong infection. (**A**) Frequency of Ly49^{+} NK cells in the blood of DTA mice (N=8) and *Klra6*^{cre}DTA mice (N=5) 0, 5, 8 and 12 days post LCMV-Armstrong infection. (**B**) Summary graph showing relative expression of LCMV GP (normalized to β-actin) in the blood of DTA mice (N=7) and *Klra6*^{cre}DTA mice (N=6) 0, 5, 8 and 12 days post LCMV-Armstrong infection as measured by RT-qPCR. (**C**) Percentage of CD62L^{-}CD44^{+} effector CD8^{+} T cells, CD62L^{-}CD44^{+} effector CD4^{+} T cells, I-A^{b} GP$_{66\text{-}77}$ tetramer positive CD4^{+} T cells and H-2D^{b} GP$_{33\text{-}41}$ tetramer positive CD8^{+} T cells in the blood of DTA mice (N=8) and *Klra6*^{cre}DTA mice (N=5) 0, 5, 8 and 12 days post LCMV-Armstrong infection. Representative data from two independent experiments are shown. The mean±SEM is indicated.

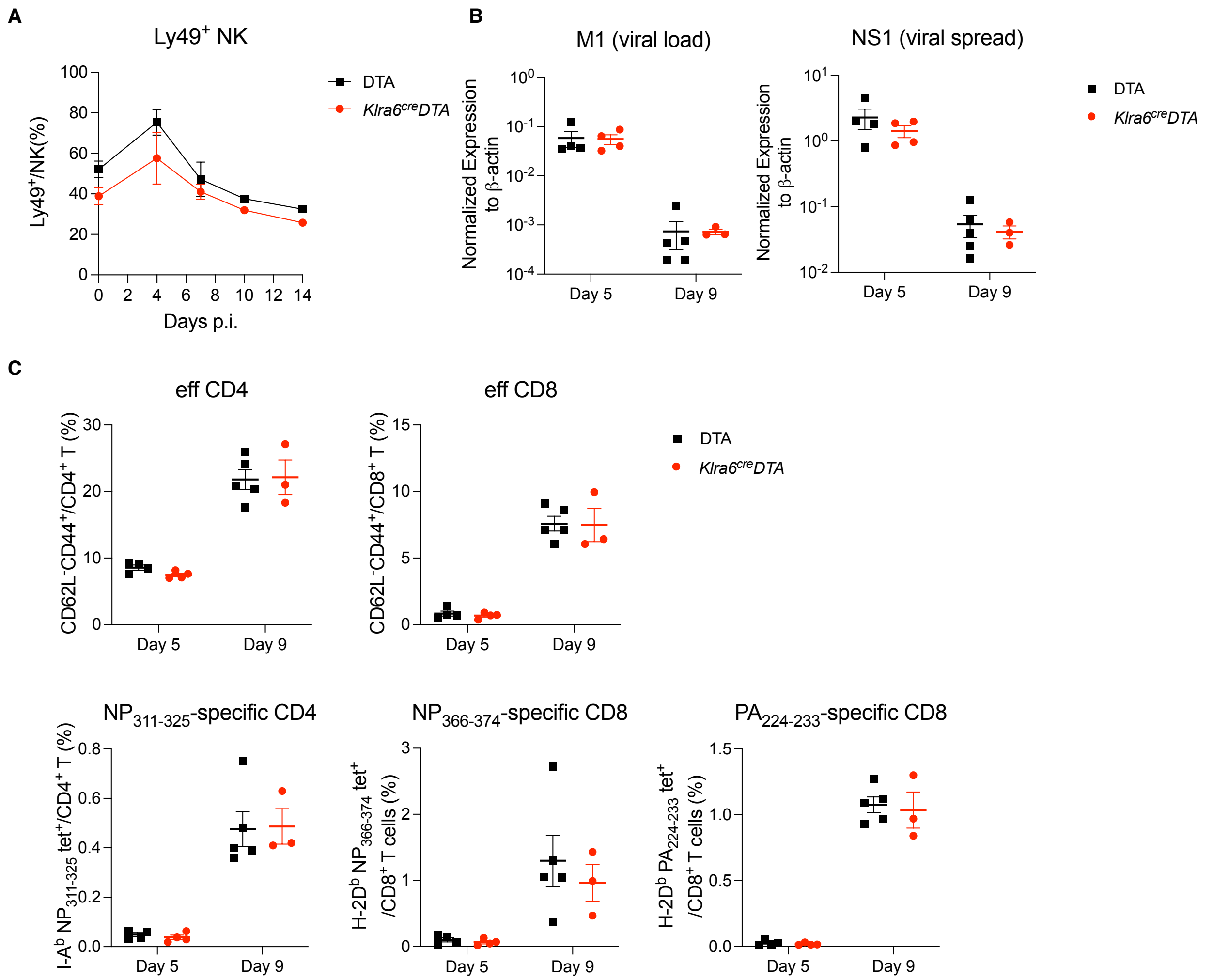

Fig. S11. Analysis of *Klra6*^{cre}DTA mice versus DTA mice upon influenza A/PR/8/34 H1N1 infection. (**A**) Frequency of Ly49^{+} NK cells in the blood of *Klra6*^{cre}DTA mice (N=5) and DTA mice (N=6) after influenza infection. (**B**) Summary graphs showing relative expression of M1 (representing viral load) and NS1 (representing viral spread) normalized to β-actin in the lung of DTA mice (N=4 or 5) and *Klra6*^{cre}DTA mice (N=3 or 4) on Day 5 and 9 post influenza infection as measured by RT-qPCR. (**C**) Percentage of CD62L^{-}CD44^{+} effector CD8^{+} T cells, CD62L^{-}CD44^{+} effector CD4^{+} T cells, I-A^{b} NP$_{311-325}$ tetramer positive CD4^{+} T cells, H-2D^{b} NP$_{366-374}$ tetramer positive CD8^{+} T cells and H-2D^{b} PA$_{224-233}$ tetramer positive CD8^{+} T cells in the lung-draining lymph nodes of DTA mice (N=4 or 5) and *Klra6*^{cre}DTA mice (N=3 or 4) on Day 5 and 9 post influenza infection. Representative data from two independent experiments are shown. The mean±SEM is indicated.
