# Supplementary material for: KIR+CD8+ T cells suppress pathogenic T cells and are active in autoimmune diseases and COVID-19
Source: Science. 2022 Mar 8;376(6590):eabi9591. doi: 10.1126/science.abi9591 (PMC8995031; doi:10.1126/science.abi9591)
Supplement: Supplementary file 3 — Tables S1 to S7 [file science.abi9591_tables_s1_to_s7.zip › science.abi9591_table_captions.pdf]

**Table S1. (separate file)**
Differentially expressed genes between $KIR^+$ and $KIR^-$ CD8+ T cells from MS patients

**Table S2. (separate file)**
Differentially expressed genes between $KIR^+$ and $KIR^-$ CD8+ T cells from healthy donors and a variety of autoimmune diseases (MS, SLE and CeD)

**Table S3. (separate file)**
Differentially expressed features of each cluster compared to all other cells among $CD8^+$ T cells from healthy controls, MS patients and COVID-19 patients (10x Genomic platform)

**Table S4. (separate file)**
Differentially expressed features of each cluster compared to all other cells among $KIR^+CD8^+$ T cells from healthy subjects and patients with autoimmune diseases (Smart-seq2 platform)

**Table S5. (separate file)**
Detailed information of patients and healthy controls included in the study

**Table S6. (separate file)**
Clinical metadata of COVID-19 patients and healthy controls included in the study

**Table S7. (separate file)**
Fluorescent dye-conjugated antibodies used for flow cytometric analysis.
