# Supplementary material for: KIR+CD8+ T cells suppress pathogenic T cells and are active in autoimmune diseases and COVID-19
Source: Science. 2022 Mar 8;376(6590):eabi9591. doi: 10.1126/science.abi9591 (PMC8995031; doi:10.1126/science.abi9591)
Supplement: Supplementary file 4 — MDAR Reproducibility Checklist [file science.abi9591_mdar_reproducibility_checklist.pdf]

Materials Design Analysis Reporting (MDAR) Checklist for Authors

The MDAR framework establishes a minimum set of requirements in transparent reporting applicable to studies in the life sciences (see Statement of Task: doi:10.31222/osf.io/9sm4x.). The MDAR checklist is a tool for authors, editors and others seeking to adopt the MDAR framework for transparent reporting in manuscripts and other outputs. Please refer to the MDAR Elaboration Document for additional context for the MDAR framework.

Materials

Antibodies	Yes (indicate where provided: page no/section/legend)	n/a
For commercial reagents, provide supplier name, catalogue number and RRID, if available.	table S7	

Cell materials	Yes (indicate where provided: page no/section/legend)	n/a
**Cell lines:** Provide species information, strain. Provide accession number in repository **OR** supplier name, catalog number, clone number, **OR** RRID		✓
**Primary cultures:** Provide species, strain, sex of origin, genetic modification status.	Page 18 and table S5	

Experimental animals	Yes (indicate where provided: page no/section/legend)	n/a
**Laboratory animals:** Provide species, strain, sex, age, genetic modification status. Provide accession number in repository **OR** supplier name, catalog number, clone number, **OR** RRID	Page 19-20	
**Animal observed in or captured from the field:** Provide species, sex and age where possible		✓
**Model organisms:** Provide Accession number in repository (where relevant) **OR** RRID		✓

Plants and microbes	Yes (indicate where provided: page no/section/legend)	n/a
**Plants:** provide species and strain, unique accession number if available, and source (including location for collected wild specimens)		✓
**Microbes:** provide species and strain, unique accession number if available, and source	Page 27	

Human research participants	Yes (indicate where provided: page no/section/legend)	n/a
Identify authority granting ethics approval (IRB or equivalent committee(s), provide reference number for approval.	Page 18-19	
Provide statement confirming informed consent obtained from study participants.	Page 18-19	
Report on age and sex for all study participants.	Tables S5 and S6	

Design

**Study protocol**	**Yes (indicate where provided: page no/section/legend)**	**n/a**
For clinical trials, provide the trial registration number **OR** cite DOI in manuscript.	Page 19	

**Laboratory protocol**	**Yes (indicate where provided: page no/section/legend)**	**n/a**
Provide DOI or other citation details if detailed step-by-step protocols are available.	Page 24-25	

**Experimental study design (statistics details)**	**Yes (indicate where provided: page no/section/legend)**	**n/a**
State whether and how the following have been done**, or** if they were not carried out.		
Sample size determination	Page 28	
Randomisation		✓
Blinding		✓
Inclusion/exclusion criteria		✓

**Sample definition and in-laboratory replication**	**Yes (indicate where provided: page no/section/legend)**	**n/a**
State number of times the experiment was replicated in laboratory	Figure legends	
Define whether data describe technical or biological replicates	Figure legends	

**Ethics**	**Yes (indicate where provided: page no/section/legend)**	**n/a**
Studies involving human participants: State details of authority granting ethics approval (IRB or equivalent committee(s), provide reference number for approval.	Page 18-19	
Studies involving experimental animals: State details of authority granting ethics approval (IRB or equivalent committee(s), provide reference number for approval.	Page 19-20	
Studies involving specimen and field samples: State if relevant permits obtained, provide details of authority approving study; if none were required, explain why.		✓

**Dual Use Research of Concern (DURC)**	**Yes (indicate where provided: page no/section/legend)**	**n/a**
If study is subject to dual use research of concern, state the authority granting approval and reference number for the regulatory approval		✓

Analysis

Attrition	Yes (indicate where provided: page no/section/legend)	n/a
State if sample or data point from the analysis is excluded, and whether the criteria for exclusion were determined and specified in advance.	Page 28	

Statistics	Yes (indicate where provided: page no/section/legend)	n/a
Describe statistical tests used and justify choice of tests.	Page 28 and figure legends	

Data Availability	Yes (indicate where provided: page no/section/legend)	n/a
State whether newly created datasets are available, including protocols for access or restriction on access.	Page 36	
If data are publicly available, provide accession number in repository or DOI or URL.	Page 36	
If publicly available data are reused, provide accession number in repository or DOI or URL, where possible.	Page 23	

Code Availability	Yes (indicate where provided: page no/section/legend)	n/a
For all newly generated code and software essential for replicating the main findings of the study:		
State whether the code or software is available.	Page 36	
If code is publicly available, provide accession number in repository, or DOI or URL.	Page 36	

Reporting

Adherence to community standards	Yes (indicate where provided: page no/section/legend)	n/a
MDAR framework recommends adoption of discipline-specific guidelines, established and endorsed through community initiatives. Journals have their own policy about requiring specific guidelines and recommendations to complement MDAR.		
State if relevant guidelines (eg., ICMJE, MIBBI, ARRIVE) have been followed, and whether a checklist (eg., CONSORT, PRISMA, ARRIVE) is provided with the manuscript.		✓
